# “A better system” – managing the adverse effects of prescribed medicines: Cluster-randomised controlled trial and process evaluation of the ADRe Profile in primary care

**DOI:** 10.1371/journal.pone.0356480

**Published:** 2026-09-18

**Authors:** Vera Logan, Alex Conboy, Neil Carter, David Hughes, Adam Turner, Alan Watkins, Sue Jordan

**Affiliations:** Faculty of Medicine, Health and Life Science, Swansea University, Swansea, Wales, United Kingdom; University of South Australia, AUSTRALIA

## Abstract

**Introduction:**

The avoidable harm due to incomplete monitoring of repeat prescriptions in primary care has been known for decades, but prevention has proved difficult. We aimed to test the ability of the Adverse Drug Reaction (ADRe) Profile to identify and address clinical problems for patients aged ≥65 prescribed >4 long-term medicines, living in their own homes, and managed in primary care.

**Methods:**

A pragmatic cluster-randomized controlled trial was undertaken in 6 primary care practices in South-West Wales, alongside a process evaluation. In addition to usual care, patients in the intervention arm completed the ADRe Profile, which was then shared with the practice pharmacist. Patients completed a second ADRe Profile with the nurse researcher 20 weeks later. The control arm received usual care. Information on patients’ clinical problems and prescriptions was extracted from practice medical records before and after the intervention and analysed using multilevel generalised linear mixed models (GLMM), including practice as a random intercept, and accounting for trial arm, age, deprivation and co-morbidities. Symptoms recorded on the first and second Profiles were compared. Resolution of clinical problems was described, and participants’ perspectives sought.

**Results:**

We recruited one general practice group, comprising six practices, and 60 patients. Three patients were lost to the study. Intervention arm patients were more likely to have more than one problem addressed than control arm patients (22/27[82%] vs. 11/30[35%], adjusted odds ratio [aOR] 7.48, 95% confidence interval [CI] 1.99-28.10). All intervention arm patients either benefited or expected to benefit following referrals. For two patients, the intervention proved critical: detection of B12 deficiency in a patient using metformin and flecainide de-prescribing. Fewer patients reported pain by the study’s end (16/27 vs 24/27). All patients were grateful for ADRe, but clinicians had reservations concerning staff time.

**Conclusion:**

The ADRe Profile benefited patients by reducing risk of suboptimal prescribing and iatrogenic harm. The project is registered with the Clinicaltrials.gov as NCT04663360 (https://clinicaltrials.gov/study/NCT04663360).

## Background

The avoidable harm due to incomplete monitoring of repeated prescriptions has been documented for decades [1–4], and has proved intractable [5–8]. Despite the World Health Organisation (WHO) global challenge initiative [9,10], mortality due to adverse drug events (ADEs) rose from 2.05 (0.92–3.18) per 100,000 population in 2001 to 6.86 (5.76–7.95) in 2019, attributed, in the main, to unmonitored primary care prescribing [11]. Most adverse drug reactions (ADRs) and ADEs are avoidable [9,12–14]. The full extent of harm is uncertain, but known associations require close monitoring, as some adverse effects, including tinnitus and ototoxicity associated with antidepressants [15], and dementia associated with pregabalin, atorvastatin and oxycodone [16], are irreversible. Up to 20.37% (95% CI 16.89, 23.85) of primary care patients experience ADRs [17]. (Definitions in glossary, below.)

Polypharmacy, defined as prescription of 5 or more regular medicines [9,18,19], increases the potential for ADRs [20,21], errors [22], and drug-related hospital admissions [23,24]. The adverse effects of polypharmacy disproportionately impact the old and the poor [25–27]. Both the Royal Pharmaceutical Society’s (RPS’s) medicines optimisation framework [28], and the WHO’s approach depend upon monitoring and evaluating the effects of medicines on patients’ outcomes. Methods currently employed for ADR identification and mitigation include voluntary reporting and reviews of prescription charts, often relying on pre-emptive lists of potentially inappropriate medicines [29]. There is little evidence that existing interventions have improved outcomes for poly-medicated older adults [30]. Single-profession initiatives have not always involved patients’ reports of real-time problems and have not reduced hospital admissions or problems such as falls [31–39], particularly in primary care [40]. Whilst prescribing improved, outcomes did not; despite convenience, standard approaches were ineffective [31,34].

Instruments identifying patients’ real-time, actual health problems that inform medicine reviews can work, particularly if they are comprehensive [41]. Our instrument, the ADRe Profile, integrates the expertise of nurses, pharmacists, prescribers, and patients in the surveillance and prevention of potential ADRs, ADEs and health problems [8,42–44]. ADRe [8,45,46] comprehensively and systematically compiles patients’ signs and symptoms in a single document, (see [46]). Patients, carers or nurses note problems that need attention and might be caused by prescription medicines, before sharing the document with pharmacists and prescribers. With information on patients’ real-time problems available from one source, pharmacists conduct their usual medicines reviews, recommending, signposting, adjusting prescriptions and referring where necessary. ADRe offers instant access information for each problem regarding assessments, referrals, and aetiologies. These include the medicines that may be contributing to signs and symptoms and conditions that may need to be managed more closely. Whilst digital prescribing systems identify errors and drug interactions, ADRe brings patients’ actual problems into focus, so that problems can be juxtaposed with prescriptions.

The impact of the ADRe Profile has been reported in care homes, mental health teams and outpatients in randomised controlled trials [43,47] and observational studies [8,48–51]. Across these studies, ADRe implementation consistently increased the identification and resolution of health problems, including pain. This paper reports on a randomised controlled trial (RCT) that assessed the impact of the ADRe Profile in general practice. The primary objective was to assess, at individual patient level, whether the ADRe Profile identified health problems that were or might be ameliorated by focusing on medicines’ optimisation. A feasibility study demonstrated that ADRe had potential to benefit patients in general practice [52]. We explored: differences between trial arms in problems and prescriptions in the practice medical records at baseline and follow-up (before and after the intervention was delivered in one arm); problems identified and addressed using ADRe; clinical gains emanating from these actions; costs of staff time; participants’ views; harms recorded.

The trial is registered (https://clinicaltrials.gov/study/NCT04663360).The protocol is published [44].

## Methods

### Patient and public involvement

The Swansea University Patient Experience and Evaluation in Research (PEER) group expressed its full support for the research and helped to develop the Participant Information Sheet. Two members joined the steering group.

### Trial design

This parallel-arm pragmatic equivalence cluster RCT and process evaluation explored the impacts of patient-centred monitoring by comparing the ADRe screening and management system for potential ADRs [8,46] with usual care. The allocation ratio of clusters was 1:1. The study was conducted to accord with the Consolidated Standards of Reporting Trials (CONSORT) 2010 statement extension to cluster-randomised trials [53], in conjunction with the Template for Intervention Description and Replication (TIDIER) guidelines [54] (S1, S2 Files). The trial was undertaken between 1^st^ October 2021 and 31^st^ August 2022.

### Settings and participants

Primary care general practices in South West Wales typically operate as groups of cooperating practices in a geographical locality. We approached 24 General Practitioner (GP) groups to explore their willingness to participate in the study, and selected a group that comprised six individual GP practices in one valley, each with several GP partners. We recruited 60 patients from these six primary care practices, where the pharmacist and GPs were willing to review completed ADRe documents. The six practices worked as a group of collaborating doctors and allied professionals. The study area is amongst the most deprived in Wales for income, employment and health; however, it enjoys relatively good transport links. Over half the patients are amongst the 20% most deprived in terms of health, measured as long-term illness, premature death, cancer incidence, recorded long-term and mental health conditions, preterm birth and childhood obesity [55,56].

### Inclusion criteria

Patients were identified by their practice pharmacist. Written consent to participate was obtained by researchers. Eligible patients were aged 65 or older, living in their own homes and prescribed 5 or more regular medicines, long-term (vitamin or nutritional supplements and emollients were not included as they rarely cause ADRs). Where medicines were prescribed as PRN (*pro re nata)*, patients were asked about mode of administration; only medicines taken daily were included.

### Intervention

In addition to usual care, patients in the intervention arm practices completed the ADRe profiles and, 20 weeks later, attended a scheduled ADRe appointment with the nurse researcher in either their GP practice or their own home, as preferred, to administer the profile for a second time and check vital signs. The ADRe Profile compiled information on potential ADRs, ADEs and health status through vital sign measurements, observations and reported symptoms. The patients’ primary care pharmacist reviewed the ADRe Profiles, including prescribed medicines, alongside the decision-support patient management suggestions from the instrument, and addressed problems at their discretion. The pharmacists agreed that no formal training on ADRe was necessary. The second ADRe Profile was compared with the first (detailed in S3 File).

Patients in the control arm received usual care. This included medicines reviews by GP or pharmacist, when triggered by usual clinical processes.

### Outcomes

We compared, at individual level, changes in problems documented in: A) practice medical records (2 occasions per practice between 1^st^ November 2021 and 31^st^ July 2022), and B) the first and second ADRe Profiles to assess clinical gains or harms.

A) The primary outcome was ‘health problems addressed’, as indicated by the medical records. ‘Health problems’ were defined as signs and symptoms listed on ADRe [46] [44 p.5], and S3 File. ‘Problems addressed’ were defined as those where action was taken following problem identification, for example by changes in prescribing or referrals or health promotion [44 p.5] (examples are described under ‘clinical gains reported by patients’ and in S4 File, S1, S2 Tables). In both arms, at baseline (before) and follow-up (after ADRe use in one arm), using medical records, we assessed the number and nature of health problems identified and addressed and changes in prescribing, including deprescribing (see glossary), dose reduction, new prescriptions, laboratory tests, and recording of vital signs [57].
B) In the intervention arm we assessed 1) new problems identified or changes in problems noted and documented on ADRe at first and second administration, 2) problems addressed, 3) clinical significance, 4) time and staff costs of using ADRe, 5) participants’ evaluations, 6) and potential harms. We did not compare vital signs, medicines self-management or health promotion items; these items contributed to case descriptions. The process evaluation explored intervention fidelity and stakeholders’ experiences of ADRe.

### Harms

All changes in symptoms and prescriptions were recorded, reviewed by the primary care pharmacist, referred to the prescriber if needed, and analysed.

### Sample size

The sample size was calculated as described [44] based on standard formulae [58,59] and accounting for clustering.

In an earlier ‘before and after’ single arm intervention study, with patients acting as their own controls, ADRe indicated that 17 of 19 (89%) patients receiving polypharmacy had at least one clinical problem that might require a prescribing change [8]. We hypothesised that 9/19 (47%) would have shown some improvement without intervention. This suggested that our new study required an effective sample of 40 patients (20 in each arm), with 5% significance and 90% power [58]. Based on the reported intra-cluster coefficient (0.02) [43],, and a design effect of 1.18, to achieve an effective sample size of 40, an actual sample size of 48 was needed [60]. Ten patients in each of 6 clusters (GP practices) allows for 20% loss to follow up (S3 File).

### Randomisation

Practices were randomized after enrolment was completed. To stratify the six practices, we ascertained the socio-economic status of the population served, using the Welsh Index of Multiple Deprivation (WIMD) 2019 [61]. WIMD is a composite of eight domains (or types) of deprivation: a) Income b) Employment c) Health d) Education e) Access to Services f) Community Safety g) Physical Environment, and h) Housing. Each domain is compiled from several different indicators, and the domains are combined to give a single rank for the local area [61]. Practices were randomised by an independent, external statistician using random number generating software, so that they were equally assigned to intervention or control arms (S3 File).

Blinding of participants was not possible, due to the nature of the intervention. The data analyst was blinded to trial arm.

### Statistical analysis

Analyses were at the individual patient level [44]. Differences between trial arms in the changes in number and nature of problems identified and addressed and prescribing were compared using bivariate statistics. The impact of trial arm and independent factors and covariates on dichotomised dependent outcomes documented in the medical records (>1 problem addressed, at least 1 new problem recorded, and prescribing adjustments) was assessed using multilevel generalised linear mixed models (GLMM), accounting for variability across GP practices by including practice as a random intercept [62]. We adjusted for age, deprivation and comorbidity. Data on age and deprivation were dichotomised: patients aged 65–75 (reference group) were compared with older patients; those in the most deprived WIMD quintile (reference group) were compared all with other patients, reflecting the study population. Comorbidity at baseline was summarised by ‘Number of long-term conditions’, entered as a continuous variable.

Changes in reported symptoms on the ADRe Profile between first and second administration in the intervention arm were enumerated. Clinical significance, staff costs of ADRe implementation and process outcomes were described. The ‘modified intention to treat principle’ [63] was adopted, adhering to the ‘intention to treat’ principle and excluding missing data. No data were imputed. There were no subgroup analyses.

### Process evaluation

Alongside the RCT, a process investigation examined participants’ perspectives on ADRe. A questionnaire with free-form comments was administered to 26 of 27 service users in the intervention arm. Semi-structured interviews with two pharmacists working with this ADRe project [52] supplemented the data. Fieldnotes documented researcher interactions with professionals and service users.

### Ethics

Ethical approval was granted by the Health Research Authority Research Ethics Committee on 17th March 2021 (Integrated Research Application System [IRAS] 292693). All participants had full capacity. They gave written consent for participation, *verbatim* reproduction of quotations, and for researchers to access individual-level patient data.

## Results

### Participant flow and recruitment

**Fig 1:**
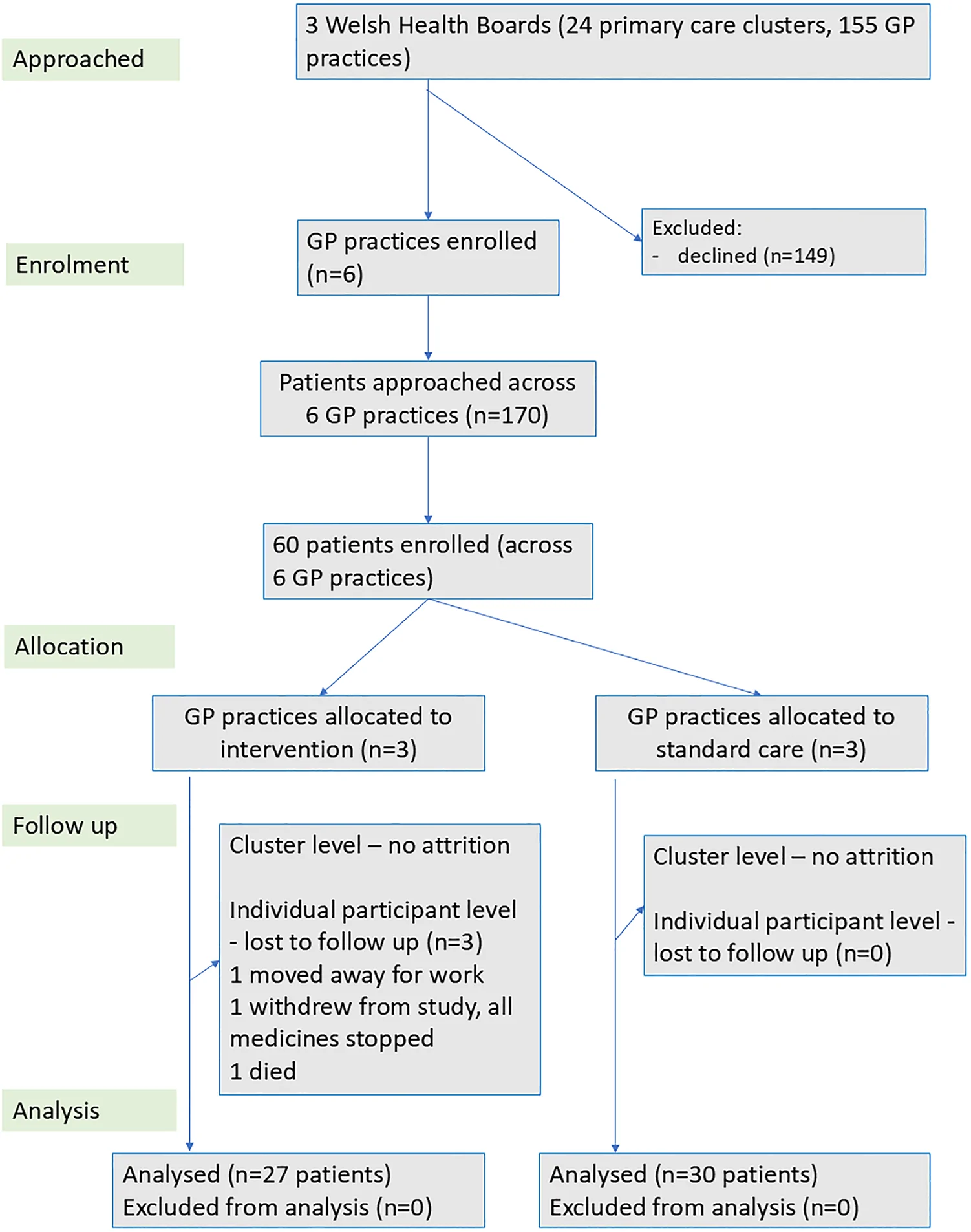
Trial participant flow diagram.

We recruited one of the 24 GP Practice groups approached. These groups are locally determined arrangements of GP practices. From the six practices in the participating group, we enrolled 60 patients (35.3% of 170 approached). No practices withdrew. Three intervention arm patients were lost to the study: two before administration of the intervention, due to death and moving out of area. The third withdrew after first administration of ADRe, stating that all medicines had been discontinued by secondary care teams. All three were excluded from analyses (Fig 1). Protocol deviations are detailed in S3 File; most notably, time between the data collection rounds was more protracted than planned for 22 of 27 patients. There was no cross-over between trial arms. No patients moved house; therefore, there were no changes in WIMD quintiles. Data collection from medical notes was complete. Missing data arose in the ADRe questionnaire when: a) patients completed ADRe by telephone or were unable to stand it was impossible to obtain measurements of all vital signs (5 of 27 respondents); b) one question was designated as ‘discretionary’ due to cultural sensitivities: the question on sexual dysfunction was omitted by 15 of 27 respondents; all other questions were complete. These data were not subjected to inferential analyses. (Further details are in S3 File.) Differences between those responding and not responding militated against transferability of responses, and hence data imputation.

### Baseline characteristics

At baseline, the control and intervention arms were comparable in terms of numbers of prescribed medicines, long-term conditions, and problems noted in medical records (Tables 1 and 2).

**Table 1:** Patient characteristics at baseline.

	Intervention arm, n = 27		Control arm, n = 30	
	Mean [SD]	Range (min-max)	Mean [SD]	Range (min-max)
Age*	73.85 [5.69]	24 (66-89)	77.17 [6.29]	25 (68-93)
Number of long-term health conditions per patient	3.78 [1.37]	5 (2-7)	3.67 [1.49]	6 (1-7)
	Number (%)		Number (%)	
Sex (m/f) female n (%)	9 (33.3%)		18 (60%)	
**Deprivation: most deprived quintile WIMD	16 (59%)		23 (77%)	
***Age 65–75	19 (70%)		15 (50%)	

*Shapiro-Wilk test for data distribution: intervention arm P 0.01; control arm P 0.01.

**There were 0–4 patients in each of the other four quintiles.

***Dichotomisation of age was based on definitions of premature death, used in UK statistics [64].

**Table 2:** Long-term conditions recorded.

Clinical conditions in medical records	Intervention arm (n = 27) number (%)	Control arm (n = 30) number (%)
Hypertension	18 (67)	17 (57)
Chronic pain	8 (30)	11 (37)
COPD	10 (37)	10 (33)
Osteoarthritis	5 (19)	10 (33)
Diabetes	6 (22)	9 (30)
Mental health conditions	8 (30)	9 (30)
CHD	7 (26)	8 (27)
Thyroid disorders	6 (22)	6 (20)
Atrial fibrillation	<5	5 (17)
Stroke	0	<5
Rheumatoid arthritis	0	<5
Gout	5 (19)	0
Other*	<5	10

Notes to table. COPD chronic obstructive pulmonary disease, CHD coronary heart disease.

Numbers 1–4 are reported as <5 to ensure confidentiality. Most patients had > 1 condition.

* Including chronic kidney disease, respiratory problems, cancer, renal problems, prostatic enlargement, epilepsy, gastrointestinal problems.

## Outcomes

### A. Comparing intervention and control arms

#### Problems found and addressed.

The number of problems identified in the medical records increased in both arms. The difference between arms was statistically significant after the intervention, at follow-up, suggesting that the intervention had increased recognition of problems sufficiently serious to be documented in the medical records. Significantly more problems were addressed in the intervention arm. Before the intervention, there was no statistically significant difference between the trial arms in the mean number of medicines prescribed. There was a statistically significant difference between the two arms in de-prescribing. The number of medicines prescribed increased in the control arm. In the intervention arm, increases were balanced by discontinuations (Table 3).

**Table 3:** Trial arm differences in problems identified, problems addressed and prescribing, as documented in medical records.

	Intervention arm, n = 27		Control arm, n = 30				
Observations Numbers of:	Mean [SD]	Range (min-max)	Mean [SD]	Range (min-max)	t(df)	P	Cohen’s d (95%CI)
Problems identified at baseline*	1.15 [0.86]	3 (0-3)	0.87 [1.20]	3 (0-3)	1.01 (55)	0.16	0.27 (−0.26-0.79)
Problems identified at follow-up *	3.59 [2.06]	9 (0-9)	1.77 [1.17]	5 (0-5)	4.17 (55)	<0.001	1.11*** (0.54-1.67)
Number of problems addressed**	2.48 [1.45]	6 (0-6)	1.40 [1.13]	4 (0-4)	3.15 (55)	0.001	0.84*** (0.29-1.38)
Medicines prescribed at baseline*	7.74[2.46]	10 (5-15)	8.37 [2.59]	10 (5-15)	−0.93 (55)	0.18	−0.25 (−0.77- 0.28)
Medicines prescribed at follow-up*	7.89 [2.90]	12 (5-17)	9.07 [3.04]	11 (5-16)	−1.49(55)	0.07	−0.40 (−0.92-0.13)
Deprescribing	0.96 (0.90)	3 (0-3)	0.53 (0.97)	4 (0-4)	1.98 (55)	0.03	0.53** (−0.01-1.05)

Notes to Table 3. SD Standard deviation

* Baseline data were extracted before the intervention was delivered. Follow-up data were extracted after the intervention was delivered in the intervention arm. Control arm participants received usual care throughout; they did not receive the intervention.

** Investigated or resolved

The effect size was calculated by dividing the mean difference by the standard deviation [65]. A Cohen's d of 0.2 is considered a small effect, 0.5 a medium effect**, and 0.8 a large effect***.

There were no missing data in this table.

### Recording vital signs

From the medical records, documentation of vital signs and laboratory tests increased more in the intervention than the control arm (Table 4). Two patients in the control arm and one in the intervention arm had had no vital signs documented for at least 4 years. There were no recordings of postural measurements, but 3 patients reporting falls had postural hypotension and antihypertensive prescriptions (patients 7, 13, 16).

**Table 4:** Differences in vital signs and laboratory tests, as documented in medical records.

Parameters in the medical records	Intervention arm (n = 27)	Control arm (n = 30)	OR (95%CI)
Any vital signs/measurements recorded in the year before intervention	16 (59.3%)	20 (66.7%)	0.73 (0.25-2.14)
Any vital signs/measurements recorded at follow-up	22 (81.5%)	21 (70.00%)	1.89 (0.54-6.56)
Laboratory tests in the year before intervention	18 (66.7%)	17 (56.7%)	1.53 (0.52-4.50)
Laboratory tests at follow up	25 (92.6%)	18 (60%)	8.33 (1.66-41.90)
Weight recorded before intervention	9 (33.3%)	16 (53.3%)	0.44 (0.15-1.23)
Weight recorded at follow-up	14 (51.9%)	16 (53.3%)	0.94 (0.33-2.67)

Note to Table 4: there were no missing data in this table.

**Fig 2:**
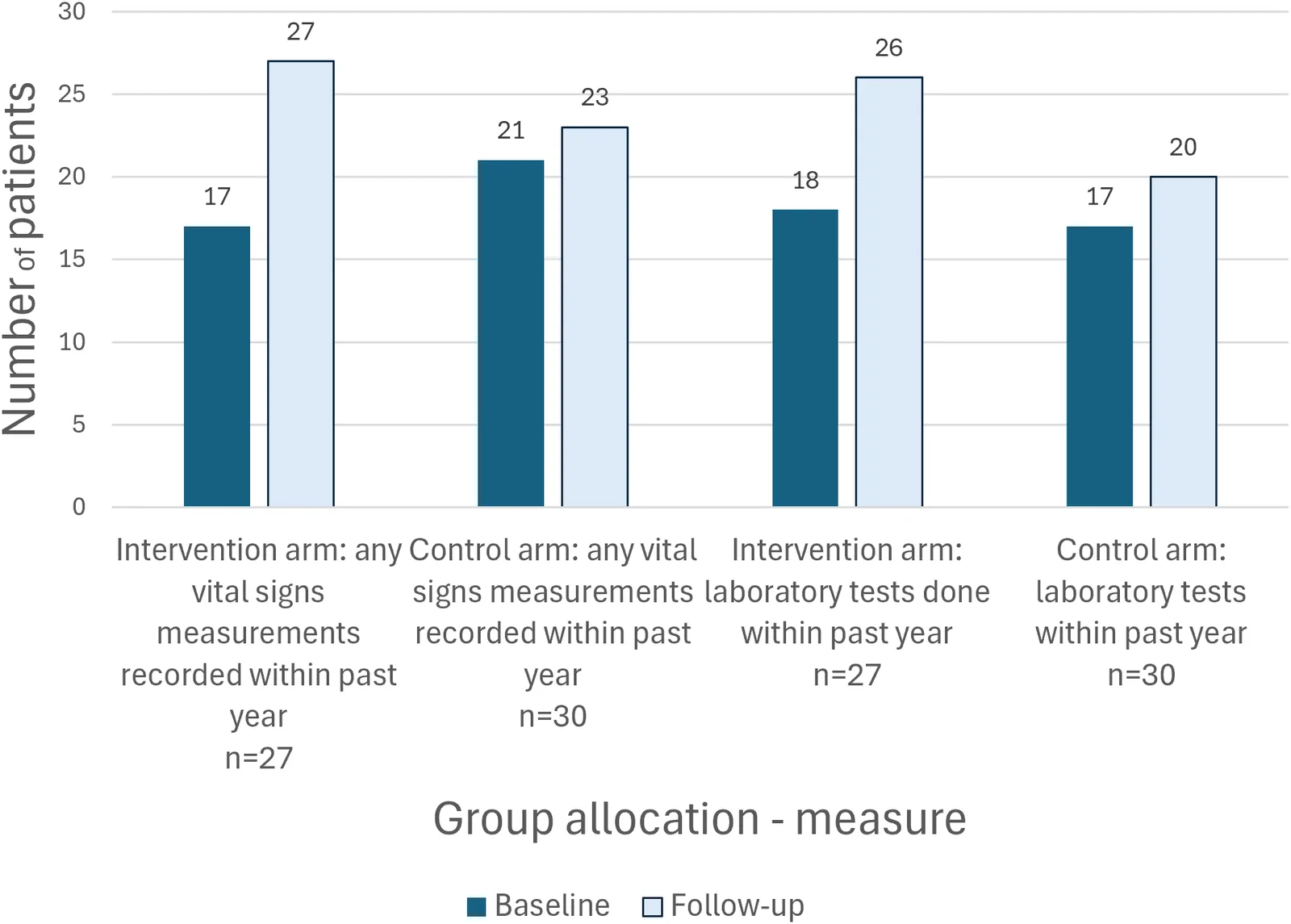
Vital signs and laboratory tests.

For 11 patients at baseline and four at follow-up, vital signs were documented on ADRe, but not in the medical records. The recording of weight and laboratory tests increased in the intervention arm, but not the control arm (Fig 2).

### Problems addressed – adjusted analyses

Intervention arm patients were more likely to have 2 or more problems addressed, when age, deprivation and number of long-term conditions were accounted (aOR 7.48, 1.99-28.10) (Table 5). Fewer intervention arm patients had new problems documented in their medical records. The difference was modest (19 of 27, 70% vs 23 of 30, 77%) and statistically non-significant. Recording of new problems was associated with socio-economic status: 10 of 18 (56%) patients in the 4 most affluent quintiles had a new problem recorded, compared with 32 of 39 (82%) in the most deprived quintile (aOR 0.15, 0.03-0.72).

**Table 5:** Changes between baseline and follow-up, as documented in medical records.

	Intervention arm (n = 27) n (%)	Control arm (n = 30) n (%)	OR (95% CI) unadjusted	Trial arm * aOR (95% CI)	Other significant covariates aOR (95% CI)	Overall model significance F(df) P	Pseudo R^2^ conditional
Problems documented in medical records							
>1 problem addressed	22 (82)	11 (36.7)	7.60 (2.24-25.81)	7.48 (1.99-28.10)	None, only trial arm	2.76(4,52) 0.04	0.51
At least 1 new problem recorded	19 (70)	23 (77)	0.72 (0.22-2.36)	1.41 (0.21-9.32)	WIMD, 0.15 (0.03-0.72) **	1.70 (4,52) 0.16	0.45
Prescribing							
Deprescribing, medicines discontinued	15 (56)	9 (30)	2.92 (0.98, 8.67)	2.37 (0.70-8.07	WIMD***, 4.53 (1.19-17.35)	2.08 (4,52) 0.10	0.50
Deprescribing, medicines stopped or doses reduced †	18 (64)	11 (37)	3.46 (1.16-10.29)	2.83 (0.70-11.44)	None, trial arm strongest association	1.54 (4,52) 0.21	0.53
Any new prescriptions	16 (59)	23 (77)	0.44 (0.14-1.39)	0.39 (0.11-1.43)	None, trial arm strongest association	1.39 (4,52) 0.25	0.45

*Notes to table. OR odds ratio, aOR adjusted odds ratio, CI confidence intervals, WIMD Welsh Index of Multiple Deprivation. ICC intraclass correlation coefficients ranged 0.12-0.48. ICC (conditional) for primary outcome 0.36. Full models are available in* S4 File, *S3 Table and* Appendix to S5A-S5E Tables: Parameters of Regression Models. * Generalised Linear Mixed Models (GLMM) with GP practice as the random intercept and adjusted for deprivation (most deprived WIMD quintile vs quintiles 2–5), age (<76), and number of long-term conditions. The count of long-term conditions was normally distributed. Probability distribution was binomial, and link function logit. There are no missing data in these analyses. Conditional pseudo R^*2*^ *is reported, to account for fixed and random effects.*

** Wealthier people had fewer new problems.

*** Wealthier people had more medicines discontinued.

† This outcome measured both discontinued medicines (as above) plus dose reductions.

#### Differences in prescribing.

More deprescribing occurred in the intervention arm. In the intervention arm 15 of 27 (56%) patients had one or more medicines withdrawn, compared with 9 of 30 (30%) in the control arm, OR 2.92, 0.98-8.67. In adjusted analysis, relative affluence was a stronger predictor of deprescribing than trial arm: deprescribing was more frequent amongst more affluent patients (12 of 18, 67% *vs* 12 of 39, 31%, aOR 4.53, 1.19-17.35). When dose reductions were included in de-prescribing, differences between trial arms became statistically significant (OR 3.46, 1.16-10.29); adjusting for age, deprivation and long-term conditions left trial arm as the strongest predictor but confidence intervals were too wide to indicate statistical significance (aOR 2.83, 0.70-11.44). Fewer patients had new prescriptions in the intervention arm, 16 of 27 (59%) vs 23 of 30 (77%) in the control arm, (aOR 0.44, 0.11-1.44). The difference was not statistically significant (Table 5).

### B. Changes between first and second ADRe administration

#### Problems found by ADRe.

Not all problems filtered through the practice processes to be documented in medical records. At the start of the study, there were 31 problems listed in the intervention arm and 26 in the control arm (mean 1.15 per patient [SD 0.86] and 0.87 per patient [SD 1.20], respectively) (Table 3). The real number of problems was higher. When first used, ADRe documentation indicated that of the total of 388 identified problems (mean 14.37 per patient [SD 7.53]); 357 were not documented in the medical records. These problems largely remained uninvestigated.

Excluding ‘vision problems’, which indicated need for glasses in 22 of 27 patients, the most prevalent problems recorded on ADRe were: ‘shortness of breath’ (17 of 27 patients), ‘pain’ (24 of 27), ‘low energy’ (14 of 27), ‘feeling cold’ or ‘hearing problems’ (13 of 27), ‘dry mouth’ (12 of 27) and ‘tingling, pins and needles in fingers’ (11 of 27), ‘dizziness’ (9 of 27), ‘problems with balance’ (10 of 27) and falls within last month (8 of 27).

ADRe detected extrapyramidal symptom (EPS) patterns (tremor, posture and movement problems) in 11 of 27 patients. One of these potential cases of EPS was already recorded by the practice. Hypothyroidism patterns were identified in 6 of 27 (3 were already recorded) and signs of a congestive heart failure were identified in 9 patients (4 were already recorded).

#### Problems addressed through ADRe.

**Fig 3:**
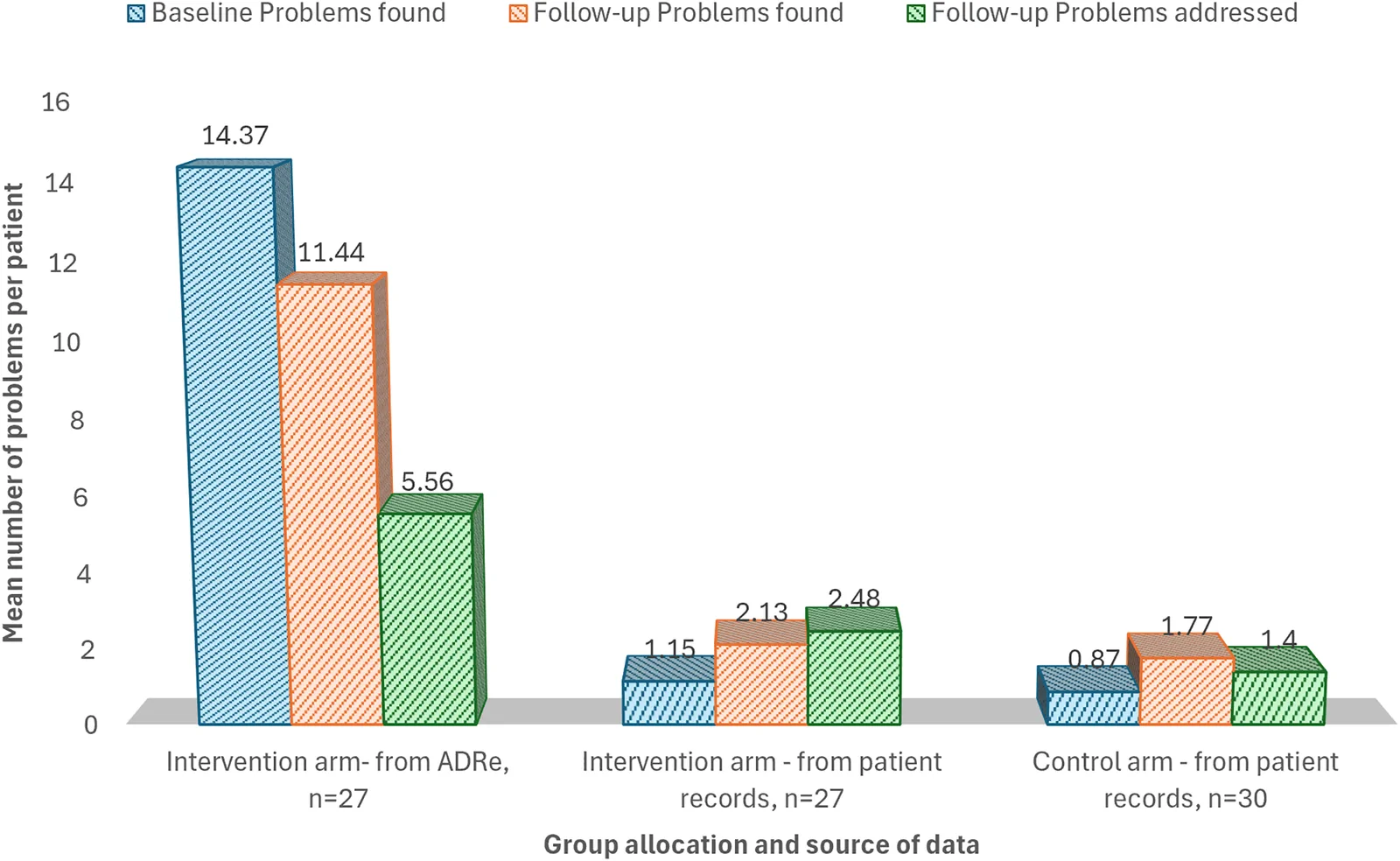
Problems and actions documented in the medical records, baseline and follow-up.

In the intervention arm, the pharmacist acted on 150 of the 388 ADRe-reported problems identified (mean per patient 5.56, [SD = 3.75]), often by escalating the problem for GP review (78 problems in 23 patients). Many (67, mean 2.48 per patient, [SD = 1.45]) problems and actions filtered through to the practice records by the time of the second data collection (round 2) (Table 3). This resulted in more problems recorded as addressed in the intervention arm than in the control arm (Fig 3).

Fewer problems were identified when the ADRe Profile was readministered (round 2): mean 11.44 problems per patient (SD 6.66) compared with 14.37 (SD 7.53) in round 1. The mean reduction of 2.93 problems per patient (SD 2.96), t(26) 5.14, p < .001 was statistically significant, with a small effect size (Cohen’s d 0.10). Every patient had at least one problem resolved, with some reporting up to six fewer problems than present in round 1. Twenty of the 27 patients had a substantive clinical gain. For example, patient 24 had decreased symptoms of fatigue following gabapentin and chlorphenamine deprescribing, and patient 16’s sleep problems resolved following duloxetine deprescribing. No new problems emerged in these patients. Additionally, the academic team identified further issues; 2–21 considerations per patient (mean 7.22, SD = 5.09). For example, patient 608 was hypoxic, falling and showing signs of EPS and cognitive decline; although reduction of gabapentin and morphine offered potential for clinical gain, the patient declined any changes to prescriptions (S4 File, S1 Table).

### Clinical gains reported by patients

The prevalence of 37 problems fell (Table 6, S4 File, S2 Table). Pain was reported by 24 of 27 (89%) patients at round 1, and 16 of 27 (59%) at round 2: attention to analgesia and oedema were instrumental. The reduction in the prevalence of hallucinations was probably due to deprescribing of opioids, antidepressants, beta agonists, antihypertensives or flecainide. Fewer patients reported tremors, abnormal movements, abnormal gait, balance problems, feeling cold, rashes, oedema, excessive sweating, injection site problems, anxiety, panic attacks, fatigue, dizziness, headaches, insomnia, paraesthesia, hearing difficulties, dry eyes, incontinence, chest pain, dyspnoea, swallowing difficulty, diarrhoea, and constipation. Seven problems—cognitive decline, bleeding/bruising, aggression, mood fluctuations, vision problems, dry mouth, and indigestion—were reported by more patients at round 2.

**Table 6:** Changes in problems between first and second ADRe data collection and actions: examples. Intervention arm patients, n = 27 S4 File, S2 Table has full data.

Problem	Round 1 number reporting	Number improving	Number worsening	Round 2 number reporting	Actions contributing to a decrease in symptoms experienced with patient IDs
Abnormal movements at rest	5	4	1	2	14 – none (gabapentin a likely cause) 19 – Sukkarto® switched to metformin (possibility of correcting hypoglycaemia causing movement disorders) 26 – none 27 – mycophenolate dose corrected, reducing risk of tremor and abnormal movements.
Feel cold	13	5	1	9	3 – plan to review beta-blocker 5 – diuretic prescribed (addressed heart failure, and improved circulation), flecainide discontinued (oedema reduced). 7 – dapagliflozin prescribed (treating heart failure) 20 – pain reduced 23 – doxazosin de-prescribing
Hallucinations/ vivid dreams	8	6	0	2	2 – none evident 5 – flecainide discontinued, improving heart failure, improving dreams (Horie et al., 2021) 7 – opioid deprescribed 16 – duloxetine de-prescribing 20 – salbutamol withdrawal 23 – alpha blocker discontinued (less hypotension may stop nightmares)
Sleep problems	9	6	1	4	2 – none evident 6 – pain relief or deprescribing metformin, reducing diarrhoea / gastro-intestinal upsets 13 – opioids deprescribed, emollient prescribed for itching 14 – mirtazapine prescribed (sedative) 16 – duloxetine de-prescribing 23 – de-prescribing of alpha-blocker
Falls in past month	8	4	0	4	11 – none 14 – attended physiotherapy sessions 16 – duloxetine de-prescribing 19 – home visit, advice given with vital signs measurement
Pain	24	9	1	16	1 – amlodipine discontinued (likely leading to reduced swelling and indigestion) 4 – carbamazepine deprescribed (less headache & neuropathy) 5 – new cancer treatment, flecainide stopped (was causing oedema & pain) 6 – Oromorph® and co-codamol prescribed 9 – GP review, advice given 15 – none 17 – Zapain® prescribed 18 – none 19 – cream and ointment prescribed
Hearing problems	13	4	1	10	3 – ear wax removed 8 – none 10 – ear wax removed 19 – none

*Notes to table. There were no missing data for any of these problems.*

The pharmacist acted every time certain problems were noted: bleeding/bruising (9/ 9 patients), behavioural issues (once), and injection site problems (once). The typical response to bleeding was to check clotting parameters and other laboratory results. Other issues were less consistently managed: pain was investigated in 14 of 24 (58%) instances and feeling cold in 8 of 13 (62%), usually by seeking review of analgesia or beta blockers. Urination problems were addressed in 6 of 7 (86%) patients, typically by referring to the incontinence service or recommending pelvic floor exercises, while oedema was addressed in 7 of 10 (70%) patients, typically by seeking GP review of possible heart failure. This suggests variability in response depending on the problem and its perceived severity. For example, patient 5 was prescribed a complex regimen of cardiovascular medicines, including warfarin, flecainide, and bisoprolol. ADRe review identified several problems, such as irregular heart rate, bleeding and easy bruising, leg oedema, cold sores, vivid nightmares, sleep disturbances, and pain. Following ADRe escalation to the pharmacist and GP, laboratory tests and vital signs were updated, and a GP review was initiated. The dose of warfarin was reassessed, and flecainide was deprescribed. New medicines were added: Prostap® injections (leuprorelin) and bumetanide. The additional care resulted in: resolved pain, diagnosis and treatment of early-stage prostatic cancer, and cessation of vivid, nightmarish dreams. This case demonstrates how ADRe review and collaborative approaches improved symptom control, early diagnosis of a serious condition, and enhanced quality of life. (Illustrative cases are in S4 File, S1 Table.)

### Costs

ADRe Profile completion required a mean of 30 (SD 13) minutes of nursing time, costing ~ £23 GBP. In addition, the primary care pharmacist spent approximately 15–20 minutes per review, costing £14. As no routine medicines reviews were scheduled, the pharmacist requested GP consultations for 23 of 27 patients, incurring an additional cost of £32 per appointment. Patient self-completion of the ADRe Profile substantially reduced staff input, limiting the nursing role to recording vital signs delegated to a nursing assistant, which costs approximately £4 for 5–10 minutes’ work. Total staff cost per patient-completed ADRe Profile ranged from £18 (without GP involvement) to £50 (with GP consultation). This would rise to £73 if 20–30 minutes of nursing time were required for patients unable to self-complete ADRe.

### Process evaluation

From earlier research in residential homes and access negotiations, the research team was aware that clinicians had doubts about adopting ADRe because of the time they believed it would take against the background of already high workloads. This was reflected in the unwillingness of GPs to complete planned qualitative interviews, and is probably the most challenging barrier to implementation of ADRe in general practice.

Service user perspectives were more positive. High adherence to the research protocol was observed, ensuring completion of ADRe items. A theme emerging from service users’ comments was that talk in GP consultations was not generally an effective way of identifying negative effects of polypharmacy. Participants reported a pattern of selective disclosure of health concerns to their GPs, reporting only issues they deemed ‘valid’ and ‘serious enough,’ due to the perceived burden mentioning minor issues would impose:

*‘People, especially elderly, do not want to bother the doctor.’* (patient 12, taking pregabalin, morphine, an antidepressant and 3 other medicines, with EPS, oedema and skin breakdown).

In commenting on the usefulness of ADRe, many service users suggested that systematic monitoring of possible ADRs would help overcome difficulties in talking to GPs about their worries. For example, patient 2 (taking 3 antihypertensives, with postural hypotension, balance problems and a history of falls) felt that ‘*GPs are very difficult to contact*’, while patient 11 (taking 4 antihypertensives, reporting dizziness, and with a history of falls) said: *‘It is difficult to speak to them (GPs), they are not available.’* A sense of disinterest from primary care staff also emerged as a significant barrier. Patient 21, (taking an NSAID for pain from arthritis, hypertension recognised) reported *‘the worst thing is when they (health professionals at the GP surgery) do not care,*’ and patient 9, (prescribed morphine and inhaled beta agonists with steroid for COPD and dyspnoeic) explained: *‘Sometimes the surgery makes me feel like I am wasting their time.‘*

Participants described ADRe as a welcome alternative to discussion of problems in face-to-face consultations. Patient 7 (taking 3 medicines lowering BP, with postural hypotension, arrhythmia, falls and pain) explained ‘*this is a better system than seeing the GP, I find that GPs are reluctant to make any changes, they follow what the hospital prescribed and are reluctant to stop any medicines. Also talking to GPs is very rigid and formal, you got more out of me than a GP would.’* Similarly, patient 12 described how the approach facilitated by ADRe felt more accessible: ‘*With this kind of set-up, (it is) much friendlier, you get more out of people’.*

Some participants suggested that the value of ADRe was that it created a dynamic of collaboration. Patient 21 said: ‘*It is a 3-cornered arrangement which works’,* and contrasted this with the negativity of ‘*feeling that the surgery don’t [SIC] care’*. According to patient 4, ADRe brought *‘1st class benefits’,* saying*: ‘There has been COVID for three years and a wall has emerged between patients and doctors. You chipped away at that wall’.* Patient 27 welcomed the opportunity for meaningful dialogue: ‘*I can ask you questions, because I haven’t been able to see anyone, I had no-one to talk to about my problems, that is what is missing.’* These comments illustrate how the ADRe Profile may help bridge communication gaps and uncover conditions that do not surface in routine consultations, and this might be expected to improve outcomes if this version of ADRe can be implemented more widely.

Both pharmacists interviewed also saw benefits in terms of early detection of clinical issues that might otherwise remain unaddressed and lead to clinical deterioration and admission. They saw particular value in the cues that ADRe offered to link condition-specific signs and symptoms to possible ADRs. Pharmacist 1 reflected on how the tool prompted consideration of underlying conditions in patients presenting with seemingly non-specific symptoms: *‘…lots of patients that had put ‘tired all the time’ or ‘bruising’ or ‘bleeding’, it [ADRe] just kind of prompted you to check for thyroid or anaemia, so it kind of prompted you to check for bloods as well (. …) An issue that isn’t important for the patient could be important for the prescriber because they’ve got the knowledge to pick up on something.’* Pharmacist 1 mentioned the utility of ADRe in broadening the clinical perspective: *‘...at least there were more things to consider for the patient’.* They suggested that ADRe played a mediating role in interdisciplinary communication, saying that it ‘*really helped me bridge that gap between the patient and the doctor*‘.

However, both pharmacists shared the concerns about the extra burden of work associated with using ADRe that researchers had heard expressed by doctors and nurses. Pharmacist 2 said that they ‘*would be worried about how much work it might create. (...) If they are on a lot of meds, to go and check every interaction would be very, very time-consuming*‘. These findings reflect a tension between the clinical benefits of ADRe and the pragmatic challenges of utilising review tools of this kind in GP practices and pharmacies affected by resource constraints.

### Harms

No attributable disbenefits and harms were observed.

## Discussion

When the ADRe Profile was added to usual care of older people, some previously unidentified problems were addressed. The provenance of many problems was uncertain, but all were important to patients. For 2 of 27 patients (IDs 5, 6), ADRe identified life-changing problems, and triggered timely management (flecainide deprescribing, and vitamin B12 deficiency causing asthenia and paraesthesia in a patient prescribed metformin). Other gains were more modest, e.g., relief of itching following prescription for an emollient (ID 9), and some symptoms abated for no reason apparent to researchers, e.g., insomnia and vivid dreams subsided (ID 2). All patients in the intervention arm had actual or potential clinical gain. For 9 of 27, this was relief of pain; four people were no longer falling. ADRe influenced prescribing, prompting adjustments and deprescribing. Demographics, such as deprivation, also influenced medicines management.

### “Bridge that gap”: patient to prescriber communication

The prevalence of problems identified here is higher than the estimated 20–30% prevalence of ADRs [17] or ADEs in primary care [66]. ADRe’s monitoring found unaddressed problems, some more serious than others, in all patients, in line with community pharmacists’ reports of drug-related problems in >80% patients, (in Germany) [67], and >70% in primary care institutions [68].

### Comparison with other instruments

Structured, standardised patient questioning improves ADR reporting [69], but, outside clinical trials, is rarely incorporated into practice. Most guidelines and ADR instruments focus on single conditions, but patients with multi-morbidity have the greatest needs [10, p.8]. The NHS provides quality standards for single conditions, but these are not monitored [70, p.27], in part because patients rarely have a single problem.

Checklists devised to record the adverse effects of medicines are mostly brief, without decision support and untested in RCTs [70]. In contrast, six instruments tested in RCTs designed to ameliorate ADRs for poly-medicated adults in primary care [41] increased ADR reporting, and three, like ADRe, improved clinical outcomes overall [71–73]. All three were administered by GPs or pharmacists, and the additional patient contact time needed might be too expensive for NHS GPs. ADRe’s costs are minimised by patient self-completion [74].

Checking prescribed medicines against lists of high-risk medicines and interactions without questioning patients may not take full account of patients’ current clinical situations. Decision tools predicting future harm from medicines, such as STOPP-START [75], require more clinical information than is available from medicines charts and laboratory reports [76]. ADRe bridged this information gap, bringing individualised, structured reports of patients’ ongoing problems to pharmacists reviewing medicines records. Through both process (Table 4) and clinical changes (Table 6, S4 File, S1, S2 Tables). ADRe complemented pharmacist reviews – it did not replace them.

### What matters to patients

There is no consensus around selection of trial outcomes in RCTs of complex interventions for poly-medicated patients. Systematic reviews of interventions to alleviate ADRs have focused on easily measured outcomes, such as prescribing, mortality, hospitalization, falls or quality of life scales, without demonstrating patient benefit in primary care [30–34,36–40]. In this trial, focusing on patients’ actual, not predicted, problems led to patient-reported clinical gain, over and above improved prescribing.

Problems documented in medical records predominantly involved patient-reported acute issues and routine checks associated with diagnosed single medical conditions [77]. Participants’ perceptions of professionals’ workload led to them selectively reporting health problems judged to be ‘valid’ and ‘serious enough.’ This under-reporting of common but impactful symptoms, such as pain, balance, EPS, fatigue, and dyspnoea, created disparities between problems identified through ADRe and the medical records. Patients’ hesitancy to report symptoms was also due to concerns regarding ageism, burdening professionals and accessibility [78,79], plus a tendency to accept symptoms as normal signs of aging [79], including Parkinsonism possibly caused by gabapentinoids. The ADRe Profile shifted the judgment of problem validity from service users to professionals, and facilitated identification of long-standing, unnoticed problems, some of which were very serious [8,43,48–51].

Professionals’ and patients’ views on the importance of symptoms [80,81] and their aetiology often differ [82]. All patients were grateful for the support or “first class benefits” offered by ADRe, as in other settings [8,43,45,50], but doctors [74] were concerned about medicolegal vulnerability [83], and more doubtful about ADRe [52].

Patients highlighted that seemingly minor previously unreported health issues impacted their lives, and warranted early identification. The additional information provided by ADRe enhanced management and improved outcomes, often through recognition of symptom patterns (e.g., heart failure in patients 1 and 23). The significant reduction in the number of patients in pain, reduced reporting of tremor, dyspnoea or urination problems, and early diagnoses of prostatic cancer and vitamin B12 deficiency, demonstrate the tangible impact of ADRe. ADRe often generated multiple small, incremental improvements, accruing “marginal gains” [84], making important differences overall.

### Costs

ADRe addressed many problems of minimal cost to the NHS, for example, pain, dyspnoea, confusion. Patients had not ‘troubled’ their doctors with these problems. Any economic benefits of ADRe would probably accrue from pre-empting escalation and hospitalization. Many issues ADRe identified were resolved by pharmacists [8,45,50]. Most GP consultations initiated by the trial were due to unmet need recognised by ADRe, rather than new costs.

In general practice, there may be no routinely scheduled medicines reviews, and nurses’ and pharmacists’ time would entail new costs of £18 to £73 per patient. These would be offset by the reduction in direct costs of experiencing ADEs, estimated at ~$200 (£157) per 30 days per patient [85]. Quarterly administration of ADRe costs £100–300 a year, less than the estimated direct costs of living with ADEs (£2447 each year) [85].

In the ADRe studies, some 10% patients had a serious ADR, that would have led to admission had not problems been identified [8,43,50,51,86]. The cost of each preventable admission due to ADRs or ADEs has been estimated as between £5,000 GBP [87,88] and above £8,000 [13]. Each falls-related admission costs ~ £18k [89] plus costs of hospital bed occupancy. This suggests that ADRe’s prompts for falls risk assessments, vision checks, reduction of sedatives, antidepressants or antipsychotics could generate savings.

Incorporating ADRe into proposals for annual appraisals and medicines reviews [90], would be unlikely to add to their costs, assuming that patients self-completed ADRe, and vital signs measurements were integral to reviews. Currently, primary care services are not mandated to routinely record vital signs: postural hypotension was never recorded (Table 4). Since systematic reviews suggest current initiatives do not improve clinical outcomes [30–34,36–39], and there is no established strategy to monitor patients for the effects of their repeat prescriptions [41,91], larger trials are needed to examine the impact of ADRe on hospitalization, and associated costs.

### Prompts, checks and prevention

WHO mortality data indicate that preventing avoidable medicine-related harm is critical [11]. Failure to monitor patients for ADRs and ADEs causes many preventable hospitalizations [12,29,92]: the problem is long-standing [1–4], and worsening [5–7].

Medicines optimisation underpins patient safety. Both the General Medical Council’s [93], and the Nursing and Midwifery Council’s [94] professional standards include monitoring patients for the effects of prescribed medicines, particularly before repeat prescriptions are issued, but do not specify how this might be done. ADRe fills this gap. ADRe was effective in the present study: it triggered reviews for all patients and, for 2 of 27, life-saving interventions. Some problems warranted new prescriptions (for example, for pain or anaemia), whilst others, such as xerostomia (n = 5) or oedema (n = 7) or itching (n = 5), were ameliorated by using information on ADRe. Signposting emerged as a crucial benefit, directing patients to relevant healthcare services or non-prescription preparations [95].

Preventive health care is universally advocated [96,97], but there are no effective risk-prediction models for ADRs [98]. A comprehensive tool, such as ADRe, shared between clinicians would address patients’ concerns around feeling ‘treated in silos’ and suboptimal multidisciplinary working [99, p.17].

## Strengths and limitations

Randomisation minimised bias and permitted causal inference. Baseline characteristics were well-balanced. Patient recruitment rate was good (60 of 170 approached). There was no unaccounted attrition. A single pharmacist implemented ADRe, ensuring consistency, but risking contamination between arms [100].

This was a small RCT in one economically deprived valley in South-West Wales. The sample was too small to explore hospitalization or mortality. Corollaries of the small sample size were low counts in some categories, necessitating simplification of variables, and restriction in the number of covariates. The covariates selected reflect the important considerations in our communities. The diverse domains of the WIMD obviate the necessity of considering each separately in small studies. The need to narrow confidence intervals and reduce the risk of type 1 errors by introducing further covariates was balanced against the risk of over-fitting, which would limit the validity of findings [101]. We acknowledge that our exploratory models, based on 57 patients, are vulnerable to over-interpretation and inclusion of secondary outcomes, without adjusting for multiple comparisons, increases the likelihood of reporting misleadingly positive results (type 1 errors). However, statistical findings should be considered alongside the narrative accounts of patient benefit when determining the practical adequacy of ADRe [102].

The restricted trial setting limits generalisation of findings to more affluent areas where the doctor: patient ratio is higher [103], and the prevalence of polypharmacy lower [27]. The practices recruited may have been unusually open to change [52]. Transferability of evidence-based practice and trial findings from affluent to deprived communities or from urban to rural settings and *vice versa* should not be assumed without further research in the target setting [104]).

Blinding was not feasible because participants, practitioners and researchers were aware of the intervention, potentially introducing biases, such as the experimenter [105], Hawthorne [106] or John Henry effects [107]. Self-reported outcomes are vulnerable to social desirability bias, and subjective interpretation of symptoms [108], but there is no reason to assume this would change between data collection rounds.

The provenance of problems identified was not explored with de-challenge and re-challenge, the ‘gold standard’ for ADR detection. This study was not designed to meet the criteria for ADR signal detection [109]. Formal ADR assessment scales have low inter-rater reliability [110,111], and were not deployed.

Symptom aetiology was often uncertain or mixed (for example, patient 3 was prescribed 40 mg furosemide, and hearing improved when ear wax was removed); however, there was a statistically significant reduction in problems following ADRe administration. Many symptoms resolved when acknowledged and treated, congruent with definitions of ADEs as under or over-treatment [85]. Some problems abated when prescribing changed: for example, EPS improved when doses of mycophenolate (patient 27) and gabapentin (patient 24) were reduced. Severity of signs and symptoms was assessed by patients and clinicians, not ADRe; inclusion of trivial symptoms was minimized by using problems in medical records as the primary outcome.

Some patients declined advice to discontinue gabapentinoid or opioid prescriptions, despite EPS (patients 12, 22, 26), reducing ADRe’s potential impact. The study’s timeframe limited exploration of all associations between symptoms and medicines identified in ADRe decision support and the outcomes of referrals to secondary care.

Staff costs were calculated as these reportedly represent a barrier to ADRe implementation [8,50]. The costs of medicines in this study were too low to make a financial impact. We were unable to put a monetary value on reduction in pain, hallucinations or falls.

## Implications

More could be done to reduce the harm associated with ADRs and ADEs [8,41,45,74]. The ADRe Profile with decision support facilitated systematic, comprehensive, person-centred monitoring by structuring communication between patients, pharmacists and prescribers. This addressed some instances of unmonitored avoidable medicine-related harm and supported safer prescribing. The clinical and practical significance of these findings [112,113], and associated statistical significance, do not guarantee adoption and implementation of ADRe [114–117]. The reluctance of primary care practices to engage with ADRe [52], and review of the contextual challenges [118], indicate that further work on implementation of these exploratory findings is needed.

In 2017, the WHO launched the Global Patient Safety Challenge ‘Medication Without Harm’ to develop systems for medicines management that would halve avoidable medicine-related harms by 2022 [9]. However, the prevalence of unmonitored polypharmacy continues to rise [29], along with admissions [5–7] and deaths [11] attributable to ADRs and ADEs. Implementation of WHO plans to reduce iatrogenic harm from polypharmacy, and promote ‘active participation of patients’ [10, p.36], will involve primary care time, but any savings from reduced admissions will accrue to secondary care. Implementation of such altruism on the part of primary care demands holism and leadership.

Taken with previous RCTs [43,47] and observational studies [8,49–52,86], we suggest that ADRe consistently identifies and addresses important problems unrecorded elsewhere, and reduces health-related problems for many patients by facilitating timely intervention. Is ADRe [46] one of the cost-effective prevention strategies the NHS needs [97]?

### Protocol

The trial protocol is published [44].

## Glossary

**Table:** 

			Reference
ADE	Adverse drug event	ADEs I include ADRs, subtherapeutic effects of medication therapy (STE), drug dependence, drug intoxications and untreated indications.	Gyllensten H, Rehnberg C, Jönsson AK, Petzold M, Carlsten A, Andersson Sundell K. Cost of illness of patient-reported adverse drug events: a population-based cross-sectional survey. BMJ Open. 2013 Jun 20;3(6):e002574. https://doi.org/10.1136/bmjopen-2013-002574. PMID: 23794552; PMCID: PMC3686161 p.2
ADR	Adverse drug reaction	ADRs are noxious and unintended responses to a medicinal product	Council for International Organizations of Medical Sciences (2025) Glossary of ICH terms and definitions. Available from: https://cioms.ch/wp-content/uploads/2025/12/CIOMS_ICHglossary_V9_9December2025.pdf accessed 24th April 2026 p.11
	Deprescribing	Deprescribing is the process of withdrawal of an inappropriate medication, supervised by a health care professional with the goal of managing polypharmacy and improving outcomes.	Reeve E, Gnjidic D, Long J, Hilmer S. A systematic review of the emerging deﬁnition of ‘deprescribing’ with network analysis: implications for future research and clinical practice. Br J Clin Pharmacol. 2015 Dec;80(6):1254−68. https://doi.org/10.1111/bcp.12732. PMID: 27006985; PMCID: PMC4693477. P.1266
EPS	Extrapyramidal symptoms	Parkinsonism, with posture and movement disorders and tremor.	Lee J, Muzio MR. Neuroanatomy, Extrapyramidal System [Updated 2022 Nov 9]. In: StatPearls [Internet]. Treasure Island (FL): StatPearls Publishing; 2025 Jan. Available from: https://www.ncbi.nlm.nih.gov/books/NBK554542/

## Supporting information

S1 File Consort checklist. (DOCX)

S2 File Tidier checklist. (DOCX)

S3 File Details of methods legend. This file details: Intervention procedures and protocol deviations; Health problems, as listed on ADRe; Sample size; randomization.
(DOCX)

S4 File Legend. This file contains: S1 Table. Case reports of ADRe implementation; S2 Table. Change in symptoms and actions between first and second ADRe administration; Appendix to Table 5: Parameters of Regression models.
(DOCX)

S5 File Description of folder containing interview transcripts. Legend. The folder of interview transcripts is available by application to Swansea University library. The non-author contact is: library-digital@swansea.ac.uk. The author contact is s.e.jordan@swansea.ac.uk.
(DOCX)

## Abbreviations

CI: Confidence intervals
GP: General practitioner / general practice (primary care)
GLMM: Generalised linear mixed model
ID: Identity of patient participant
NHS: National Health Service (UK)
OR / aOR: Odds ratio / adjusted odds ratio
RCT: Randomized controlled trial
SD: Standard deviation
WHO: World Health Organisation
WIMD: Welsh Index of Multiple Deprivation
