# Supplementary material for: “A better system” – managing the adverse effects of prescribed medicines: Cluster-randomised controlled trial and process evaluation of the ADRe Profile in primary care
Source: PLoS One. 2026 Sep 18;21(9):e0356480. doi: 10.1371/journal.pone.0356480 (PMC13588343; doi:10.1371/journal.pone.0356480)
Supplement: Supplementary file 1 [file pone.0356480.s001.docx]

S1 File: CONSORT Cluster-randomised trial checklist

 CONSORT 2010 checklist of information to include when reporting a cluster randomised trial

| Section/topic and item No | Standard checklist item | Extension for cluster designs | Page number/section |
| --- | --- | --- | --- |
| **Title and abstract** | | | |
| 1a | Identification as a randomised trial in the title | Identification as a cluster randomised trial in the title | Title page 1 |
| 1b | Structured summary of trial design, methods, results, and conclusions (for specific guidance see CONSORT for abstracts) |  | Abstract pages  2,3 |
| **Introduction** | | | |
| Background and objectives: |  |  |  |
| 2a | Scientific background and explanation of rationale | Rationale for using a cluster design | Background  4-5 |
| 2b | Specific objectives or hypotheses | Whether objectives pertain to the cluster level, the individual participant level, or both | Background  5 |
| **Methods** | | | |
| Trial design: |  |  |  |
| 3a | Description of trial design (such as parallel, factorial) including allocation ratio | Definition of cluster and description of how the design features apply to the clusters | Trial design  6  See protocol paper Logan et al 2022 [44] |
| 3b | Important changes to methods after trial commencement (such as eligibility criteria), with reasons |  | Not applicable |
| Participants: |  |  |  |
| 4a | Eligibility criteria for participants | Eligibility criteria for clusters | Settings and participants  Inclusion criteria  6 |
| 4b | Settings and locations where the data were collected |  | Settings and participants 6,7 |
| Interventions: |  |  |  |
| 5 | The interventions for each group with sufficient details to allow replication, including how and when they were administered | Whether interventions pertain to the cluster level, the individual participant level, or both | Intervention  7  See also references [46] and [45] |
| Outcomes: |  |  |  |
| 6a | Completely defined prespecified primary and secondary outcome measures, including how and when they were assessed | Whether outcome measures pertain to the cluster level, the individual participant level, or both | Study outcomes  7-8 |
| 6b | Any changes to trial outcomes after the trial commenced, with reasons |  | Not applicable |
| Sample size: |  |  |  |
| 7a | How sample size was determined | Method of calculation, number of clusters(s) (and whether equal or unequal cluster sizes are assumed), cluster size, a coefficient of intracluster correlation (ICC or *k*), and an indication of its uncertainty | Sample size calculation  8, S3 file, reference [44]  S3 file, S3 Table. Note to Table 5 |
| 7b | When applicable, explanation of any interim analyses and stopping guidelines |  | Not applicable |
| **Randomisation** | | | |
| Sequence generation: |  |  |  |
| 8a | Method used to generate the random allocation sequence |  | Randomisation  9, S3 file |
| 8b | Type of randomisation; details of any restriction (such as blocking and block size) | Details of stratification or matching if used | Randomisation  9, S3 file, reference [44] |
| Allocation concealment mechanism: |  |  |  |
| 9 | Mechanism used to implement the random allocation sequence (such as sequentially numbered containers), describing any steps taken to conceal the sequence until interventions were assigned | Specification that allocation was based on clusters rather than individuals and whether allocation concealment (if any) was at the cluster level, the individual participant level, or both | Randomisation  9  S3 file |
| Implementation: |  |  |  |
| 10 | Who generated the random allocation sequence, who enrolled participants, and who assigned participants to interventions | Replaced by 10a, 10b, and 10c | Randomisation  9, S3 file |
| 10a |  | Who generated the random allocation sequence, who enrolled clusters, and who assigned clusters to interventions | Settings and participants Randomisation  9 |
| 10b |  | Mechanism by which individual participants were included in clusters for the purposes of the trial (such as complete enumeration, random sampling) | Settings and participants  Inclusion criteria  6,7 |
| 10c |  | From whom consent was sought (representatives of the cluster, or individual cluster members, or both) and whether consent was sought before or after randomisation | Ethics  10  Before randomisation |
| Blinding: |  |  |  |
| 11a | If done, who was blinded after assignment to interventions (for example, participants, care providers, those assessing outcomes) and how |  | 9, Limitations, 31 |
| 11b | If relevant, description of the similarity of interventions |  | Not applicable |
| Statistical methods: |  |  |  |
| 12a | Statistical methods used to compare groups for primary and secondary outcomes | How clustering was considered | Statistical analysis  9 |
| 12b | Methods for additional analyses, such as subgroup analyses and adjusted analyses |  | Statistical analysis  9-10 |
| **Results** | | | |
| Participant flow (a diagram is strongly recommended): |  |  |  |
| 13a | For each group, the numbers of participants who were randomly assigned, received intended treatment, and were analysed for the primary outcome | For each group, the numbers of clusters that were randomly assigned, received intended treatment, and were analysed for the primary outcome | Fig 1  11 |
| 13b | For each group, losses, and exclusions after randomisation, together with reasons | For each group, losses, and exclusions for both clusters and individual cluster members | Participant flow and recruitment  Fig 1  11 |
| Recruitment: |  |  |  |
| 14a | Dates defining the periods of recruitment and follow-up |  | Trial design  6 |
| 14b | Why the trial ended or was stopped |  | Not applicable |
| Baseline data: |  |  |  |
| 15 | A table showing baseline demographic and clinical characteristics for each group | Baseline characteristics for the individual and cluster levels as applicable for each group | Tables 1,2  11 |
| Numbers analysed: |  |  |  |
| 16 | For each group, number of participants (denominator) included in each analysis and whether the analysis was by original assigned groups | For each group, number of clusters included in each analysis | Participant flow and recruitment  11  Tables 3-5  11-14 |
| Outcomes and estimation: |  |  |  |
| 17a | For each primary and secondary outcome, results for each group, and the estimated effect size and its precision (such as 95% confidence interval) | Results at the individual or cluster level as applicable and a coefficient of intracluster correlation (ICC or *k*) for each primary outcome | Outcomes  12-24  Tables 3-6  ICC S4 file, S3 table, for primary outcome Table 5 |
| 17b | For binary outcomes, presentation of both absolute and relative effect sizes is recommended |  | Tables 4-5 |
| Ancillary analyses: |  |  |  |
| 18 | Results of any other analyses performed, including subgroup analyses and adjusted analyses, distinguishing prespecified from exploratory |  | Clinical gains, costs, process evaluation  19-24 |
| Harms: |  |  |  |
| 19 | All important harms or unintended effects in each group (for specific guidance see CONSORT for harms 106) |  | Harms  25 |
| **Discussion** | | | |
| Limitations: |  |  |  |
| 20 | Trial limitations, addressing sources of potential bias, imprecision, and, if relevant, multiplicity of analyses |  | Strengths and imitations  29-31 |
| Generalisability: |  |  |  |
| 21 | Generalisability (external validity, applicability) of the trial findings | Generalisability to clusters and/or individual participants (as relevant) | Strengths and limitations  30-31 |
| Interpretation: |  |  |  |
| 22 | Interpretation consistent with results, balancing benefits, and harms, and considering other relevant evidence |  | Discussion  25-32 |
| **Other information** | | | |
| Registration: |  |  |  |
| 23 | Registration number and name of trial registry |  | 34 |
| Protocol: |  |  |  |
| 24 | Where the full trial protocol can be accessed, if available |  | Reference [44]  34 |
| Funding: |  |  |  |
| 25 | Sources of funding and other support (such as supply of drugs), role of funders |  | Funding  With journal |

*Page numbers optional depending on journal requirements.
