# Supplementary material for: “A better system” – managing the adverse effects of prescribed medicines: Cluster-randomised controlled trial and process evaluation of the ADRe Profile in primary care
Source: PLoS One. 2026 Sep 18;21(9):e0356480. doi: 10.1371/journal.pone.0356480 (PMC13588343; doi:10.1371/journal.pone.0356480)
Supplement: Supplementary file 2 [file pone.0356480.s002.docx]

S2 File: TIDIER checklist – RCT

**The TIDieR (Template for Intervention Description and Replication) Checklist*:**

Information to include when describing an intervention and the location of the information

| **Item number** | **Item** | **Where located **** |
| --- | --- | --- |
|  |  | Page  Number |
|  | **BRIEF NAME** |  |
| **1.** | The ADRe Profile |  |
|  | **WHY** |  |
| **2.** | ADRs are a major burden on the NHS and the service users. An instrument that supports real-time person-centred identification of ADRs and facilitates addressing the problems may enable early recognition of any issues, before they develop into larger and more costly problems. | 4-5 |
|  | **WHAT** |  |
| **3.** | Materials:  <https://www.swansea.ac.uk/adre/> |  |
| **4.** | Procedures: Step \| \| Performed by; Participant selection \| \| Pharmacist; Participant approach \| \| Pharmacist; Consent \| \| Researcher; Randomisation \| \| (Swansea Clinical Trials Unit [SCTU]; **Round 1** \| \|; Intervention arm \| Control arm \|; Access nursing and medical notes from consented participants to try to identify any signs and symptoms that would populate the ADRe Profile. \| \| Researchers; Obtain list of medicines (from GP practice) \| \| Researchers; Check what is being taken from list (phone call) \| Usual care \| Researcher; ADRe to patient (service users are asked to complete the ADRe Profile independently – forms to be sent electronically or by post – SAE provided \| \| Researcher; ADRe self-completion (service users complete and pass to researcher) \| \| Service users; Nurse researcher ADRe Profile completion (within 2 weeks of service user self-completion). Ddone in person in general practice or the patients’ homes, if more suitable. If not possible, telephone call completion. \| \| Researcher; Completed ADRe passed to the cluster pharmacist, pharmacist passed recommendations to a GP, service users and researcher \| \| Pharmacist; **Round 2** \| \|; Intervention arm \| Control arm \|; Access nursing and medical notes from consented participants to try to identify any signs and symptoms that would populate the ADRe Profile. \| \| Researcher; Obtain list of medicines (from GP practice) \| \| Researcher; Second ADRe completion with the nurse researcher, 4-8 weeks later after Round 1 completion \| Usual care \| Researcher; Service user and pharmacist debrief \| \| Researcher | 5 references [45, 46]  7  9, S3 file  6-8 |
|  | **WHO PROVIDED** |  |
| **5.** | Researcher – qualified nurse with 10 years of practice experience and 6 years of academic experience.  Pharmacist – qualified pharmacist, employed as a cluster pharmacist. Initial ADRe training provided through Zoom platform (duration: 28 minutes). The pharmacist felt that ADRe was self-explanatory. Researcher was available and in regular contact throughout the duration of the trial to answer any queries and uncertainties regarding the use of the ADRe Profile.  Service user participants were given information about the research project and instructions on completion of the ADRe Profile. | 7, 21  S3 file: Intervention procedures. |
|  | **HOW** |  |
| **6.** | Procedure \| Frequency per participant \| Expected duration \| Conducted by \| Place and mode of delivery; Informed consent \| 1 \| 15 mins + time to read and consider participant information \| Researcher \| Telephone and post; Educational package for participants \| 1 \| 10 minutes + time to read material \| Researcher \| Telephone and post; ADRe Profile self-completion \| 1 \| 10 – 25 mins \| service users \| By post; ADRe Profile completion with a nurse-researcher \| 2 \| 10 – 30 mins \| Researcher \| GP surgery/patient’s home; Debrief \| 1 \| 15 mins \| researchers \| Telephone/ post20 |  |
|  | **WHERE** |  |
| **7.** | Primary care in one valley in South-West Wales | 6 |
|  | **WHEN and HOW MUCH** |  |
| **8.** | As above |  |
|  | **TAILORING** |  |
| **9.** | N/A |  |
|  | **MODIFICATIONS** |  |
| **10.^ǂ^** | Nurse researcher delivered the intervention instead of the practice nurse / support worker. |  |
|  | **HOW WELL** |  |
| **11.** | Planned: Intervention adherence or fidelity was assessed by the author, by application of a modified version of the Conceptual Framework for Implementation Fidelity (Carroll et al., 2007; Hasson, 2010).  Facilitation strategies: training, instructions for completion, confidentiality. |  |
| **12.^ǂ^** | Actual:  Completeness of the ADRe Profile – excellent for all sections apart from ‘*Vital signs’,* which had very good completion rate. All ADRe reports escalated to a pharmacist, who suggested at least one action for all participants. Not all actions were implemented within the time frame for data collection. | Logan V, Hughes D, Turner A, Carter N, Jordan S. Breaking barriers: feasibility of a cluster randomised trial evaluating an instrument for identifying and ameliorating adverse drug reactions. BMJ Open. 2026 Jan 27;16(1):e099627. doi: 10.1136/bmjopen-2025-099627. PMID: 41592836. [e099627.full.pdf](https://bmjopen.bmj.com/content/bmjopen/16/1/e099627.full.pdf) **and supplementary material** |

** **Authors** - use N/A if an item is not applicable for the intervention being described. **Reviewers** – use ‘?’ if information about the element is not reported/not sufficiently reported.

† If the information is not provided in the primary paper, give details of where this information is available. This may include locations such as a published protocol or other published papers (provide citation details) or a website (provide the URL).

ǂ If completing the TIDieR checklist for a protocol, these items are not relevant to the protocol and cannot be described until the study is complete.

* We strongly recommend using this checklist in conjunction with the TIDieR guide (see *BMJ* 2014;348: g1687) which contains an explanation and elaboration for each item.

* The focus of TIDieR is on reporting details of the intervention elements (and where relevant, comparison elements) of a study. Other elements and methodological features of studies are covered by other reporting statements and checklists and have not been duplicated as part of the TIDieR checklist. When a **randomised trial** is being reported, the TIDieR checklist should be used in conjunction with the CONSORT statement (see [www.consort-statement.org](http://www.consort-statement.org)) as an extension of **Item 5 of the CONSORT 2010 Statement.** When a **clinical trial** **protocol** is being reported, the TIDieR checklist should be used in conjunction with the SPIRIT statement as an extension of **Item 11 of the SPIRIT 2013 Statement** (see [www.spirit-statement.org](http://www.spirit-statement.org)). For alternate study designs, TIDieR can be used in conjunction with the appropriate checklist for that study design (see [www.equator-network.org](http://www.equator-network.org)).
