# Supplementary material for: “A better system” – managing the adverse effects of prescribed medicines: Cluster-randomised controlled trial and process evaluation of the ADRe Profile in primary care
Source: PLoS One. 2026 Sep 18;21(9):e0356480. doi: 10.1371/journal.pone.0356480 (PMC13588343; doi:10.1371/journal.pone.0356480)
Supplement: Supplementary file 3 [file pone.0356480.s003.docx]

# S3 file: details of methods

Sections:

## 1. Intervention procedures

## 2. Health problems, as listed on ADRe

## 3. Sample size

## 4. Randomisation

## 1. Intervention procedures

| Procedure | Frequency per participant | Expected duration | Conducted by | Place and mode of delivery | Protocol deviations |
| --- | --- | --- | --- | --- | --- |
| Informed consent | 1 | 15 mins + time to read and consider participant information | Researcher | Telephone and post | None |
| Educational package for participants | 1 | 10 minutes + time to read materials | Researcher | Telephone and post | None |
| ADRe Profile self-completion | 1 | 10 – 25 mins | Service users | By post | None |
| ADRe Profile completion with a nurse-researcher | 2 | 10 – 30 mins | Researcher | GP surgery/patient’s home | Six:   - two patients took >1 hour to complete the ADRe Profile, because they raised clinical concerns needing advice; - the interval between the first and second rounds of the study exceeded 12 weeks in 22/27 patients, increasing to 20 weeks due to delays in booking rooms in primary care; 16 were followed-up at 17-20 weeks;   Missing data from the signs and symptoms analysed:   - the ADRe item on reproductive health was omitted for 15 patients, due to concerns regarding cultural sensitivity; no other questions had missing data.   Missing data on vital signs:   - vital signs could not be fully assessed by the researcher for 5 patients. All patients had a sitting BP, but for 1 participant, this was as reported from clinic attendance. Standing BP was not recorded for 4 (R1) and 5 (R2) participants: 2 were unable to stand; 1 completed ADRe by telephone, and on 1 and 2 occasions the measurement failed. - Oxygen saturation was not measured on one occasion, because the pulse oximeter battery was exhausted. It was not reported by the participant who responded by telephone. - Only patients attending the surgery had their weight measured. It was impractical to use portable scales and ensure appropriate calibration. Some patients knew their weight or BMI, and self-reported weights were used. Eight patients had no data for weight or BMI. This item was not analysed further*. |
| Debrief | 1 | 15 mins | Researcher | Telephone/ post | One participant completed ADRe by telephone only, and vital signs were as reported. This participant did not complete the process evaluation. |

*Weight and BMI information was collected once: we felt it was unlikely to change significantly in adults over 12 weeks.

## 2. Health problems as listed on ADRe

Only items listed in sections 2 and 3 were taken as problems to be analysed from the medical records. Recording of vital signs was analysed separately (Table 4). We did not assess vital signs. The information was passed to the pharmacist, and used in the descriptions of problems where necessary (Tables 6, S1, S2). We did not expect problems with medicines administration, health promotion or qualitative comments to be noted in medical records and did not record them. Of the 61 problems in sections 2 & 3, the 2 questions on pregnancy were discounted. The questions on catheters and non-verbal signs of pain proved irrelevant in this patient group, leaving 57 items for consideration.

## Health Problems as listed on ADRe

| **Vital signs** | | | | | | | | **Comments** | | | | |  |
| --- | --- | --- | --- | --- | --- | --- | --- | --- | --- | --- | --- | --- | --- |
| Heart rate sitting | | | Bpm | | | | |  | | | | |  |
| Irregular rhythm | | | | | | | |  | | | | |  |
| BP lying/ sitting | | | / mmHg | | | | |  | | | | |  |
| Change since last recording | | | / mmHg | | | | |  | | | | |  |
| BP standing | | | / mmHg | | | | |  | | | | |  |
| Change since last recording | | | / mmHg | | | | |  | | | | |  |
| Heart rate standing | | | Bpm | | | | |  | | | | |  |
| BMI | | | kg/m^2^ | | | | |  | | | | |  |
| Weight | | | st kg | | | | |  | | | | |  |
| Change since last recording | | | | | | | |  | | | | |  |
| Oxygen saturation | | | % | | | | |  | | | | |  |
| Change since last recording | | | % | | | | |  | | | | |  |
| ECG available / done in last year | | | | | | | |  | | | | |  |
| Lung function tests available / any record | | | | | | | |  | | | | |  |
| Observations | | | | | | | |  | | | | |  |
| Hand tremor affecting drinking OR eating OR fastening buttons / zips | no / yes / worse | | | | |  | | | | |  |  |  |
| Tongue: abnormal movements or tremor | no / yes / worse | | | | |  | | | | |  |  |  |
| Feet shuffling | no / yes / worse | | | | |  | | | | |  |  |  |
| Abnormal movements at rest | no / yes / worse | | | | |  | | | | |  |  |  |
| Posture abnormal | no / yes / worse | | | | |  | | | | |  |  |  |
| Gait abnormal on walking | no / yes / worse | | | | |  | | | | |  |  |  |
| Balance/ co-ordination poor/ any ‘Activities of Daily Living’ affected | no / yes / worse | | | | |  | | | | |  |  |  |
| Cognitive decline (memory or concentration problem) | no / yes / worse | | | | |  | | | | |  |  |  |
| Feeling the cold / cold to touch | no / yes / worse | | | | |  | | | | |  |  |  |
| **Skin: observation or questions** |  | | | | |  | | | | |  |  |  |
| ANY bleeding/ bruising/ nosebleeds | no / yes / worse | | | | |  | | | | |  |  |  |
| Rash (+/- itching) | | no/ yes / worse | | | |  | | | | |  |  |  |
| Swelling/ oedema | | no / yes /worse | | | |  | | | | |  |  |  |
| Sweating | | no / yes /worse | | | |  | | | | |  |  |  |
| Acne / hirsutism / *herpes simplex* (cold sores) | | no / yes /worse | | | |  | | | | |  |  |  |
| Broken skin / poor healing | | no / yes /worse | | | |  | | | | |  |  |  |
| Hair loss | | no / yes /worse | | | |  | | | | |  |  |  |
| Other skin abnormalities e.g. change in skin colour | | no / yes | | | |  | | | | |  |  |  |
| Injection site problems e.g. pain | | no / yes | | | |  | | | | |  |  |  |
|  | |  | | | |  | | | | |  |  |  |
| **Reports / questions** | | | | | | |  | | | | |  |  |
| Any convulsions or seizures (even if epileptic) | | no / yes /worse | | | |  | | | | |  |  |  |
| Behaviour problems | | no / yes /worse | | | |  | | | | |  |  |  |
| Self-harm | | no / yes /worse | | | |  | | | | |  |  |  |
| Physical violence to people or objects | | no / yes /worse | | | |  | | | | |  |  |  |
| Aggression (physical / verbal) / irritability | | no / yes /worse | | | |  | | | | |  |  |  |
| Agitation/ anxiety/ nervousness / hyperactivity | | no / yes /worse | | | |  | | | | |  |  |  |
| Restlessness or pacing | | no / yes /worse | | | |  | | | | |  |  |  |
| Panic attacks | | no / yes /worse | | | |  | | | | |  |  |  |
| Confusion | | no / yes /worse | | | |  | | | | |  |  |  |
| Mood fluctuations – high or low moods | | no / yes /worse | | | |  | | | | |  |  |  |
| Low energy, weakness, fatigue, apathy | | no / yes /worse | | | |  | | | | |  |  |  |
| Hallucinations / vivid dreams | | no / yes /worse | | | |  | | | | |  |  |  |
| Sleep problems/ insomnia (inability to sleep) | | no / yes /worse | | | |  | | | | |  |  |  |
| Sedation / excessive sleep >2 hours over normal | | no / yes /worse | | | |  | | | | |  |  |  |
| Dizziness | | no / yes / worse | | | |  | | | | |  |  |  |
| Falls | | no / yes / worse | | | |  | | | | |  |  |  |
| Headache / migraine | | no / yes /worse | | | |  | | | | |  |  |  |
| **Pain:** Any pain? E.g. joint pain. | | no / yes /worse | | | |  | | | | |  |  |  |
| Non-verbal pain indicators present? E.g. aggression, moaning, curling up, laboured breathing | | no / yes /worse | | | |  | | | | |  |  |  |
| Tingling / pins & needles | | no / yes /worse | | | |  | | | | |  |  |  |
| Tinnitus / hearing problems | | no / yes /worse | | | |  | | | | |  |  |  |
| **Vision:** eyesight problems | | no / yes /worse | | | |  | | | | |  |  |  |
| Dry eyes | | no / yes /worse | | | |  | | | | |  |  |  |
| Urination problems /incontinence / burning / discomfort on urination / UTI (urinary tract infection) | | no / yes /worse | | | |  | | | | |  |  |  |
| Urinary catheter: short/ long-term | | no/ yes | | | |  | | | | |  |  |  |
| *Discretionary question:* Reproductive system e.g. breast discomfort/erectile dysfunction/ libido (sex drive) change | | no / yes /worse | | | |  | | | | |  |  |  |
| Is the service user a **woman of childbearing age?** | | no / yes | | | |  | | | | |  |  |  |
| Implications for pre-conception care discussed? | | NA /yes / no | | | |  | | | | |  |  |  |
| Chest pain | | no / yes /worse | | | |  | | | | |  |  |  |
| Short of breath/ unable to finish sentences | | no / yes /worse | | | |  | | | | |  |  |  |
| High salt intake? | | no /yes | | | |  | | | | |  |  |  |
| **Oral health** Problems with teeth or dentures | | no / yes | | | |  | | | | |  |  |  |
| Dry mouth | | no / yes /worse | | | |  | | | | |  |  |  |
| Halitosis (bad breath) | | no / yes | | | |  | | | | |  |  |  |
| Hypersalivation /change of clothes due to drooling /wet pillow | | no / yes /worse | | | |  | | | | |  |  |  |
| Swallowing difficulties | | no /yes /worse | | | |  | | | | |  |  |  |
| Indigestion or heartburn | | no /yes / worse | | | |  | | | | |  |  |  |
| Nausea / vomiting | | no / yes /worse | | | |  | | | | |  |  |  |
| Appetite/ taste changes | | no / yes /worse | | | |  | | | | |  |  |  |
| Bowel control/ diarrhoea | | no / yes /worse | | | |  | | | | |  |  |  |
| Constipation (Bowels not open in last 36 hours) | | no / yes /worse | | | |  | | | | |  |  |  |
| Sore throat and raised temperature | | no / yes | | | |  | | | | |  |  |  |
| Respiratory problems developing. Any 1 or more of: wheeze / cough / chest tightness / short of breath | | no / yes /worse | | | |  | | | | |  |  |  |
| **Medicines Administration** | | | | |  | | | | |  |  |  |  |
| Any tablets crushed or broken? | | | |  | | | | |  |  |  |  |  |
| Regular medication taken at the same time each day? | | | |  | | | | |  |  |  |  |  |
| More than 2 doses of prescribed medication missed in the last seven days. (including refusal) | | | |  | | | | |  |  |  |  |  |
| **Medicines** bought without a prescription, include herbal preparations | | | |  | | | | |  |  |  |  |  |
| Recreational drug use (include legal highs) [optional] | | | |  | | | | |  |  |  |  |  |
| Alcohol over-use (>2 drinks on 1 occasion) [optional] | | | |  | | | | |  |  |  |  |  |
|  | | | | | | | |  | | | | | |
| Are there any other problems you would like to note? | | | |  | | | | |  |  |  |  |  |
| Thinking about your health, what is the most important problem for you, the service user?  *B* | | | |  | | | | |  |  |  |  |  |
| **Prevention and Health Promotion** | | | |  | | | | |  |  |  |  |  |
| **Vision** Optician visit in the last 12 months | | | |  | | | | |  |  |  |  |  |
| **Oral health** Dentist visit in last 12 months | | | |  | | | | |  |  |  |  |  |
| Smoking | | | |  | | | | |  |  |  |  |  |
| Changes in smoking | | | |  | | | | |  |  |  |  |  |
| **Hydration**.  Drinking at least 6-8 cups/ day? Includes water, tea, etc | | | |  | | | | |  |  |  |  |  |
| **Diet** ‘Snacking’ or eating between meals? | | | |  | | | | |  |  |  |  |  |
| Are drinks (including tea) sugar free? | | | |  | | | | |  |  |  |  |  |
| **Meals** missed or left unfinished more than once a day? | | | |  | | | | |  |  |  |  |  |
| 2 or more meals (1 cooked) eaten daily on 6 of last 7 days. | | | |  | | | | |  |  |  |  |  |
| Eating fruit or vegetables every day? | | | |  | | | | |  |  |  |  |  |
| F**racture Prevention** Takes at least 1 pint of milk/ day? Include milk added to drinks/meals/cereals | | | |  | | | | |  |  |  |  |  |
| Vitamin D intake adequate (sunlight exposure, eating oily fish) | | | |  | | | | |  |  |  |  |  |
| **Sun exposure:** Is 4 stars, high factor **sunscreen** available? | | | |  | | | | |  |  |  |  |  |
| Are dark glasses available to wear in bright sunlight? | | | |  | | | | |  |  |  |  |  |
| Immunisations up to date? | | | |  | | | | |  |  |  |  |  |

## 3. Sample size

The calculation of the sample size has been published (Logan et al 2022) and is reproduced here.

In relation to the primary outcome of the number and nature of problems addressed (clinical gains) following ADRe implementation, a sample of 60 service users in 6 clusters was considered adequate in this trial. Previously, in a ‘before and after’ single arm intervention study, with participants acting as their own controls, 17/19 (89%) had at least one clinical problem addressed when ADRe Profiles for polypharmacy were used (Jordan et al., 2021). We hypothesised that 9/19 (47%) would have shown some improvement without intervention, necessitating an effective sample of 40 patients (20 in each arm), with 5% significance and 90% power (Uitenbroek 1997). The data provided by a sample in a cluster-randomised trial is usually thought to provide less information than if the sample were randomised by individual, due to the tendency of individuals within a cluster to share more similarities than individuals from different clusters (Cornfield, 1978). Based on the reported intra-cluster coefficient (0.02), (Jordan et al., 2015), and a design effect of 1.18, to achieve an effective sample size of 40, an actual sample size of 48 was needed (Killip et al 2004). Ten patients in each of 6 clusters (GP practices) allows for 20% loss to follow up, which appears realistic in primary care, where either patient or healthcare professional may be unavailable for follow up.

## 4. Randomisation

Firstly, a pre-randomisation survey of the participating GP surgeries was performed. The 6 participating GP surgeries were stratified according to the socio-economic status of the population the GP practices serve, using the Welsh Index of Multiple deprivation ([WIMD], WG, 2019). The WIMD was based on the GP practice postcode. This strategy aimed to reduce the variability between clusters and improve the precision of the study (Campbell and Walters, 2014). The WIMD score has been selected as having the most substantial prognostic importance and other potential stratification factors (such as obtaining baseline data on other important prognostic variables, such as the number of long-term conditions) have not been utilised. Downs and colleagues (2010) warn against choosing too many stratification factors, which may cause ‘over-stratification’ in small trials, thus potentially degrading the balance.

The GP surgeries were randomised using random number generating software (EXCEL) within the relevant strata and they were equally assigned to intervention or control arms. To avoid allocation bias, randomisation was undertaken independently of the PhD team by Prof. Alan Watkins, professor of statistics in the Swansea Trials Unit. Consenting practices were numbered C3 to C8, and the list retained by the researcher (VL). Each practice was assigned a value for socio-economic status, based on the practice’s postcode. Where practices had >1 surgery, the data for the surgery being used was taken. The WIMD socio-economic status score, Wales rank and quintile was ascertained for each practice using look-up tables. All 3 measures were assigned to each practice. The statistician received the numbers 1-6 with socio-economic status assigned. The six practices were then randomised to intervention or control arms, stratified by the socio-economic status at the discretion of the statistician.

It was agreed that P6 and P8 would be randomised as a pair due to uneven distribution of participants (see the Results section for further details on the protocol deviation), to ensure a balanced allocation. With the need to stratify by WIMD, this pair could have been combined with any one of C3, C4, C5 in one arm, with the other three (C7, and the reaming two from C3, C4, C5) in the other arm. One arm would be allocated to ADRe; the other to Control.

There were thus six possibilities:

(P3, P6, P8) to ADRE

(P4, P6, P8) to ADRE

(P5, P6, P8) to ADRE

(P3, P6, P8) to Control

(P4, P6, P8) to Control

(P5, P6, P8) to Control

A simple selection process in EXCEL (using a non-repeatable seed) was implemented by the statistician; after testing it (satisfactorily) for randomness, the statistician then generated the actual randomisation. Excel assigned each of the 6 options a value between 0 and 1 at random via the function (=RAND()). The option with the highest value was automatically selected using the function (IF [named cell]=MAX(cell range),1,0). The EXCEL spreadsheet is available from the researcher on request, a copy of the allocation sequence is included in Appendix 39. The random allocation generated was P4, P6, P8 to ADRE, and P3, P5, P7 to Control.

To avoid selection bias and enable allocation concealment, GP practices were selected, and patients consented before randomisation, as recommended by Puffer, Torgerson & Watson (2003). While masking was not possible due to the design of the study, ascertainment bias was lowered by researchers not being aware of the randomisation results until after they have accessed the nursing and medical notes and elicited baseline data.

Consent was sought at the individual participant level, before randomisation, but after expressions of interest were received from potential participants.

## References

Cornfield J. (1978). Randomization by group: A formal analysis. American Journal of Epidemiology, 108(2), 100–102. https://doi.org/10.1093/oxfordjournals.aje.a112592

Jordan, S., Banner, T., Gabe-Walters, M., Mikhail, J.M., Panes, G., Round, J., Snelgrove, S., Storey, M., & Hughes, D. (2019). Nurse-led medicines’ monitoring in care homes, implementing the Adverse Drug Reaction (ADRe) Profile improvement initiative for mental health medicines: An observational and interview study. PloS One, 14(9), e0220885–e0220885. https://doi.org/10.1371/journal.pone.0220885

Jordan, S., Gabe-Walters, M.E., Watkins, A., Humphreys, I., Newson, L., Snelgrove, S., & Dennis, M.S. (2015). Nurse-Led Medicines' Monitoring for Patients with Dementia in Care Homes: A Pragmatic Cohort Stepped Wedge Cluster Randomised Trial. PloS One, 10(10), e0140203–e0140203. https://doi.org/10.1371/journal.pone.0140203

Killip S, Mahfoud, Z., & Pearce, K. (2004). What is an intracluster correlation coefficient? Crucial concepts for primary care researchers. Annals of Family Medicine, 2(3), 204–208. https://doi.org/10.1370/afm.141

Logan, V., Bamsey, A., Carter, N., Hughes, D., Turner, A. & Jordan, S. (2022). Clinical Impact of Implementing a Nurse-Led Adverse Drug Reaction Profile in Older Adults Prescribed Multiple Medicines in UK Primary Care: A Study Protocol for a Cluster-Randomised Controlled Trial. Pharmacy; 10(3):52. [https://doi.org/10.3390/pharmacy10030052](https://eur03.safelinks.protection.outlook.com/?url=https%3A%2F%2Fdoi.org%2F10.3390%2Fpharmacy10030052&data=05%7C01%7CS.E.Jordan%40Swansea.ac.uk%7Ca4f3e063f43e44a26d6308da293d27ca%7Cbbcab52e9fbe43d6a2f39f66c43df268%7C0%7C0%7C637867639870774562%7CUnknown%7CTWFpbGZsb3d8eyJWIjoiMC4wLjAwMDAiLCJQIjoiV2luMzIiLCJBTiI6Ik1haWwiLCJXVCI6Mn0%3D%7C3000%7C%7C%7C&sdata=MNgqWVeBxqDsTHyGzO%2F9JUlkFbEsGrr2gA2ne5TAXfo%3D&reserved=0)

Uitenbroek, D.G. Correlation: SISA Home. 1997. Available online: <http://www.quantitativeskills.com/sisa/> (accessed on 15 December 2021).
