# Supplementary material for: “A better system” – managing the adverse effects of prescribed medicines: Cluster-randomised controlled trial and process evaluation of the ADRe Profile in primary care
Source: PLoS One. 2026 Sep 18;21(9):e0356480. doi: 10.1371/journal.pone.0356480 (PMC13588343; doi:10.1371/journal.pone.0356480)
Supplement: Supplementary file 4 [file pone.0356480.s004.docx]

# S4 file. Additional Data Tables

S1 Table. Case reports of ADRe implementation

S2 Table. Change in symptoms and actions between first and second ADRe administration

S3 Table. GLMM with model parameters

Appendix to Table 5: Tables S5a-e. Parameters of Regression Models

## S1 table: Case reports of ADRe implementation

|  | Medicines prescribed | Problems identified (selected) | Practice changes | Participant gain | Participant comments |
| --- | --- | --- | --- | --- | --- |
| 1 | Warfarin 3mg, take as per INR  Warfarin 1 mg, take as per INR  Simvastatin 40mg, 1 nocte  Eplerenone 25 mg, 1 OD  Flecainide 100mg, 1 BD  Bisoprolol 10 mg, 1nocteRamipril 10 mg caps, 1 OD | Irregular heart rhythm  Bleeding, bruising easily  Oedema (legs)  Cold sores  Sleep problems Vivid nightmare-type dreams  Pain | Laboratory tests  Vital signs recorded  GP review  Warfarin dose review  Deprescribed: Flecainide  New medicines: Prostap® injections (leuprorelin), Bumetanide | 1. Pain resolved  2. Prostate cancer diagnosed at an early stage and treated  3. Vivid dreams resolved | *‘It (ADRe) makes sense, although I always read medication leaflets, they are sometimes confusing.’* |
|  |  |  |  |  |  |
|  | Potential for further management, identified by academic team:  1. Leg elevation advice to reduce oedema;  2. Artificial saliva OTC to reduce xerostomia;  3. Flecainide possible cause of arrhythmias/ tinnitus;  4. Inform pt about link between symptoms of cold, abnormal dreams and lack of energy possibly due to essential bisoprolol;  5. Statin may contribute to poor sleep;  6. Tinnitus may be due to flecainide or ramipril | | | | |
| 2 | Bisoprolol 10 mg, 1 OD  Isosorbide mononitrate 20 mg, 1 morning, 1 afternoon (after 6 hrs)  Aspirin 75 mg 1 OD,  Simvastatin 40 mg, 1 nocte  Omeprazole 20 mg GR caps, 1 OD  Losartan 50 mg, 1 OD  Metformin 500 mg, 2 BD before food  Victoza® (liraglutide) 6mg/ml solution for injection 3 ml pre-filled pens, 1.2 mg OD  Gliclazide 80 mg, 1 BD  Fluoxetine 20 mg caps, 1 OD  Clotrimazole 500 mg pessary and 1% cream  Metronidazole 0.75% vaginal gel | Postural hypotension  Hand tremor  Feet shuffling  Balance problems  Tingling in hands  Itching (all over)  Cognitive decline  Anxiety  Mood fluctuations  Low energy (tired all the time)  Vivid dreams  Sleep problems  Dizziness  Pain  Tingling  Tinnitus  Shortness of breath  Heartburn  Bowel control (diarrhoea) | GP review  Diabetic review  Laboratory tests undertaken  Vital signs recorded  Deprescribed: Clotrimazole, Metronidazole, Metformin  New medicines: Cyanocobalamin, Ferrous sulphate, Solifenacin, Co-codamol | 1.Pernicious anaemia diagnosed and treated  2.Hand tremor resolved  3.Balance problems resolved  4.Sleep problems resolved  5.Tingling resolved  6.Bowel problems resolved | *‘I know that there are more people involved with my health, and they will do something if I need it. It gives me peace of mind.’* |
|  | Potential for further management, identified by academic team:  1. Reviewing antihypertensives possibly causing postural hypotension (nitrates + losartan + beta blocker + SSRI + PPI + possible hypoglycaemia);  2. Beta blocker may be causing HF/ sob & masking hypo – caution in diabetes; 3.Hypoglycaemic episodes may be manifesting as a tremor or poor balance or anxiety or vivid dreams / sleep problems, paraesthesia, tinnitus;  4. Cognitive decline / depression may be due to beta blocker/ SSRI/ PPI/ statin;  5. Fluoxetine as a cause of sob/ anxiety/ sleep problems/ low energy/ paraesthesia/ tinnitus/ diarrhoea/ tremor/ posture/ movement disorders: depression persists.  6. ADRe has detected Parkinsonism, PPI continued >8 weeks & is increasing falls risk/ dizziness/ paraesthesia/ cognitive decline/ depression/ diarrhoea. Heartburn persists,  Tinnitus: check ear wax. Aspirin may be contributing to tinnitus. | | | | |
| 3 | Bisoprolol 1.25 mg, 1 BD  Apixaban 2.5, 1 BD  Bumetanide 1 mg, 1 in the morning  Simvastatin 40 mg, 1 nocte  Eplerenone 25 mg, 1 OD  Omeprazole 20 mg GR caps, 1 OD  Finasteride 5 mg, 1 OD  Sertraline 50mg, 1 ½ OD Prescriptions for short-term problems(20/04/22): Flucloxacillin, Colchicine, Maxitrol (neomycin/ polymyxin/ dexamethasone) eye drops | Balance  Feeling the cold  Excessive sweating  Mood fluctuations, low mood  Vivid dreams  Falls  Pain  Dry mouth  Diarrhoea | GP review  Laboratory tests  Vital signs recorded  New medicines: sacubitril with valsartan, dapagliflozin®, Strivit D3® (colecalciferol), Loceryl® (amorolfine) nail laquer,  Short-term prescriptions: Maxitrol® (neomycin/ polymyxin/ dexamethasone) eye drops.  Deprescribed: Bumetanide | 1.Vivid dreams stopped  2.Feeling cold stopped  3.Excessive sweating resolved  4.Diarrhoea resolved | *‘Yes, this is a better system than seeing the GP, I find that GPs are reluctant to make any changes, they follow what the hospital prescribed and are reluctant to stop any medicines. Also talking to GPs is very rigid and formal, you got more out of me than a GP would.’* |
|  | Potential for further management, identified by academic team:  Falls not resolved. Need to consider:  1. Postural hypotension – risk of falls assessment /sertraline/beta blocker/ diuretic/ PPI/ statin;  2. Low mood – if a rapid metaboliser, may be getting sertraline withdrawal symptoms. consider fluoxetine. Beta blocker essential. Consider PPI as cause.  3. Vivid dreams may be due to long sleep times or meds (SSRI, PPI, beta blocker) but unlikely to be withdrawn;  4. Hearing problems: check for ear wax. Consider bumetanide, SSRI  5. Xerostomia – consider artificial saliva preparation;  6. Diarrhoea – SSRI, PPI, statin;  7. PPI – review need as no indigestion, and possible contribution to falls, diarrhoea, dreams | | | | |
| 4 | Felodipine 2.5 mg MR, 1OD  Simvastatin 20mg, 1 nocte  Aspirin 75mg dispersible, 1 OD  Sitagliptin 100mg, 1 OD  Metformin 1g MR, 2 OD  Gliclazide 80mg, 1 BD  Ventolin 100mcg/dose, inhale 1-2 doses up to QDS, PRN  Folic acid 5mg, 1 OD  Hypromellose 0.5% eye drops PRN  Hydroxocobalamin 1mg/1ml injection, every 3 months  Terbinafine 1% cream, apply BD  Canesten cream, apply BD | Hand tremor  Feet shuffling  Abnormal posture  Balance  Cognitive decline  Feeling the cold  Oedema  Confusion  Pain  Hearing problems  Dry eyes  Urination problems  Shortness of breath  Dry mouth  Constipation | Simvastatin to Atorvastatin, stopped Felodipine for 3 weeks | 1.Ear wax removed; hearing problems resolved  2.Urination problems resolved, given pelvic floor exercises and fluids advice | *‘They (ADRe team) looked at a lot of things that wouldn’t otherwise be looked at. I am feeling looked after.’* |
|  | Potential for further management, identified by academic team:  1. Glucose monitoring to explore tremor, confusion, incontinence (salbutamol), hearing loss (consider aspirin), constipation (felodipine, immobility, Parkinson’s);  2. SOB: using only salbutamol to control lung condition – needs review, LFTs... consider gliptins, felodipine;  3. Whether B12 deficiency is due to metformin;  4. Cognitive decline/ confusion / depression, consider statin, metformin, anaemia, B12 under-replacement, hypothyroidism;  5. HF consider felodipine, B12 under-dosing / anaemia, gliptins, hypothyroidism;  6. Dry eyes, dry mouth – check inhaler technique / salbutamol use. Artificial saliva preparations | | | | |
| 5 | Donepezil 5mgs  Simvastatin 40mg 1 nocte  Candesartan 4mgs OD  Bisoprolol 2.5mgs OD  Warfarin 1mg, take as per INR  Trulicity 1.5mgs in 0.5ml prefilled pens 1 weekly (dulaglutide)  Alendronic acid 70mgs effervescent sugar free 1 weekly  Metformin 500mg x2 BD + 1 midday  Humalog mix 50 - 100iu/ ml 3ml cartridges as directed  Colecalciferol 1,000 iu OD  Co-codamol 15/500mgs 1-2 caps up to QDS | Postural hypotension  Low oxygen saturation  Hand tremor  Feet shuffling  Balance problems  Cognitive decline  Bleeding, bruising  Rash  Injection site problems  Confusion  Mood fluctuations  Low energy  Sleep problems  Dizziness  Falls  Pain  Urination problems  Swallowing difficulties  Diarrhoea | Clotrimazole cream prescribed, co-codamol stopped | 1. Advice to rotate injection sites – injection site problems resolved  2. Opioid stopped  3. Skin rash resolved  4. Sleep problems resolved  5. Dizziness resolved  6. Diarrhoea resolved | ‘*had a GP review, CT scan- waiting for results and a rash was resolved with creams… Both of us benefitted.’* (the participant and her husband participated in the study |
|  | Potential for further management, identified by academic team:  1. Low oxygen 91% should be monitored. Consider beta blocker, codeine, sleep apnoea (obese);  2. Beta blocker may be masking symptoms of hypo (note tremor);  3. Falls risk assessment, falls, dizziness: codeine, donepezil, antihypertensive;  4. Cognitive decline, confusion, mood & codeine, beta blocker, diabetes control;  5. Bleeding – paracetamol, warfarin (INR checked);  6. Rash – alendronic acid, infection (check glucose);  7. Pain – alendronic acid, stiffness from Parkinsonism;  8. Swallowing difficulties – parkinsonism | | | | |
| 6 | Gabapentin 300mgs caps 1 TDS  Levothyroxine sodium 50mcgs + 100mcgs OD  Clopidogrel 75mgs OD  Atorvastatin 40mgs OD  Zomorph 10gs mr caps 2 q. 12 hours  Paracetamol 500mgs 2 tabs q 6 hours PRN  Laxido orange OD  Acidex advance oral suspension peppermint 10ml nocte  Fostair 200 + 6 mcgs inhaler 1-2 puffs BD  Stexerol D3 1,000 iu tabs 1 x OD  Evolve eye drops carmellose 0.5% 1-2 drops PRN  Carbomer eye drops 0.2% nocte  Avamys 27.5mcgs nasal spray daily | Obesity  Abnormal movements at rest  Abnormal gait  Cognitive decline  Excessive sweating  Irritability, aggression  Agitation, anxiety  Restlessness  Panic attacks  Mood fluctuations  Low energy  Vivid dreams  Sleep problems  Falls  Pain  Tingling  Dry eyes  Urination problems  Dry mouth  Indigestion  Constipation | Mirtazapine prescribed | 1. Panic attacks, agitation, anxiety resolved  2. Sleep problems resolved  3. No further falls  4. Dry eyes resolved  5.Constipation resolved |  |
|  | Potential for further management, identified by academic team:  1. Parkinsonism – gabapentin (not mentioned above) – will be exacerbated by mirtazapine  2. Cognitive decline _ - insufficient thyroxine, morphine, gabapentin  3. Irritability _- hypoxia / poor respiratory control, pain, bronchodilators, morphine, gabapentin  4. Pain can be worsened by gabapentin, morphine, back pain by mirtazapine  5. Obesity – inhaled steroids, gabapentin  6. Agitation worsened by mirtazapine  7. Depression, asthenia – gabapentin https://bnf.nice.org.uk/drugs/gabapentin/ morphine, cortico-steroids  8. Dreams – morphine  9. Sleep problems – improved by mirtazapine – suggest re-time morphine, reduce gabapentin  10. Carpal tunnel likely hypothyroidism  11. Xerostomia – check inhaler technique & spacer  12. Gabapentin / morphine interaction and respiratory impact – review COPD (hypoxic) | | | | |
| 7 | Zapain 30/500mgs 1 up to QDS, PRN  Propanaolol MR caps 80mgs OD  Amlodipine 5mgs OD  Paracetamol 500mgs 1 q 6 hours, PRN  Omeprazole 20mgs GR OD  Levothyroxine sodium 100mcg OD  Duloxetine 30mgs GR 1 OD | Postural hypotension  Obesity  Abnormal posture  Feeling the cold  Itching  Oedema  Sweating  Anxiety  Vivid dreams  Sleep problems  Falls  Headaches  Tingling  Dry eyes  Chest pain  Short of breath  Dry mouth  Swallowing difficulties  Diarrhoea | Duloxetine® decreased from 30 to 20mcg OD | 1. Excessive sweating resolved  2. Vivid dreams and sleep problems resolved  3. No more falls in the past month  4. Headaches resolved  5. Swallowing difficulties resolved | *‘I was prescribed antidepressants which helped with sleep and pain.’* |
|  | Potential for further management, identified by academic team:  1. Postural hypotension – duloxetine, codeine;  2. Obesity – duloxetine;  3. Itching – codeine, dry skin, thyroid problems;  4. Oedema – beta blocker, calcium blocker;  5. Investigate cause for night sweats;  6. Anxiety – duloxetine;  7. PPI - ? still needed, consider dreams, paraesthesia;  8. Tingling – duloxetine, PPI, amlodipine, beta blocker;  9. Insomnia – duloxetine, codeine, beta blocker, thyroxine – consider skipping codeine in the evening;  10. Falls risk assessment;  11. Explore chest pain + sob + oedema – beta blocker, codeine;  12. Xerostomia & swallowing difficulties – artificial saliva – PPI, codeine, duloxetine;  13. Bowel control – duloxetine, beta blocker, codeine + OTC laxatives | | | | |
| 8 | Empagliflozin 10mgs 1 OD  Doxazosin 1mg 1 OD  Linagliptin 2.5mgs/ metform 1g 1 BD  Lansoprazole 30mg gr caps 1 OD  Aspirin 75mgs dispersible 1 OD  Perindopril erbumine 8mgs 1 OD  Atorvastatin 10mgs 1 OD  Felodipine 10mgs MR 1 OD  Paracetamol 500mgs 1-2 QDS  Betnovate cream 0.1% apply BD  Timodine cream TDS | Feeling the cold  Nosebleeds  Rash  Vivid dreams  Sleep problems  Pain  Vision problems  Short of breath  High salt intake  Hypersalivation  Indigestion  Alcohol overuse | Doxazosin stopped | 1. Feeling cold resolved  2. Vivid dreams and sleep problems resolved  3. Shortness of breath resolved | *‘Things have been brought to attention – the rash appointment (dermatology) was speeded up.’* |
|  | Potential for further management, identified by academic team:  1. BP 181/91 + nosebleeds (may be aspirin, paracetamol, perindopril (remote risk) or BP) – meds need review Postural drop;  2. Sob & oxygen 96% ? HF – ACEI, calcium blocker, alpha blocker;  3. review salt intake ? dietician;  4. indigestion - ACEI, calcium blocker, PPI withdrawal, alcohol, ?statin;  5. Rash – review topical preps & diabetes control ? fungal infection – any new meds started just before the rash?;  6. Dreams / insomnia – diabetes control ? nocturnal hypo , PPI, doxazosin, alcohol;  7. Vision – opticians (diabetes ? photosensitivity from perindopril);  8. ? discuss alcohol referral with GP | | | | |

Abbreviations/acronyms:

INR – internationalised

Nocte – at night

OD – once per day, BD – twice per day, TDS – three times per day, QDS – four times per day

Caps – capsules

GR – gradual release

MR – modified release

PRN – pro re nata – as needed

q - every

## S2 table. Change in symptoms and actions between first and second ADRe administration

(Intervention arm participants, n = 27)

| Problem | Round 1 number reporting | Number improving | Number worsening | Round 2  number reporting | Actions contributing to a decrease in symptoms experienced |
| --- | --- | --- | --- | --- | --- |
| Hand tremor | 4 | 1 | 0 | 3 | Patient 6 - Metformin de-prescribed (may have contributed to hypoglycaemia and tremor), B12 injections (correcting B12 deficiency, which causes tremor). |
| Abnormal movements at rest | 5 | 4 | 1 | 2 | Patient 14 – none (gabapentin a likely cause)  Patient 19 – Sukkarto® switched to metformin (possibility of correcting hypoglycaemia causing movement disorders)  Patient 26 – none  Patient 27 – mycophenolate dose corrected, reducing risk of tremor and abnormal movements. |
| Abnormal posture | 6 | 3 | 2 | 5 | Patient 16 (611) – Duloxetine decreased from 30 to 20mcg OD  Patient 16 (802) - none  Patient 27 (814) – mycophenolate dose reduced improved arthralgia, fluoxetine discontinued |
| Abnormal gait | 6 | 2 | 1 | 5 | Patient 19 – vitamin B12 deficiency corrected  Patient 27 – as above, mycophenolate dose corrected |
| Balance | 10 | 6 | 4 | 8 | Patient 2 – none  Patient 6 – cyanocobalamin and Ferrous sulphate prescription  Patient 12 – planned surgery - total knee replacement  Patient 19 – as above, B12 replacement  Patient 24 – gabapentin dose decrease, chlorphenamine de-prescribing  Patient 26 – none |
| Cognitive decline | 10 | 2 | 3 | 11 | Patient 2 (402) – anaemia treated (Ferrous fumarate prescription)  Patient 24 (811) – gabapentin dose reduced |
| Feel cold | 13 | 5 | 1 | 9 | Patient 3 – plan to review beta-blocker  Patient 5 – diuretic prescribed (addressed heart failure, and improved circulation), flecainide stopped (oedema reduced).  Patient 7 – dapagliflozin prescribed (treats heart failure)  Patient 20 – pain reduced  Patient 23 – doxazosin de-prescribing |
| Bleeding/bruising/nosebleeds | 9 | 3 | 5 | 11 | Patient 13 (607) – Co-codamol stopped  Patient 23 (810) – Doxazosin stopped  Patient 27 (814) – fluoxetine disconinued |
| Skin rash | 12 | 4 | 3 | 11 | Patient 4 – prednisolone prescription, carbamazepine stopped  Patient 9 – advice to use emollient TDS  Patient 11 – cream already prescribed; advice given  Patient 13 – Clotrimazole prescribed |
| Oedema | 10 | 3 | 2 | 9 | Patient 3 – nonevident, plan to review beta blocker  Patient 22 – Nacsys prescription (possibly reducing the need for corticosteroids)  Patient 24 – reduced Gabapentin |
| Sweating excessive | 5 | 3 | 0 | 2 | Patient 7 – vitamin D3 prescription, stopped opioids  Patient 16 – de-prescribing of Duloxetine  Patient 26 – none |
| Acne/hirsutism/herpes simplex | 3 | 1 | 0 | 2 | Patient 5 (405) – possible link to initiation of treatment for prostate cancer |
| Broken skin/poor healing | 5 | 3 | 0 | 2 | Patient 12 (606) – advice regarding use of creams  Patient 13 (607) – Clotrimazole cream prescribed  Patient 25 (812) – heart failure management |
| Injection site problems | 1 | 1 | 0 | 0 | Patient 13 – advice to rotate injection sites |
| Behaviour problems | 1 | 1 | 0 | 0 | Patient 14 (608) – Gabapentin dose increased, Mirtazapine prescribed |
| Aggression/irritability | 5 | 2 | 3 | 6 | Patient 8 (410) – pain management  Patient 14 (608) - Gabapentine dose increased, Mirtazapine prescribed |
| Agitation, anxiety | 10 | 3 | 1 | 8 | Patient 8 – none (participant refused medicine changes)  Patient 14 – started on Mirtazapine  Patient 27 – Citalopram prescription |
| Panic attacks | 3 | 3 | 0 | 0 | Patient 14 – Mirtazapine prescription  Patient 26 - none  Patient 27 – Citalopram prescription |
| Mood fluctuations | 9 | 0 | 1 | 10 | No improvement |
| Fatigue | 14 | 5 | 4 | 13 | Patient 9 – advice on emollients may have improved sleep  Patient 17 – new anaemia diagnosed, Sytron prescription  Patient 19 – creams to treat itching may have improved sleep  Patient 24 – Gabapentin and Levothyroxine dose decrease, Chlorphenamine de-prescribing  Patient 25 – Bisoprolol de-prescribing |
| Hallucinations, vivid dreams | 8 | 6 | 0 | 2 | Patient 2 – none evident  Patient 5 – Flecainide stopped, improving heart failure, improving dreams (Horie et al., 2021)  Patient 7 – opioid stopped  Patient 16 – Duloxetine de-prescribing  Patient 20 – salbutamol withdrawal  Patient 23 – stopped alpha blocker (less hypotension may stop nightmares) |
| Sleep problems | 9 | 6 | 1 | 4 | Patient 2 – none evident  Patient 6 – possibly due to pain relief or stopped metformin, so no diarrhoea / GI upsets  Patient 13 – stopped opioids, started a cream for itching  Patient 14 – started on Mirtazapine, helps sleep / sedates  Patient 16 – Duloxetine de-prescribing  Patient 23 – as above, de-prescribing of alpha-blocker |
| Sedation | 2 | 2 | 1 | 1 | Patient 7 (407) – stopped Zapain®  Patient 10 (602) – none |
| Dizziness | 9 | 4 | 2 | 7 | Patient 11 – none  Patient 12 – none  Patient 13 – co-codamol de-prescribing  Patient 19 – none |
| Falls in past month | 8 | 4 | 0 | 4 | Patient 11 – none  Patient 14 – attended physiotherapy sessions  Patient 16 – Duloxetine de-prescribing  Patient 19 – home visit, advice given with vital signs measurement |
| Headaches | 6 | 4 | 0 | 2 | Patient 16 – Duloxetine de-prescribing  Patient 17 – Zapain prescription  Patient 20 – Zapain prescription  Patient 24 – stopped chlorphenamine, reduced gabapentin |
| Pain | 24 | 9 | 1 | 16 | Patient 1 – amlodipine stopped (likely leading to reduced swelling and indigestion)  Patient 4 – stopped carbamazepine (less headache & neuropathy)  Patient 5 – new cancer treatment, flecainide stopped (was causing oedema & pain)  Patient 6 – Oromorph and Co-codamol prescriptions  Patient 9 – GP review, advice given  Patient 15 – none  Patient 17 – Zapain prescription  Patient 18 – none  Patient 19 – cream and ointment prescription |
| Tingling, pins, and needles | 11 | 3 | 1 | 9 | Patient 6 – Cyanocobalamin prescription  Patient 19 – ointments prescribed  Patient 22 – diabetic clinic (improved control of diabetes) |
| Hearing problems | 13 | 4 | 1 | 10 | Patient 3 – ear wax removed  Patient 8 - none  Patient 10 – ear wax removed  Patient 19 – none |
| Dry eyes | 9 | 4 | 2 | 7 | Patient 9 – advised to visit opticians  Patient 10 – eye drops already prescribed, none  Patient 14 – eye drops already prescribed; advice given  Patient 18 – advised to visit opticians |
| Urination problems | 7 | 2 | 1 | 6 | Patient 10 – escalated for GP review, given pelvic floor exercises and fluids advice  Patient 20 – lactulose prescription (likely improved constipation, and therefore continence) |
| Chest pain | 4 | 1 | 0 | 3 | Patient 20 – muscular pain, Zapain® prescription |
| SOB | 17 | 3 | 2 | 16 | Patient 1 – stopped amlodipine  Patient 3 – none  Patient 23 – stopped doxazosin |
| High salt intake | 3 | 1 | 0 | 2 | Patient 23 (810) – advice from nurse and pharmacist |
| Dry mouth | 12 | 0 | 1 | 13 | No improvement |
| Hypersalivation | 5 | 2 | 0 | 3 | Patient 13 (607) - none  Patient 18 (801) - none |
| Swallowing difficulties | 4 | 1 | 0 | 3 | Patient 16 – reduced duloxetine (reduces muscle co-ordination) |
| Indigestion/heartburn | 9 | 0 | 1 | 10 | No improvement |
| Bowel problems/  Diarrhoea | 7 | 4 | 1 | 4 | Patient 3 – none, beta blocker and thyroid reviews requested  Patient 6 – metformin stopped  Patient 7 – stopped opioid, reduced constipation & any overflow  Patient 13 – stopped opioid, reduced constipation & any overflow |
| Constipation | 4 | 2 | 1 | 3 | Patient 10 – none  Patient 14 – (takes Zomorph), laxative already prescribed, advice provided |

*Notes to table*

*No change: feet shuffling, skin abnormal, restlessness, confusion, dental problems, appetite, respiratory problems*

*No reports of: self-harm, violence, seizures, halitosis, emesis, sore throat, tongue tremor, catheter use*

*Hair loss was reported as a clinical sign, rather than a problem* per se. *Eyesight problems were not included in this assessment, as they usually indicated a need for new glasses.*

*Questions on pregnancy, the reproductive system, and pain behaviours were omitted.*

## Table S3. GLMMs with all parameters reported

### S3 Table: Changes between baseline and follow-up, as documented in medical records

|  | **Intervention arm (n=27) n (%)** | **Control arm (n=30) n (%)** | **OR (95% CI) unadjusted** | **Trial arm ***  Fixed coefficient (B, exponential)  (95% CI)/ F(df) P | **Significant covariates**  Fixed coefficient (95% CI) P | Overall model significance F(df) P | Model fit  AIC, BIC | ICC,  Adjusted/  conditional | Pseudo R^2^ conditional |  |  |  |  |  |
| --- | --- | --- | --- | --- | --- | --- | --- | --- | --- | --- | --- | --- | --- | --- |
| Problems documented in medical records | | | | | |  |  |  |  |  |  |  |  |  |
| >1 problem addressed | 22 (82) | 11 (36.7) | 7.60 (2.24-25.81) | 7.48 (1.99-28.10)/ 9.30 (1,52) 0.004 | None, only trial arm | 2.76(4,52) 0.04 | 261.59. 265.28 | 0.47/0.36 | 0.51 |  |  |  |  |  |
| At least 1 more problem recorded | 19 (70) | 23 (77) | 0.72 (0.22-2.36) | 1.41 (0.21-9.32) / 0.14 (1,52) 0.72 | SES, wealthier people had fewer new problems: 0.15 (0.03-0.72) /5.87 (1,52) 0.02 | 1.70 (4,52) 0.16 | 274.81  278.47 | 0.12/0.45 | 0.45 |  |  |  |  |  |
| Prescribing | | | | | |  |  |  |  |  |  |  |  |  |
| Deprescribing, medicines discontinued | 15 (56) | 9 (30) | 2.92 (0.98, 8.67) | 2.37 (0.70-8.07)/ 2.01 (1,52) 0.16 | SES, wealthier people had more deprescribing: 4.53 (1.19-17.35)/5.09 (1,52) 0.03 | 2.08 (4,52) 0.10 | 257.90, 261.56 | 0.44/ 0.39 | 0.50 |  |  |  |  |  |
| Describing, medicines stopped or doses reduced | 18 (64) | 11 (37) | 3.46 (1.16-10.29) | 2.83 (0.70-11.44)/ 2.27 (1,52) 0.14 | None, trial arm strongest association | 1.54 (4,52) 0.21 | 252.62, 256.28 | 0.48/ 0.43 | 0.53 |  |  |  |  |  |
| Any new prescriptions | 16 (59) | 23 (77) | 0.44 (0.14-1.39) | 0.39 (0.11-1.43)/ 2.14 (1,52) 0.15 | None, trial arm strongest association | 1.39 (4,52) 0.25 | 269.47/ 273.12 | 0.40/ 0.37 | 0.45 |  |  |  |  |  |

*Notes to table. OR odds ratio, aOR adjusted odds ratio, CI confidence intervals, ICC intraclass correlation coefficient, SES socio-economic status, SE standard error, AIC* Akaike Information Criterion, BIC Bayesian Information Criterion

** General Linear Mixed Models (GLMM) with GP practice as the random intercept and adjusted for deprivation (most deprived WIMD quintile vs quintiles 2-5), age (<76), and number of long-term conditions. Probability distribution was binomial, and link function logit. There are no missing data.*

*Conditional pseudo R^2^ reported, to account for fixed and random effects.*

## Appendix to Table 5: Parameters of Regression Models

GP practice was irrevocably linked to trial arm.

Numbers of long-term conditions and prescriptions were closely linked, and only the number of conditions was entered into the model to minimise collinearity. Data on age and deprivation were dichotomized: participants aged 65-75 (reference group) were compared with older participants; those in the most deprived quintile (reference group) were compared with patients in all other quintiles to reflect the study population. ‘Number of long-term conditions’ was entered as a continuous variable; this variable was normally distributed.

WIMD, Welsh index of multiple deprivation

## Problems addressed

In adjusted analyses, the only statistically significant predictor of >1 problem addressed was the use of ADRe.

### Table S5a Factors affecting >1 problem being addressed: adjusted analysis, GLMM

| **Fixed Coefficients^a^** | | | | | | | | | |
| --- | --- | --- | --- | --- | --- | --- | --- | --- | --- |
| Model Term | Coefficient | Std. Error | t | Sig. | 95% Confidence Interval | | Exp(Coefficient) | 95% Confidence Interval for Exp(Coefficient) | |
|  |  |  |  |  | Lower | Upper |  | Lower | Upper |
| Intercept | -1.692 | 2.0387 | -.830 | .410 | -5.783 | 2.399 | .184 | .003 | 11.008 |
| Allocation=0 | 2.012 | .6596 | 3.050 | .004 | .688 | 3.336 | 7.478 | 1.990 | 28.095 |
| Allocation=1 | 0^b^ | . | . | . | . | . | . | . | . |
| WIMD_groups=1 | .624 | .7175 | .869 | .389 | -.816 | 2.063 | 1.866 | .442 | 7.871 |
| WIMD_groups=2 | 0^b^ | . | . | . | . | . | . | . | . |
| Age_group=0 | .240 | .6682 | .359 | .721 | -1.101 | 1.580 | 1.271 | .333 | 4.857 |
| Age_group=1 | 0^b^ | . | . | . | . | . | . | . | . |
| Chronic_conditions | -.098 | .2177 | -.450 | .655 | -.535 | .339 | .907 | .586 | 1.403 |
| Probability distribution: Binomial. Link function: Logit^a^ | | | | | | | | | |
| a. Target: 2 or more problems addressed | | | | | | | | | |
| b. This coefficient is set to zero because it is redundant. | | | | | | | | | |

## Table S5b. Problems recorded in the medical records

We identified participants where at least one more symptom was newly recorded in the medical records in the six months between the two rounds of record review. More new symptoms appeared amongst control arm participants. We speculate that without ADRe, symptoms were not being addressed until they became sufficiently serious to warrant visiting the GP.

### Table S5b. At least one more problem recorded GLMM

| **Fixed Coefficients^a^** | | | | | | | | | |
| --- | --- | --- | --- | --- | --- | --- | --- | --- | --- |
| Model Term | Coefficient | Std. Error | t | Sig. | 95% Confidence Interval | | Exp(Coefficient) | 95% Confidence Interval for Exp(Coefficient) | |
|  |  |  |  |  | Lower | Upper |  | Lower | Upper |
| Intercept | -2.130 | 2.0430 | -1.043 | .302 | -6.230 | 1.970 | .119 | .002 | 7.169 |
| Allocation=0 | .346 | .9401 | .368 | .715 | -1.541 | 2.232 | 1.413 | .214 | 9.320 |
| Allocation=1 | 0^b^ | . | . | . | . | . | . | . | . |
| WIMD_groups=1 | -1.929 | .7963 | -2.422 | .019 | -3.527 | -.331 | .145 | .029 | .718 |
| WIMD_groups=2 | 0^b^ | . | . | . | . | . | . | . | . |
| Age_group=0 | 1.314 | .8248 | 1.593 | .117 | -.341 | 2.969 | 3.722 | .711 | 19.479 |
| Age_group=1 | 0^b^ | . | . | . | . | . | . | . | . |
| Chronic_conditions | .329 | .2591 | 1.270 | .210 | -.191 | .849 | 1.390 | .826 | 2.337 |
| Probability distribution: Binomial. Link function: Logit^a^ | | | | | | | | | |
| a. Target: At least one more sign or symptom of a potential ADR (=problem) at Step 2, compared to Step 1, in medical notes | | | | | | | | | |
| b. This coefficient is set to zero because it is redundant. | | | | | | | | | |

## Deprescribing: medicines discontinued

Deprescribing was more frequent amongst the more affluent patients. Relative affluence was a stronger predictor of de-prescribing than use of ADRe, when analysed using logistic regression (enter and backwards LR models).

### Table S5c. Deprescribing adjusted analysis: GLMM

| **Fixed Coefficients^a^** | | | | | | | | | |
| --- | --- | --- | --- | --- | --- | --- | --- | --- | --- |
| Model Term | Coefficient | Std. Error | t | Sig. | 95% Confidence Interval | | Exp(Coefficient) | 95% Confidence Interval for Exp(Coefficient) | |
|  |  |  |  |  | Lower | Upper |  | Lower | Upper |
| Intercept | -1.582 | 2.0872 | -.758 | .452 | -5.770 | 2.606 | .206 | .003 | 13.550 |
| Allocation=0 | .864 | .6100 | 1.417 | .162 | -.360 | 2.088 | 2.373 | .698 | 8.072 |
| Allocation=1 | 0^b^ | . | . | . | . | . | . | . | . |
| WIMD_groups=1 | 1.510 | .6695 | 2.256 | .028 | .167 | 2.854 | 4.528 | 1.182 | 17.352 |
| WIMD_groups=2 | 0^b^ | . | . | . | . | . | . | . | . |
| Age_group=0 | -.437 | .6564 | -.665 | .509 | -1.754 | .880 | .646 | .173 | 2.412 |
| Age_group=1 | 0^b^ | . | . | . | . | . | . | . | . |
| Chronic_conditions | .192 | .2209 | .871 | .388 | -.251 | .636 | 1.212 | .778 | 1.888 |
| Probability distribution: Binomial. Link function: Logit^a^ | | | | | | | | | |
| a. Target: Any de-prescribing between Step 1 and Step 2? | | | | | | | | | |
| b. This coefficient is set to zero because it is redundant. | | | | | | | | | |

## Any deprescribing: medicines stopped or doses reduced

Any deprescribing was taken as discontinuation or dose reduction. This was more prevalent in the intervention arm.

### Table S5d. Medicines discontinued or reduced: GLMM

| **Fixed Coefficients^a^** | | | | | | | | | |
| --- | --- | --- | --- | --- | --- | --- | --- | --- | --- |
| Model Term | Coefficient | Std. Error | t | Sig. | 95% Confidence Interval | | Exp(Coefficient) | 95% Confidence Interval for Exp(Coefficient) | |
|  |  |  |  |  | Lower | Upper |  | Lower | Upper |
| Intercept | -1.338 | 2.1294 | -.628 | .532 | -5.611 | 2.935 | .262 | .004 | 18.815 |
| Allocation=0 | 1.039 | .6966 | 1.492 | .142 | -.358 | 2.437 | 2.827 | .699 | 11.440 |
| Allocation=1 | 0^b^ | . | . | . | . | . | . | . | . |
| WIMD_groups=1 | .714 | .6709 | 1.064 | .292 | -.632 | 2.060 | 2.042 | .531 | 7.848 |
| WIMD_groups=2 | 0^b^ | . | . | . | . | . | . | . | . |
| Age_group=0 | -.935 | .6424 | -1.455 | .152 | -2.224 | .354 | .393 | .108 | 1.425 |
| Age_group=1 | 0^b^ | . | . | . | . | . | . | . | . |
| Chronic_conditions | .218 | .2111 | 1.033 | .306 | -.206 | .642 | 1.244 | .814 | 1.900 |
| Probability distribution: Binomial. Link function: Logit^a^ | | | | | | | | | |
| a. Target: deprescribing inc lower dose binary | | | | | | | | | |
| b. This coefficient is set to zero because it is redundant. | | | | | | | | | |

## New Prescriptions

New prescriptions were more prevalent in the control arm, but the difference was statistically non-significant. New prescribing occurred at a similar rate in both deprivation categories (25/40, 63% vs 14/18, 78%). Adjusted analysis did not identify any associations.

### Table S5e. Factors affecting new prescribing (any) enter model

| **Fixed Coefficients^a^** | | | | | | | | | |
| --- | --- | --- | --- | --- | --- | --- | --- | --- | --- |
| Model Term | Coefficient | Std. Error | t | Sig. | 95% Confidence Interval | | Exp(Coefficient) | 95% Confidence Interval for Exp(Coefficient) | |
|  |  |  |  |  | Lower | Upper |  | Lower | Upper |
| Intercept | -.125 | 2.0583 | -.061 | .952 | -4.256 | 4.005 | .882 | .014 | 54.873 |
| Allocation=0 | -.932 | .6368 | -1.463 | .149 | -2.210 | .346 | .394 | .110 | 1.413 |
| Allocation=1 | 0^b^ | . | . | . | . | . | . | . | . |
| WIMD_groups=1 | .838 | .7196 | 1.164 | .250 | -.606 | 2.282 | 2.311 | .545 | 9.793 |
| WIMD_groups=2 | 0^b^ | . | . | . | . | . | . | . | . |
| Age_group=0 | .570 | .6627 | .860 | .394 | -.760 | 1.900 | 1.768 | .468 | 6.685 |
| Age_group=1 | 0^b^ | . | . | . | . | . | . | . | . |
| Chronic_conditions | -.317 | .2329 | -1.363 | .179 | -.785 | .150 | .728 | .456 | 1.162 |
| Probability distribution: Binomial. Link function: Logit^a^ | | | | | | | | | |
| a. Target: Any new prescriptions between Step 1 and Step 2? (including creams and vitamins). | | | | | | | | | |
| b. This coefficient is set to zero because it is redundant. | | | | | | | | | |
