# Supplementary material for: “A better system” – managing the adverse effects of prescribed medicines: Cluster-randomised controlled trial and process evaluation of the ADRe Profile in primary care
Source: PLoS One. 2026 Sep 18;21(9):e0356480. doi: 10.1371/journal.pone.0356480 (PMC13588343; doi:10.1371/journal.pone.0356480)
Supplement: Supplementary file 5 [file pone.0356480.s005.docx]

## Folder description

Survey responses

This folder contains a dataset of collected responses from participants who completed the ADRe questionnaire. The files capture participants’ answers to closed- and open-ended questions, along with associated variables such as demographic indicators. The contents are organised to support mixed-methods analysis.

Cognitive interviews

The folder contains cognitive interview analysis conducted with different participant groups - a GP, a pharmacist, a nurse and two patients. Each document captures structured insights, including interview transcripts, thematic coding, and interpretive summaries tailored to each subgroup. The two profession-specific analyses focus on general practitioners and pharmacists.

Interview transcripts

This folder contains a collection of qualitative data files documenting individual interviews conducted with pharmacists as part of the study. Each file represents a verbatim transcript of a single interview, capturing participants’ responses, perspectives, and experiences relevant to the research topic.

The folder of transcripts is available by application to Swansea University library. The non-author contact is: [library-digital@swansea.ac.uk](mailto:library-digital@swansea.ac.uk) The author contact is [s.e.jordan@swansea.ac.uk](mailto:s.e.jordan@swansea.ac.uk) .

URL is: [https://zenodo.org/records/19736675](https://eur03.safelinks.protection.outlook.com/?url=https%3A%2F%2Fzenodo.org%2Frecords%2F19736675&data=05%7C02%7CS.E.Jordan%40Swansea.ac.uk%7C83aeda658df546be630708dea213df7c%7Cbbcab52e9fbe43d6a2f39f66c43df268%7C0%7C0%7C639126403384132438%7CUnknown%7CTWFpbGZsb3d8eyJFbXB0eU1hcGkiOnRydWUsIlYiOiIwLjAuMDAwMCIsIlAiOiJXaW4zMiIsIkFOIjoiTWFpbCIsIldUIjoyfQ%3D%3D%7C0%7C%7C%7C&sdata=SQHlCHq4mjoPeWvQltF78DLlqRiwaYFsjOgm0F%2BzhCk%3D&reserved=0)

Doi is: 10.5281/zenodo.19736674 ([https://doi.org/10.5281/zenodo.19736674](https://eur03.safelinks.protection.outlook.com/?url=https%3A%2F%2Fdoi.org%2F10.5281%2Fzenodo.19736674&data=05%7C02%7CS.E.Jordan%40Swansea.ac.uk%7C83aeda658df546be630708dea213df7c%7Cbbcab52e9fbe43d6a2f39f66c43df268%7C0%7C0%7C639126403384153949%7CUnknown%7CTWFpbGZsb3d8eyJFbXB0eU1hcGkiOnRydWUsIlYiOiIwLjAuMDAwMCIsIlAiOiJXaW4zMiIsIkFOIjoiTWFpbCIsIldUIjoyfQ%3D%3D%7C0%7C%7C%7C&sdata=f%2Bug4JV6U367VhtwegaM7lf%2Fo9v7UqOfnk5HwnJu1f8%3D&reserved=0))
