# Supplementary material for: “A better system” – managing the adverse effects of prescribed medicines: Cluster-randomised controlled trial and process evaluation of the ADRe Profile in primary care
Source: PLoS One. 2026 Sep 18;21(9):e0356480. doi: 10.1371/journal.pone.0356480 (PMC13588343; doi:10.1371/journal.pone.0356480)
Supplement: Supplementary file 6 [file pone.0356480.s007.docx]

## Response to reviewers and editor

27.4.26

Dear Dr. Gebreyohannes,

# “A better system” – managing the adverse effects of prescribed medicines: cluster-randomised controlled trial and process evaluation of the ADRe Profile in primary care

Short title: Monitoring patients for adverse effects of prescribed medicines

PONE-D-26-02770

Thank you for the opportunity to respond to the reviewers’ helpful comments. We have re-analysed the data and clarified as requested. Reponses to individual comments are tabulated.

We are grateful to the reviewers for their insightful comments.

While the reviewers recognise the potential relevance of your study, they have raised several important concerns that need to be addressed before the manuscript can be considered further.

In particular, the reviewers highlighted the following key issues among others:

| - Academic writing and clarity: The manuscript would benefit from substantial editing to improve the clarity, structure, and overall academic writing. | We apologise for any lack of clarity. The manuscript has been re-edited by native English speakers, who are editors of research journals. We are unable to identify either statements that need further support or grammatical errors. |
| --- | --- |
| - Primary outcome: The primary outcome of the study should be explicitly stated and consistently presented. | We apologise for any lack of clarity. We have re-written (line 143 *et seq*):  The primary outcome was ‘health problems addressed’, as indicated by the medical records. ‘Health problems’ were defined as signs and symptoms listed on ADRe [46] [44 p.5], and S3 file. ‘Problems addressed’ were defined as those where action was taken following problem identification, for example by changes in prescribing or referrals or health promotion [44 p.5] (examples are described under ‘clinical gain’ and in S4 file, S2 Table). Before and after ADRe use, in both arms, using medical records, we assessed the number and nature of health problems identified and addressed and changes in prescribing, including deprescribing (see glossary), dose reduction, new prescriptions, laboratory tests, and recording of vital signs [55]. |
| - Power calculation and clustering | Thank you. We have moved the detail of the calculation from the published protocol [44], from supplementary material into the manuscript (line 163 *et seq*).  In a ‘before and after’ single arm intervention study, with participants acting as their own controls, 17/19 (89%) had at least one clinical problem addressed when ADRe for polypharmacy was used [8]. We hypothesised that 9/19 (47%) would have shown some improvement without intervention, necessitating an effective sample of 40 patients (20 in each arm), with 5% significance and 90% power [56]. Based on the reported intra-cluster coefficient (0.02), [43], and a design effect of 1.18, to achieve an effective sample size of 40, an actual sample size of 48 was needed (Killip et al 2004) [58]. Ten patients in each of 6 clusters (GP practices) allows for 20% loss to follow up (S3 file).  Thank you. The two uses of the term ‘clustering’ have been explicated. We apologise for any confusion.  We have two distinct types of “clusters”. To avoid ambiguity. GP clusters have now been re-named GP groups, retaining the term ‘cluster’ solely as a statistical concept. |
| - Handling of missing data | - Data collection from **medical notes** was complete (Tables 4, 5). - We have detailed where missing data arose. We explain (line 219 *et seq*):   Missing data arose in the ADRe questionnaire when: a) participants completed ADRe by telephone or were unable to stand it was impossible to obtain measurements of all vital signs (5/27 respondents); b) one questions were designated as ‘discretionary’ due to cultural sensitivities: the question on sexual dysfunction was omitted by 15/27 respondents; all other questions were complete. These data were not subjected to inferential analyses. (Further details are in S3 file.) Differences between those responding and not responding militated against transferability of responses, and hence data imputation.      Reasons for missing data   - Lack of equipment (e.g. scales, device battery) - Participant physical limitations (e.g., inability to stand to measure standing blood pressure) - Telephone-based completion - Clinical discretion for sensitive items |
| - The manuscript should clearly explain any deviations from the originally planned study design and discuss their potential implications. | These were detailed in S3 file, and more details have now been added to the manuscript.  Protocol deviations are detailed in S3 file; most notably, time between the data collection rounds was more protracted than planned for 22/27 participants.   - Delays in follow-up timing (planned: 4-12 weeks), actual: 16 had follow-up at 17-20 weeks (causes: logistical delays |

Please carefully revise your manuscript in light of the reviewers’ comments and provide a detailed, point-by-point response to each comment.
==============================

Please submit your revised manuscript by Apr 29 2026 11:59PM. If you will need more time than this to complete your revisions, please reply to this message or contact the journal office at [plosone@plos.org](mailto:plosone@plos.org). When you're ready to submit your revision, log on to <https://www.editorialmanager.com/pone/> and select the 'Submissions Needing Revision' folder to locate your manuscript file.

Please include the following items when submitting your revised manuscript:

- A letter that responds to each point raised by the academic editor and reviewer(s). You should upload this letter as a separate file labeled 'Response to Reviewers'.
- A marked-up copy of your manuscript that highlights changes made to the original version. You should upload this as a separate file labeled 'Revised Manuscript with Track Changes'.
- An unmarked version of your revised paper without tracked changes. You should upload this as a separate file labeled 'Manuscript'.

If you would like to make changes to your financial disclosure, please include your updated statement in your cover letter. Guidelines for resubmitting your figure files are available below the reviewer comments at the end of this letter.

If applicable, we recommend that you deposit your laboratory protocols in protocols.io to enhance the reproducibility of your results. Protocols.io assigns your protocol its own identifier (DOI) so that it can be cited independently in the future. For instructions see: [https://journals.plos.org/plosone/s/submission-guidelines#loc-laboratory-protocols](https://eur03.safelinks.protection.outlook.com/?url=https%3A%2F%2Ftrack.editorialmanager.com%2FCL0%2Fhttps%3A%252F%252Fjournals.plos.org%252Fplosone%252Fs%252Fsubmission-guidelines%2523loc-laboratory-protocols%2F1%2F010f019cf46d4d9c-cb5c07c8-c29f-4c92-ba65-709f92492ce2-000000%2F4hWhd2XRJxV_D4u2RlCJfZb2R04h3GoeHPFLOKcXtmc%3D254&data=05%7C02%7Cs.e.jordan%40swansea.ac.uk%7C6f20e634672c4a1012b208de8301e62f%7Cbbcab52e9fbe43d6a2f39f66c43df268%7C0%7C0%7C639092241630050387%7CUnknown%7CTWFpbGZsb3d8eyJFbXB0eU1hcGkiOnRydWUsIlYiOiIwLjAuMDAwMCIsIlAiOiJXaW4zMiIsIkFOIjoiTWFpbCIsIldUIjoyfQ%3D%3D%7C0%7C%7C%7C&sdata=mTCXTw4ihCyY62%2B8KGp3vCtkyKeZ1Jc2GySN2Qqog3o%3D&reserved=0). Additionally, PLOS ONE offers an option for publishing peer-reviewed Lab Protocol articles, which describe protocols hosted on protocols.io. Read more information on sharing protocols at [https://plos.org/protocols?utm_medium=editorial-email&utm_source=authorletters&utm_campaign=protocols](https://eur03.safelinks.protection.outlook.com/?url=https%3A%2F%2Ftrack.editorialmanager.com%2FCL0%2Fhttps%3A%252F%252Fplos.org%252Fprotocols%253Futm_medium%3Deditorial-email%2526utm_source%3Dauthorletters%2526utm_campaign%3Dprotocols%2F1%2F010f019cf46d4d9c-cb5c07c8-c29f-4c92-ba65-709f92492ce2-000000%2F92Cl17YMJ3LB16cqQ3oPwc3QFhYJ-tl4iHPUff8nmN8%3D254&data=05%7C02%7Cs.e.jordan%40swansea.ac.uk%7C6f20e634672c4a1012b208de8301e62f%7Cbbcab52e9fbe43d6a2f39f66c43df268%7C0%7C0%7C639092241630071862%7CUnknown%7CTWFpbGZsb3d8eyJFbXB0eU1hcGkiOnRydWUsIlYiOiIwLjAuMDAwMCIsIlAiOiJXaW4zMiIsIkFOIjoiTWFpbCIsIldUIjoyfQ%3D%3D%7C0%7C%7C%7C&sdata=Dy4HDpNOWIWgPmDeX0anz8CISmKqbulPH5V7bw%2Fza4w%3D&reserved=0).

We look forward to receiving your revised manuscript.

Kind regards,

Eyob Alemayehu Gebreyohannes, PhD
Academic Editor
PLOS One

Dear Dr. Gebreyohannes, and Team,

Thank you

Journal Requirements:

When submitting your revision, we need you to address these additional requirements.

1. Please ensure that your manuscript meets PLOS ONE's style requirements, including those for file naming. The PLOS ONE style templates can be found at

<https://journals.plos.org/plosone/s/file?id=wjVg/PLOSOne_formatting_sample_main_body.pdf> and

<https://journals.plos.org/plosone/s/file?id=ba62/PLOSOne_formatting_sample_title_authors_affiliations.pdf>

***We have endeavoured to comply with all requirements. Please could you identify any outstanding issues.***

2. Thank you for stating the following in the Acknowledgments Section of your manuscript:

“This work was supported by Swansea University (Centenary Research Excellence Scholarship), sponsor reference RIO 034-20. The funders had no role in study design, data collection and analysis, interpretation of data, decision to publish, or preparation of the manuscript.”

We note that you have provided funding information that is not currently declared in your Funding Statement. However, funding information should not appear in the Acknowledgments section or other areas of your manuscript. We will only publish funding information present in the Funding Statement section of the online submission form.

Please remove any funding-related text from the manuscript and let us know how you would like to update your Funding Statement. Currently, your Funding Statement reads as follows:

“This work was supported by Swansea University (Centenary Research Excellence Scholarship), sponsor reference RIO 034-20. The funders had no role in study design, data collection and analysis, interpretation of data, decision to publish, or preparation of the manuscript.”

Please include your amended statements within your cover letter; we will change the online submission form on your behalf.

***Thank you. This has been removed from manuscript and included in the covering letter, as advised.***

3. Thank you for stating the following financial disclosure:

“This work was supported by Swansea University (Centenary Research Excellence Scholarship), sponsor reference RIO 034-20. The funders had no role in study design, data collection and analysis, interpretation of data, decision to publish, or preparation of the manuscript.”

We note that one or more of the authors is affiliated with the funding organization, indicating the funder may have had some role in the design, data collection, analysis or preparation of your manuscript for publication; in other words, the funder played an indirect role through the participation of the co-authors. If the funding organization did not play a role in the study design, data collection and analysis, decision to publish, or preparation of the manuscript and only provided financial support in the form of authors' salaries and/or research materials, please do the following:

1. Review your statements relating to the author contributions, and ensure you have specifically and accurately indicated the role(s) that these authors had in your study. These amendments should be made in the online form.

***We are confident that these are accurate, but please forward any specific questions you may have.***

2. Confirm in your cover letter that you agree with the following statement, and we will change the online submission form on your behalf:

“The funder provided support in the form of salaries for authors [insert relevant initials], but did not have any additional role in the study design, data collection and analysis, decision to publish, or preparation of the manuscript. The specific roles of these authors are articulated in the ‘author contributions’ section.

***Yes, thank you, please do this for us. We have added this to the covering letter, as advised.***

4. Your ethics statement should only appear in the Methods section of your manuscript. If your ethics statement is written in any section besides the Methods, please delete it from any other section.

***This has been done.***

5. Please ensure that you refer to Figure 2 in your text as, if accepted, production will need this reference to link the reader to the figure.

***We apologise for this oversight. It has been corrected.***

6. We note that there is identifying data in the Supporting Information file “Supplementary material 4 additional tables.docx”. Due to the inclusion of these potentially identifying data, we have removed this file from your file inventory. Prior to sharing human research participant data, authors should consult with an ethics committee to ensure data are shared in accordance with participant consent and all applicable local laws.

Data sharing should never compromise participant privacy. It is therefore not appropriate to publicly share personally identifiable data on human research participants. The following are examples of data that should not be shared:

-Name, initials, physical address
-Ages more specific than whole numbers
-Internet protocol (IP) address
-Specific dates (birth dates, death dates, examination dates, etc.)
-Contact information such as phone number or email address
-Location data
-ID numbers that seem specific (long numbers, include initials, titled “Hospital ID”) rather than random (small numbers in numerical order)

Data that are not directly identifying may also be inappropriate to share, as in combination they can become identifying. For example, data collected from a small group of participants, vulnerable populations, or private groups should not be shared if they involve indirect identifiers (such as sex, ethnicity, location, etc.) that may risk the identification of study participants.

Additional guidance on preparing raw data for publication can be found in our Data Policy (<https://journals.plos.org/plosone/s/data-availability#loc-human-research-participant-data-and-other-sensitive-data>) and in the following article: <http://www.bmj.com/content/340/bmj.c181.long>.

Please remove or anonymize all personal information “ID numbers”, ensure that the data shared are in accordance with participant consent, and re-upload a fully anonymized data set. Please note that spreadsheet columns with personal information must be removed and not hidden as all hidden columns will appear in the published file.

**Following this advice, we have removed the column with ID numbers and demographic information in S4 file (table S1) and replaced multi-digit ID numbers with a simplified sequence (1-27) in table 2 and throughout the manuscript.**

7. We note that this data set consists of interview transcripts. Can you please confirm that all participants gave consent for interview transcript to be published?

**Point 4 of the consent form states: ‘Agree to the ADRe Profile consultation with the nurse/researcher to be audio recorded and for the anonymised data and quotes to be used in the thesis and relevant professional publications.’**

**The transcripts do not contain any potentially identifying information.**

If they DID provide consent for these transcripts to be published, please also confirm that the transcripts do not contain any potentially identifying information (or let us know if the participants consented to having their personal details published and made publicly available). We consider the following details to be identifying information:

- Names, nicknames, and initials
- Age more specific than round numbers
- GPS coordinates, physical addresses, IP addresses, email addresses
- Information in small sample sizes (e.g. 40 students from X class in X year at X university)
- Specific dates (e.g. visit dates, interview dates)
- ID numbers

Or, if the participants DID NOT provide consent for these transcripts to be published:

- Provide a de-identified version of the data or excerpts of interview responses

- Provide information regarding how these transcripts can be accessed by researchers who meet the criteria for access to confidential data, including:

a) the grounds for restriction

**We did not seek consent to place transcripts in the public domain. The wording of the signed consent (above) does not specifically allow full transcripts to be published.**

b) the name of the ethics committee, Institutional Review Board, or third-party organization that is imposing sharing restrictions on the data

**Health Research Authority Research Ethics Committee (Wales REC 6) 17th March 2021 (Integrated Research Application System [IRAS] 292693.**

**All participants had full capacity.**

c) a non-author, institutional point of contact that is able to field data access queries, in the interest of maintaining long-term data accessibility.
**We have deposited the transcripts with Swansea University library. The non-author contact is:** [**library-digital@swansea.ac.uk**](mailto:library-digital@swansea.ac.uk)**. The author contact is** [**s.e.jordan@swansea.ac.uk**](mailto:s.e.jordan@swansea.ac.uk)**.**

**URL is:** [**https://zenodo.org/records/19736675**](https://eur03.safelinks.protection.outlook.com/?url=https%3A%2F%2Fzenodo.org%2Frecords%2F19736675&data=05%7C02%7CS.E.Jordan%40Swansea.ac.uk%7C83aeda658df546be630708dea213df7c%7Cbbcab52e9fbe43d6a2f39f66c43df268%7C0%7C0%7C639126403384132438%7CUnknown%7CTWFpbGZsb3d8eyJFbXB0eU1hcGkiOnRydWUsIlYiOiIwLjAuMDAwMCIsIlAiOiJXaW4zMiIsIkFOIjoiTWFpbCIsIldUIjoyfQ%3D%3D%7C0%7C%7C%7C&sdata=SQHlCHq4mjoPeWvQltF78DLlqRiwaYFsjOgm0F%2BzhCk%3D&reserved=0)

**Doi is: 10.5281/zenodo.19736674 (**[**https://doi.org/10.5281/zenodo.19736674**](https://eur03.safelinks.protection.outlook.com/?url=https%3A%2F%2Fdoi.org%2F10.5281%2Fzenodo.19736674&data=05%7C02%7CS.E.Jordan%40Swansea.ac.uk%7C83aeda658df546be630708dea213df7c%7Cbbcab52e9fbe43d6a2f39f66c43df268%7C0%7C0%7C639126403384153949%7CUnknown%7CTWFpbGZsb3d8eyJFbXB0eU1hcGkiOnRydWUsIlYiOiIwLjAuMDAwMCIsIlAiOiJXaW4zMiIsIkFOIjoiTWFpbCIsIldUIjoyfQ%3D%3D%7C0%7C%7C%7C&sdata=f%2Bug4JV6U367VhtwegaM7lf%2Fo9v7UqOfnk5HwnJu1f8%3D&reserved=0)**)**

d) Any relevant data set names, URLs, DOIs, etc. that an independent researcher would need in order to request your minimal data set.

For further information on sharing data that contains sensitive participant information, please see: <https://journals.plos.org/plosone/s/data-availability#loc-human-research-participant-data-and-other-sensitive-data>
If there are ethical, legal, or third-party restrictions upon your dataset, you must provide all of the following details (<https://journals.plos.org/plosone/s/data-availability#loc-acceptable-data-access-restrictions>):

1. A complete description of the dataset

**This is included in the folder, alongside the transcripts, and as S5 file.**

2. The nature of the restrictions upon the data (ethical, legal, or owned by a third party) and the reasoning behind them

**Ethical. Health Research Authority Research Ethics Committee (Wales REC 6) 17th March 2021 (Integrated Research Application System [IRAS] 292693.**

**The signed consent obtained gave permission for quotations to be used. There was no explicit consent for transcripts to be placed in the public domain.**
3. The full name of the body imposing the restrictions upon your dataset (ethics committee, institution, data access committee, etc)

**We did not seek permission to place full transcripts in the public domain. It is reasonable to make these de-identified transcripts available to *bona fide* researchers on request.**

**Ethical. Health Research Authority Research Ethics Committee (Wales REC 6) 17th March 2021 (Integrated Research Application System [IRAS] 292693.**

4. If the data are owned by a third party, confirmation of whether the authors received any special privileges in accessing the data that other researchers would not have

NA
5. Direct, non-author contact information (preferably email) for the body imposing the restrictions upon the data, to which data access requests can be sent

**We have deposited the transcripts with Swansea University library. The non-author contact is:** [**library-digital@swansea.ac.uk**](mailto:library-digital@swansea.ac.uk)**. The author contact is** [**s.e.jordan@swansea.ac.uk**](mailto:s.e.jordan@swansea.ac.uk)**.**

**URL is:** [**https://zenodo.org/records/19736675**](https://eur03.safelinks.protection.outlook.com/?url=https%3A%2F%2Fzenodo.org%2Frecords%2F19736675&data=05%7C02%7CS.E.Jordan%40Swansea.ac.uk%7C83aeda658df546be630708dea213df7c%7Cbbcab52e9fbe43d6a2f39f66c43df268%7C0%7C0%7C639126403384132438%7CUnknown%7CTWFpbGZsb3d8eyJFbXB0eU1hcGkiOnRydWUsIlYiOiIwLjAuMDAwMCIsIlAiOiJXaW4zMiIsIkFOIjoiTWFpbCIsIldUIjoyfQ%3D%3D%7C0%7C%7C%7C&sdata=SQHlCHq4mjoPeWvQltF78DLlqRiwaYFsjOgm0F%2BzhCk%3D&reserved=0)

**Doi is: 10.5281/zenodo.19736674 (**[**https://doi.org/10.5281/zenodo.19736674**](https://eur03.safelinks.protection.outlook.com/?url=https%3A%2F%2Fdoi.org%2F10.5281%2Fzenodo.19736674&data=05%7C02%7CS.E.Jordan%40Swansea.ac.uk%7C83aeda658df546be630708dea213df7c%7Cbbcab52e9fbe43d6a2f39f66c43df268%7C0%7C0%7C639126403384153949%7CUnknown%7CTWFpbGZsb3d8eyJFbXB0eU1hcGkiOnRydWUsIlYiOiIwLjAuMDAwMCIsIlAiOiJXaW4zMiIsIkFOIjoiTWFpbCIsIldUIjoyfQ%3D%3D%7C0%7C%7C%7C&sdata=f%2Bug4JV6U367VhtwegaM7lf%2Fo9v7UqOfnk5HwnJu1f8%3D&reserved=0)**)**

**The folder includes all transcripts from this study. The responsible ethics committee is: Health Research Authority Research Ethics Committee (Wales REC 6) (Integrated Research Application System [IRAS] 292693.**

8. If the reviewer comments include a recommendation to cite specific previously published works, please review and evaluate these publications to determine whether they are relevant and should be cited. There is no requirement to cite these works unless the editor has indicated otherwise.

NA
[Note: HTML markup is below. Please do not edit.]

5. Review Comments to the Author

| Reviewer #1: As the statistical reviewer I will focus on methods and reporting  Major 1) aspects of the paper are not easy to follow with bullet points and not strong academic writing. | Thank you for this insightful comment. We have revisited the manuscript.  We did not identify any bullet points. The manuscript has been re-edited by three professors, all native English speakers, who are also editors. We did not identify any grammatical errors or unorthodox style or unsupported statements. We have aimed for appropriate intertextuality.  <https://en.wikipedia.org/wiki/Academic_writing> |
| --- | --- |
| 2) it was not clear what the primary outcome is in the relevant section (study outcomes). This is key since all other analyses for other outcomes need to be treated as exploratory since the power calculation does not relate to them. | Thank you. We have clarified (line 143 *et seq*):  A) The primary outcome was ‘health problems addressed’, as indicated by the medical records. ‘Health problems’ were defined as signs and symptoms listed on ADRe [46] [44 p.5], and S3 file. ‘Problems addressed’ were defined as those where action was taken following problem identification, for example by changes in prescribing or referrals or health promotion [44 p.5] (examples are described under ‘clinical gain’ and in S4 file, S2 Table). Before and after ADRe use, in both arms, using medical records, we assessed the number and nature of health problems identified and addressed and changes in prescribing, including deprescribing (see glossary), dose reduction, new prescriptions, laboratory tests, and recording of vital signs [55]. |
| 3) specifically about the power calculation e.g. from previous work. | We have transferred the text from the protocol paper [44] from supplementary material to the manuscript. We have added (line 163 *et seq*):  In a ‘before and after’ single arm intervention study, with participants acting as their own controls, 17/19 (89%) had at least one clinical problem addressed when ADRe for polypharmacy was used [8]. We hypothesised that 9/19 (47%) would have shown some improvement without intervention, necessitating an effective sample of 40 patients (20 in each arm), with 5% significance and 90% power [56]. Based on the reported intra-cluster coefficient (0.02), [43], and a design effect of 1.18, to achieve an effective sample size of 40, an actual sample size of 48 was needed (Killip et al 2004) [58]. Ten patients in each of 6 clusters (GP practices) allows for 20% loss to follow up (S3 file). |
| 4) practice needs to be included as a random intercept in the regression models to account for clustering. | Thank you for this suggestion. The GP practice variable was included as a random intercept in all 5 GLMM analyses to allow for the multi-level structure of the data. Methods text now states ((line 187 *et seq*):  … assessed using multilevel generalized linear mixed models (GLMM), accounting for variability across GP practices by including practice as a random intercept [60]. We adjusted for age, deprivation and comorbidity. Data on age and deprivation were dichotomised: patients aged 65-75 (reference group) were compared with older patients; those in the most deprived WIMD quintile (reference group) were compared all with other patients, reflecting the study population. Comorbidity at baseline was summarised by ‘Number of long-term conditions’, entered as a continuous variable.  Table 5 has been reworked, replacing the previous adjusted analyses. Full models, as generated in spss, have been included in S4 file to ensure transparency.  Results were essentially unchanged when adjusted analyses from the GLMMs were substituted for the binary logistic regression models. As expected, confidence intervals widened.  Our professor of statistics has checked this addition to the paper, and is now included as a co-author. |
| 5) it’s not clear how missing data were dealt with. The consort statement has a section on that. were all data complete? If yes please state so. If not why weren’t multiple imputation approaches considered. | We agree this is a very important consideration, and apologise for any lack of clarity. We have further detailed where missing data arose. We explain (line 218 *et seq*):  Data collection from medical notes was complete. Missing data arose in the ADRe questionnaire when: a) participants completed ADRe by telephone or were unable to stand it was impossible to obtain measurements of all vital signs (5/27 respondents); b) one questions were designated as ‘discretionary’ due to cultural sensitivities: the question on sexual dysfunction was omitted by 15/27 respondents; all other questions were complete. These data were not subjected to inferential analyses. (Further details are in S3 file.) Differences between those responding and not responding militated against transferability of responses, and hence data imputation. |
| 6) why was age and WIMD dichotomised? There is no rationale for this and it reduced power further. | Thank you for highlighting this issue. We have now expanded the ‘limitations’ section of the manuscript to address concerns. As we acknowledge, the small sample size in our trial limited the deployment of covariates. We avoided low numbers in the cells of our models, as far as possible.  All participants were aged ≥65, indicating that a continuous variable would be inappropriate. In both arms, age was not normally distributed. We have added this information as a note to Table 1.  Shapiro- Wilk test for data distribution: intervention arm P=0.01; control arm P=0.01. We have also supported the use of 75 as a ‘cut point’ with a reference .  We have added a further note to Table 1:  Dichotomisation of age was based on definitions of premature death, used in UK statistics [62].  WIMD is a categorical variable. Most participants were in the most deprived quintile (Table 1). Numbers in the other 4 quintiles ranged between 4 and 0. Local knowledge of the research setting confirms the validity of this dichotomisation. We have added a note to Table 1:  There were 0-4 participants in each of the other four quintiles.  We agree that these measure to promote parsimony and minimise over-fitting reduce the power of the analyses. We now acknowledge this under ‘limitations’ (line 553 *et seq*).  Corollaries of the small sample size were low counts in some categories, necessitating simplification of variables, and restriction in the number of covariates. |
| And also any other covariate with imblalanced needs to be included. | Thank you for highlighting this. We have augmented the ‘limitation’ section of the discussion to encompass this important consideration.  We are limited by the size of the data set, and the numbers of participants with each outcome. Our lived experience of valleys’ communities suggests that we have included the most important modifiable risk factors that might help practitioners to identify needs.  We have expanded (line 553 *et seq*)  Corollaries of the small sample size were low counts in some categories, necessitating simplification of variables, and restriction in the number of covariates. Covariates selected reflect the important considerations in our communities. The diverse domains of the WIMD obviate the necessity of considering each separately in small studies. The need to narrow confidence intervals and reduce the risk of type 1 errors by introducing further covariates was balanced against the risk of over-fitting, which would limit the validity of findings [99]. We acknowledge that our model, based on 57 participants, is vulnerable to over-interpretation and inclusion of secondary outcomes, without adjusting for multiple comparisons, increases the likelihood of reporting misleading positive results. However, statistical findings should be considered alongside the narrative accounts of patient benefit when determining the practical adequacy of ADRe [100] |

Minor

| 11) WIMD needs to be defined for international readers. Area level measure   what domains it includes  what is the level on which calculated etc (Also what year used). | Thank you, we have augmented the text accordingly (line 174 *et seq*):  The Wales 2019 Index of Multiple Deprivation (WIMD) is a composite of eight domains (or types) of deprivation: a) Income b) Employment c) Health d) Education e) Access to Services f) Community Safety g) Physical Environment h) Housing. Each domain is compiled from several different indicators, and the domains are combined to give a single rank for the local area [59]. |
| --- | --- |
| 2) avoid the use of McNemar’s test and any other test that focuses on statistical significance. The regression model is adequate for that  with the baseline scores/values included as covariates. This allows obtaining effect sizes and confidence intervals which are more informative than p-values  especially in analyses that are underpowrered (e.g. secondary outcocomes). This to some extent addresses the type-I error inflation – this needs to be discussed as a limitation and also all analyses except primary outcome need to be discussed as exploratory. | Thank you. The testing of symptoms between before and after the intervention cannot be accommodated in the regression models because we do not have information on the signs and symptoms listed on ADRe for the control arm. The number of long-term conditions listed in the medical records is a covariate.  We acknowledge the limitations of McNemar’s test, and, on the advice of the reviewer, have removed it from the paper.  All trial outcomes were pre-specified in the protocol [44].  We agree that secondary outcomes inflate the risk of type I errors, and, as suggested, added this point to the ‘limitations’ (line 558 *et seq*):  We acknowledge that our model, based on 57 participants, is vulnerable to over-interpretation and inclusion of secondary outcomes, without adjusting for multiple comparisons, increases the likelihood of reporting misleading positive results. However, statistical findings should be considered alongside the narrative accounts of patient benefit when determining the practical adequacy of ADRe [100]. |

Reviewer #2: This is an interesting article, indeed a “better system”

| However, I am unable to access and review the ADRe profile itself, which I believe is the essence of this paper. The link should be easily accessible, or a version posted as supplementary material to enable a proper understanding of the applicability of such a tool. | Thank you. Positive support is much appreciated.  We apologise. ADRe is normally available by email from our website. A direct link was included in reference 46. We have now clarified and augmented reference [46]. The problems listed on ADRe are reproduced in S3 file. This has prompted us to expand the information in S4 file, S2 table. |
| --- | --- |
| Line 86- actual is underlined?? | Thank you. This was intended for emphasis. Following reviewer’s advice, the underline has been removed. |
| Different fonts and sizes in various parts of the article, these should be corrected. | Thank you. We have attended to this. |
| Table 3- 1,11 or 1.11 . please clarify | Thank you for the detailed scrutiny. Typographical error. We apologise. |

Reviewer #3:

| 1.Cluster randomization concerns  The study is described as a cluster-randomized trial involving six GP practice clusters; however, only one cluster consisting of six practices was ultimately recruited. This substantially undermines the rationale for cluster randomization and raises concerns about selection bias and external validity. Please clarify whether clustering effects were accounted for and discuss the implications for statistical inference | Thank you. We apologise for confusion. The two uses of the term ‘clustering’ have been explicated. We have two distinct types of “clusters”. To avoid ambiguity. GP clusters have now been re-named GP groups, retaining the term ‘cluster’ solely as a statistical term. As stated in our protocol [44 p.3, 3.1], our clusters were GP practices. Data have been re-analysed to account for clustering. Under ‘analysis’ (line 187) we now state: ... was assessed using multilevel generalized linear mixed models (GLMM), accounting for variability across GP practices by including practice as a random intercept [60]. \| |
| --- | --- |
| 2. Deviation from the planned study design |  |
| The protocol planned recruitment of six clusters, but the final study included only one cluster. This represents a major deviation from the protocol and may invalidate the sample size calculation and power assumptions. The impact of this deviation should be explicitly discussed. | We apologise for this lack of clarity. In statistical terms, we recruited 6 clusters as planned. However, these were all from a single GP group. We hope that the above adjustment of terminology explicates the situation. |
| 3. Unit of analysis vs. design mismatch |  |
| Although the trial was designed as a cluster RCT, the analysis was conducted at the individual patient level due to insufficient clusters. This raises concerns about inflated type I error and potential violation of the original trial design assumptions. | Thank you. On advice of the statistician, we have re-analysed the data to account for clustering. Methods text now states (line 187 *et seq*):  … was assessed using multilevel generalized linear mixed models (GLMM), accounting for variability across GP practices by including practice as a random intercept [60].  Table 5 has been reworked, replacing the previous adjusted analyses. Full models, as generated in spss, have been included in S4 file to ensure transparency.  Results were essentially unchanged when adjusted analyses from the GLMMs were substituted for the binary logistic regression models. As expected, confidence intervals widened.  We acknowledge the risk of type 1 error under ‘limitations’ (line 558 *et seq*).  We acknowledge that our model, based on 57 participants, is vulnerable to over-interpretation and inclusion of secondary outcomes, without adjusting for multiple comparisons, increases the likelihood of reporting misleading positive results. However, statistical findings should be considered alongside the narrative accounts of patient benefit when determining the practical adequacy of ADRe [100]. |
| 4. Lack of ADR causality assessment |  |
| The manuscript frequently attributes symptoms to medications, yet no formal causality assessment method (e.g., WHO-UMC, Naranjo) was used. The findings therefore represent symptom detection rather than confirmed ADR identification, and this distinction should be clearly stated. | Thank you. We agree that ADRe focuses on symptoms that concern patients, regardless of aetiology.  We have clarified in the ‘background’ section (line 78 *et seq*):  Our instrument, the ADRe Profile, integrates the expertise of nurses, pharmacists, prescribers, and patients in the surveillance and prevention of potential ADRs, ADEs, and health problems [8, 42-44].  We acknowledge that there were no causality assessments under ‘limitations’ (line 448 *et seq*).  The provenance of problems identified was not explored with de-challenge and re-challenge, the ‘gold standard’ for ADR detection. This study was not designed to meet the criteria for ADR signal detection [107].  And (line 577 *et seq*):  Symptom aetiology was often uncertain or mixed (for example, patient 403 was prescribed 40mg furosemide, and hearing improved when ear wax was removed); however, there was a statistically significant reduction in problems following ADRe administration.  In this context, we, and the local ethics committee, felt that any rechallenge to confirm symptom aetiology would be unethical. |
| 5. Definition and clinical relevance of “problems” |  |
| The study reports 388 problems among 27 patients, averaging over 14 problems per patient, most of which were not recorded in medical records. It is unclear how a “problem” was defined and whether minor symptoms were counted alongside clinically significant ADRs. Greater clarification of problem classification and clinical relevance is required. | Thank you. We have clarified and listed the problems ADRe aims to address (line 143 *et seq*):  ‘Health problems’ were defined as the signs and symptoms listed on ADRe [46] [44 p.5] and listed in S3 file.  We note under ‘limitations’ (line 570 *et seq*):  Self-reported outcomes are vulnerable to social desirability bias, and subjective interpretation of symptoms [101], but there is no reason to assume this would change between data collection rounds.  Severity of signs and symptoms is assessed by patients and clinicians. We now acknowledge this (line 582 *et seq*):  Severity of signs and symptoms was assessed by patients and clinicians, not ADRe; inclusion of trivial symptoms was minimized by using problems in medical records as the primary outcome. |
| 6. Potential overinterpretation of clinical benefit |  |
| The manuscript states that all patients in the intervention arm benefited or expected to benefit, which appears overly strong given that many interventions consisted of referrals or monitoring rather than confirmed clinical improvement. The interpretation of benefit should be more cautious and evidence-based. | Thank you. All 27 patients benefitted or expected to benefit, some more so than others. We state that some gains were modest e.g. relief of itching.  Our interpretation of the implications is based on the accumulation of evidence on the clinical impact of ADRe, not just this study. Following this comment, we have modified our conclusion (line 607 *et seq*):  Taken with previous RCTs [43, 47] and observational studies [8, 49-52, 83], we suggest that ADRe consistently identifies and addresses important problems unrecorded elsewhere, and improves outcomes for many patients by facilitating timely intervention. |
| 7. Generalizability limitations |  |
| The study was conducted in a single economically deprived region with a very small number of participating practices. The generalizability of the findings to broader primary care populations requires further discussion. | Thank you. This important point warrants its own paper [102]. The confines of the word limit preclude a lengthy discussion of this acknowledged problem. We have added (line 5653 *et seq*):  Transferability of evidence based practice and trial findings from affluent to deprived communities or from urban to rural settings and *vice versa* should not be assumed without further research in the target setting [102]. |
| 8. Feasibility and implementation challenges |  |
| The study notes that many clinicians declined participation due to concerns about workload. This suggests potential implementation barriers, which should be more fully discussed in the context of real-world adoption. | This is an important topic, which warrants its own paper [116]. We agree that ADRe faces implementation barriers, as discussed in a paper earlier this year, and have expanded (line 597 *et seq*):  The reluctance of primary care practices to engage with ADRe [52], and review of the contextual challenges [116], indicate that further work on implementation is needed. |
| 9. Inconsistent age criteria |  |
| The abstract states >64 years, while the methods state ≥65 years. Please standardize the eligibility criteria. | Thank you, this has been done. |
| 10. Terminology clarity |  |
| The manuscript uses ADR, ADE, symptoms, and problems interchangeably. These represent distinct concepts in pharmacovigilance and should be used consistently. | Thank you. Terminology is clarified in the glossary (was List 1) ‘Definitions’.  When referring to the literature, we have used the terms adopted by the original authors. We agree that this introduces some inevitable inconsistency, but prefer to adhere to authors’ terminology.  We have clarified that ADRe works on ADRs, ADEs and problems of other aetiology. |
| 11. Polypharmacy definition |  |
| Please clarify whether PRN medications were included in the count of long-term medicines. | Thank you for this attention to detail. We have added (line 125 *et seq*):  Where medicines were prescribed as PRN (*pro re nata)*, patients were asked regarding administration; only medicines taken daily were included. |
| 12. Intervention standardization |  |
| Additional detail is needed on training of pharmacists and standardization of ADRe review procedures. | Only one pharmacist was involved. It was agreed that no formal training was needed. |
| 13. Outcome hierarchy |  |
| It would be helpful to distinguish clearly between process outcomes (e.g., referrals, investigations) and clinical outcomes (symptom resolution, deprescribing). | We agree that both process and clinical outcomes were assessed.  Investigations are documented in Table 4.  Changes in signs and symptoms are listed in Table 6 and S4 file, S2 table. To acknowledge their inter-relationship, we have added (line 478 *et seq*):  Through both process (Table 4) and clinical changes (Table 6, S4 File, S1, S2 Tables), ADRe complemented pharmacist reviews – it did not replace them.  Referrals were considered an effective strategy to address problems. The differences in clinical problems between the first and second administration of ADRe (Table 6, S4 File, S1, S2 Tables) also indicated clinical gain.  ADRe affected some (not all) process outcomes (laboratory testing (Table 4) and deprescribing (Tables 3, 5)), and clinical outcomes, as listed on ADRe (S3 file) as measured by the numbers of problems addressed (Tables 3,5). Where referrals were made, clinical gain usually followed from changes in advice or prescribing.  There were also differences in clinical problems between the trial’s start and finish (Table 6, S4 File 4, S1,S2 Tables). |
| 14. Phases of study |  |
| It is mentioned in the protocol that the study would be conducted in 3 phases. Wa it maintained while conducting the study?Please provide the timeline for the same. | The protocol paper [44] indicates that 2 phases were planned. This paper reports Phase 1 plus the completed aspects of phase 2. Phase 2 was not completed in its entirety. However, earlier stakeholder evaluations are available [8, 50]. The trial was preceded by reliability and validity work [73] and a feasibility study [52]. |
