# Supplementary material for: “A better system” – managing the adverse effects of prescribed medicines: Cluster-randomised controlled trial and process evaluation of the ADRe Profile in primary care
Source: PLoS One. 2026 Sep 18;21(9):e0356480. doi: 10.1371/journal.pone.0356480 (PMC13588343; doi:10.1371/journal.pone.0356480)
Supplement: Supplementary file 7 [file pone.0356480.s008.docx]

Response to reviews

Eyob Alemayehu Gebreyohannes, PhD
Academic Editor
PLOS One

Dear Dr. Gebreyohannes,

Thank you very much for considering our work. Our responses are below.

Yours,

Sue

**Journal Requirements:**

1. If the reviewer comments include a recommendation to cite specific previously published works, please review and evaluate these publications to determine whether they are relevant and should be cited. There is no requirement to cite these works unless the editor has indicated otherwise. NA

2. Please review your reference list to ensure that it is complete and correct. If you have cited papers that have been retracted, please include the rationale for doing so in the manuscript text, or remove these references and replace them with relevant current references. Any changes to the reference list should be mentioned in the rebuttal letter that accompanies your revised manuscript. If you need to cite a retracted article, indicate the article’s retracted status in the References list and also include a citation and full reference for the retraction notice. NA

**Additional Editor Comments:**

Revisit the reference to the 24 GP groups, as it may be unnecessary and potentially confusing for readers. Instead, rephrase the terminology and, where possible, provide a clearer description of the specific cluster included in the study. This would improve clarity and maintain focus on the study findings without introducing distracting detail.

***Thank you for this advice. I hope our amendments provide greater clarity.*** Our description has been curtailed by our commitment to ensure that the practices remain anonymous.

***( L***ine 117 *et seq*)

We recruited 60 patients from six primary care practices in one valley in South-West Wales where the pharmacist and general practitioners (GPs) were willing to review completed ADRe documents. The six practices worked as a group of collaborating doctors and allied professionals. The study area is amongst the most deprived in Wales for income, employment and health; however, it enjoys relatively good transport links. Over half the patients are amongst the 20% most deprived in terms of health, measured as long-term illness, premature death, cancer incidence, recorded long-term and mental health conditions, preterm birth and childhood obesity (RCGP 2022, Welsh government 2025). The practices in the upper valley were the most deprived.

[Note: HTML markup is below. Please do not edit.]

Reviewers' comments:

Reviewer's Responses to Questions

**Comments to the Author**
Questions 1-5 are all affirmative, and do not require a response from us.

6. Review Comments to the Author

Please use the space provided to explain your answers to the questions above. You may also include additional comments for the author, including concerns about dual publication, research ethics, or publication ethics. (Please upload your review as an attachment if it exceeds 20,000 characters)

| 1. **Reviewer #1:** I am satisfied with the authors' responses and the resulting changes to the paper. I have nothing else to add. | **Thank you for your support.** |
| --- | --- |
| 2. **Reviewer #2:** I think the reference to the 24 GP groups is not necessary and confusing, the authors should rephrase the terminology and describe if possible, the particular cluster that was used in the study. This does not distract from the findings. | Thank you for this insight. We have rewritten, line 117 *et seq*:  We recruited 60 patients from six primary care practices in one valley in South-West Wales where the pharmacist and general practitioners (GPs) were willing to review completed ADRe documents. The six practices worked as a group of collaborating doctors and allied professionals. The study area is amongst the most deprived in Wales for income, employment and health; however, it enjoys relatively good transport links. Over half the patients are amongst the 20% most deprived in terms of health, measured as long-term illness, premature death, cancer incidence, recorded long-term and mental health conditions, preterm birth and childhood obesity (RCGP 2022, Welsh government 2025). The practices in the upper valley were the most deprived.  Our description has been curtailed by our commitment to ensure that the practices remain anonymous. |
| 3. **Reviewer #3:** Thank you to the authors for the careful and constructive revision of the manuscript. The revised version is notably improved in terms of methodological clarity, statistical reporting, and discussion of limitations. I especially appreciate the re-analysis using GLMM to account for clustering effects, as well as the clearer distinction made between patient-reported symptoms/problems and confirmed adverse drug reactions. | **Thank you for your support.** |
| 4. The manuscript addresses an important and clinically relevant area in primary care pharmacovigilance and polypharmacy management among older adults. The patient-centred approach of the intervention, together with its emphasis on communication between patients, pharmacists, and prescribers, represents a meaningful strength of the study. | **Thank you for your support.** |
| 5. The additional clarifications regarding study design, terminology, handling of PRN medications, and differentiation between process and clinical outcomes have improved both the readability and interpretability of the manuscript. I also value the authors’ more balanced discussion of implementation challenges and feasibility in routine clinical practice. | **Thank you for your support.** |
| 6. A few concerns nevertheless remain, mainly related to the interpretation and framing of the findings. While the revised statistical approach is appropriate, the small number of participating practices and recruitment from a single GP group continue to limit the robustness and wider generalizability of the conclusions. In this regard, the study may be better framed as exploratory or pilot in nature. | We agree that this trial is exploratory in primary care. We have substituted ‘explored’ for ‘investigated’ (line 109) and described our models as ‘exploratory’ (line 563).  There is no funding for further work, so we are unable to term this ‘pilot work’. We acknowledge that ‘further work on implementation of these exploratory findings is needed.’ (line 604)  The evidence for adoption of ADRe becomes more convincing when considered alongside the work in other areas (lines 91-2, and 612), and patients’ views (line 504) |
| 7. In addition, some statements relating to “clinical benefit” and “improved outcomes” still appear somewhat stronger than the available evidence fully supports. Several of the reported benefits were linked to identification of symptoms, referrals, investigations, medication review, or monitoring processes, rather than confirmed reductions in medication-related harm. I would therefore encourage the authors to further moderate causal language throughout the manuscript and to consistently distinguish between: | We have endeavoured to comply, as detailed below.  We agree that referrals do not always confer clinical benefit; however, expert opinion is welcomed by patients, and care usually improves. We now acknowledge that outcomes of referrals (mainly to medical specialists) were not available within the study timeframe. Line 590 *et seq* now reads:  The study’s timeframe limited exploration of all associations between symptoms and medicines identified in ADRe decision support and the outcomes of referrals to secondary care.  We do not mention this is the paper, but our participants were unable to expedite their referrals to consultants by paying for private healthcare, leading to long delays.  We have qualified our claims by inserting ‘some’, for example, line 599:  This addressed some instances of unmonitored avoidable medicine-related harm … |
| 8. -patient-reported symptoms/problems, | We agree that ADRe only monitors patient-reported problems. Where patients live in their own homes, it is impossible to confirm reports of falls or insomnia. To clarify, we have changed the section heading from ‘clinical gain’ to ‘clinical gains reported by patients’. (line 350)  We now acknowledge that patient-reported symptoms fluctuate for reasons unrelated to pharmacotherapy. Line 454:  Other gains were more modest e.g. relief of itching following prescription for an emollient (ID 9), and some symptoms abated for no reason apparent to researchers e.g. insomnia and vivid dreams subsided (ID 2).  For the primary outcome, we used the medical notes. While we can be confident regarding actions taken and prescription changes, we can only infer associations with patient-reported problems. |
| 9. -potential medication-related concerns, | In practice, it is difficult to assign aetiology, as noted (line 577):  Symptom aetiology was often uncertain or mixed.    By listing putative explanations for resolution of problems, Table 6 shows that many improvements were not due to changes in prescriptions. |
| 10. -and confirmed ADRs/ADEs. | We agree and acknowledge (line 58) that we were unable to meet the criteria of de-challenge and re-challenge. Neither we nor the research ethics committee consider re-challenge to be appropriate in primary care trials of complex interventions, due to the risks involved. We acknowledge under ‘limitations’, line 577:  The provenance of problems identified was not explored with de-challenge and re-challenge, the ‘gold standard’ for ADR detection.  For some problems the balance of opinion indicates iatrogenic aetiology. Examples include: resolution of paraesthesia following B12 supplementation for a patient using metformin long-term, resolution of hallucinations on discontinuation of flecainide, resolution of abnormal movements following mycophenolate or gabapentin dose reduction. |
| 11. In Table 3, terms such as “after intervention” may be slightly confusing when referring to the control arm, since the control group did not receive the intervention. Consider using terminology such as “follow-up” or “post-study assessment” for clarity. | Thank you for this detailed consideration. We agree. We have adopted the terms ‘baseline’ and ‘follow-up’.  We have added a note to table 3:  ** Baseline data were extracted before the intervention was delivered. Follow-up data were extracted after the intervention was delivered in the intervention arm. Control arm participants received usual care throughout; they did not receive the intervention.*  We have adjusted line 99 *et seq* to reflect the change in terminology:  We explored: differences between trial arms in problems and prescriptions in the practice medical records at baseline and follow-up (before and after the intervention was delivered in one arm);    We have also clarified in line 157:  In both arms, at baseline (before) and follow-up (after ADRe use in one arm), using medical records … |
| 12. In Table 5, the distinction between “Deprescribing, medicines discontinued” and “Deprescribing, medicines stopped or doses reduced” is not immediately clear and may be confusing to readers, as the latter appears to include the former. Kindly clarify whether these represent separate or cumulative outcomes and consider revising the wording for greater clarity. | Thank you. We have added a note to table 5.  † This outcome measured both discontinued medicines (as above) plus dose reductions. |
| 13. In the response to reviewers, it is mentioned that only one pharmacist was involved in the study/intervention. However, in the Results section (process evaluation), references are made to “Pharmacist 1” and “Pharmacist 2.” Kindly clarify the number and roles of pharmacists involved in the intervention and process evaluation, as this currently appears inconsistent and may confuse readers. | Thank you. We have clarified that both pharmacists were working with the research team.  Line 209:  … two pharmacists working with the ADRe project [Logan et al 2025, 2026] …  We are obliged to restrict details to preserve anonymity.  Transcripts from all interviews in all phases of the project are available via a non-author email: [library-digital@swansea.ac.uk](mailto:library-digital@swansea.ac.uk) or the corresponding authors.e.jordan@swansea.ac.uk.  URL is: [https://zenodo.org/records/19736675](https://eur03.safelinks.protection.outlook.com/?url=https%3A%2F%2Fzenodo.org%2Frecords%2F19736675&data=05%7C02%7CS.E.Jordan%40Swansea.ac.uk%7C83aeda658df546be630708dea213df7c%7Cbbcab52e9fbe43d6a2f39f66c43df268%7C0%7C0%7C639126403384132438%7CUnknown%7CTWFpbGZsb3d8eyJFbXB0eU1hcGkiOnRydWUsIlYiOiIwLjAuMDAwMCIsIlAiOiJXaW4zMiIsIkFOIjoiTWFpbCIsIldUIjoyfQ%3D%3D%7C0%7C%7C%7C&sdata=SQHlCHq4mjoPeWvQltF78DLlqRiwaYFsjOgm0F%2BzhCk%3D&reserved=0)  Doi is: 10.5281/zenodo.19736674 ([https://doi.org/10.5281/zenodo.19736674](https://eur03.safelinks.protection.outlook.com/?url=https%3A%2F%2Fdoi.org%2F10.5281%2Fzenodo.19736674&data=05%7C02%7CS.E.Jordan%40Swansea.ac.uk%7C83aeda658df546be630708dea213df7c%7Cbbcab52e9fbe43d6a2f39f66c43df268%7C0%7C0%7C639126403384153949%7CUnknown%7CTWFpbGZsb3d8eyJFbXB0eU1hcGkiOnRydWUsIlYiOiIwLjAuMDAwMCIsIlAiOiJXaW4zMiIsIkFOIjoiTWFpbCIsIldUIjoyfQ%3D%3D%7C0%7C%7C%7C&sdata=f%2Bug4JV6U367VhtwegaM7lf%2Fo9v7UqOfnk5HwnJu1f8%3D&reserved=0)) |
| Overall, this is a thoughtful and clinically valuable study that contributes meaningfully to ongoing discussions on safer prescribing and person-centred monitoring in primary care. With some further refinement of the conclusions and interpretation, the manuscript will be further strengthened. | Thank you for your support. We have endeavoured to adopt the helpful suggestions of the expert reviewers. For example, line 613 now reads:  ADRe … reduces health-related problems for many patients … |
