# Supplementary material for: “A better system” – managing the adverse effects of prescribed medicines: Cluster-randomised controlled trial and process evaluation of the ADRe Profile in primary care
Source: PLoS One. 2026 Sep 18;21(9):e0356480. doi: 10.1371/journal.pone.0356480 (PMC13588343; doi:10.1371/journal.pone.0356480)
Supplement: Supplementary file 8 [file pone.0356480.s009.docx]

Response to reviewers

Eyob Alemayehu Gebreyohannes, PhD
Academic Editor
PLOS One

28.7.26

Dear Dr. Gebreyohannes,

Thank you very much for your careful consideration of our work. We have amended the abstract as suggested. Our responses are below.

Yours,

Sue

PONE-D-26-02770R2

“A better system” – managing the adverse effects of prescribed medicines: cluster-randomised controlled trial and process evaluation of the ADRe Profile in primary care

PLOS One

Dear Dr. Jordan,

Thank you for submitting your manuscript to PLOS ONE. After careful consideration, we feel that it has merit but does not fully meet PLOS ONE’s publication criteria as it currently stands. Therefore, we invite you to submit a revised version of the manuscript that addresses the points raised during the review process.

==============================
**ACADEMIC EDITOR:**

There is only one minor edits you need to make before the paper can be formally accepted for publication. In the abstract section, change "We recruited 1 of 24 general practice groups (each comprising 6 practices) and 60 of 170 patients 40 approached." To enhance clarity, re-write it as "We recruited 1 general practice group comprising 6 practices and 60 patients."

***Thank you, this has been done.***

Please submit your revised manuscript by Sep 10 2026 11:59PM If you will need more time than this to complete your revisions, please reply to this message or contact the journal office at [plosone@plos.org](mailto:plosone@plos.org). When you're ready to submit your revision, log on to <https://www.editorialmanager.com/pone/> and select the 'Submissions Needing Revision' folder to locate your manuscript file.

Please include the following items when submitting your revised manuscript:

- A letter that responds to each point raised by the academic editor and reviewer(s). You should upload this letter as a separate file labeled 'Response to Reviewers'.
- A marked-up copy of your manuscript that highlights changes made to the original version. You should upload this as a separate file labeled 'Revised Manuscript with Track Changes'.
- An unmarked version of your revised paper without tracked changes. You should upload this as a separate file labeled 'Manuscript'.

***We have complied with all three.***

If you would like to make changes to your financial disclosure, please include your updated statement in your cover letter. Guidelines for resubmitting your figure files are available below the reviewer comments at the end of this letter. NA

If applicable, we recommend that you deposit your laboratory protocols in protocols.io to enhance the reproducibility of your results. Protocols.io assigns your protocol its own identifier (DOI) so that it can be cited independently in the future. For instructions see: [https://journals.plos.org/plosone/s/submission-guidelines#loc-laboratory-protocols](https://eur03.safelinks.protection.outlook.com/?url=https%3A%2F%2Ftrack.editorialmanager.com%2FCL0%2Fhttps%3A%252F%252Fjournals.plos.org%252Fplosone%252Fs%252Fsubmission-guidelines%2523loc-laboratory-protocols%2F1%2F010f019fa688eca4-02d33b7d-a7bd-44bb-abd7-9e645eb34d76-000000%2FTRB_gpGMn6BW7Pbo7jFYACZEseKE_0q3SVON3qsB58g%3D258&data=05%7C02%7Cs.e.jordan%40swansea.ac.uk%7Caa6efd663810425d8bab08deec4f3d64%7Cbbcab52e9fbe43d6a2f39f66c43df268%7C0%7C0%7C639208022254375122%7CUnknown%7CTWFpbGZsb3d8eyJFbXB0eU1hcGkiOnRydWUsIlYiOiIwLjAuMDAwMCIsIlAiOiJXaW4zMiIsIkFOIjoiTWFpbCIsIldUIjoyfQ%3D%3D%7C0%7C%7C%7C&sdata=0BnxPB4vWWThKJpVo%2FA8VmCZdIT0Dp6tivR2YLmgfhQ%3D&reserved=0). Additionally, PLOS ONE offers an option for publishing peer-reviewed Lab Protocol articles, which describe protocols hosted on protocols.io. Read more information on sharing protocols at [https://plos.org/protocols?utm_medium=editorial-email&utm_source=authorletters&utm_campaign=protocols](https://eur03.safelinks.protection.outlook.com/?url=https%3A%2F%2Ftrack.editorialmanager.com%2FCL0%2Fhttps%3A%252F%252Fplos.org%252Fprotocols%253Futm_medium%3Deditorial-email%2526utm_source%3Dauthorletters%2526utm_campaign%3Dprotocols%2F1%2F010f019fa688eca4-02d33b7d-a7bd-44bb-abd7-9e645eb34d76-000000%2F7nuO6ZFZVZ1C73IfmTsajwcpc4ovbi94iWb1ZqiYINU%3D258&data=05%7C02%7Cs.e.jordan%40swansea.ac.uk%7Caa6efd663810425d8bab08deec4f3d64%7Cbbcab52e9fbe43d6a2f39f66c43df268%7C0%7C0%7C639208022254403703%7CUnknown%7CTWFpbGZsb3d8eyJFbXB0eU1hcGkiOnRydWUsIlYiOiIwLjAuMDAwMCIsIlAiOiJXaW4zMiIsIkFOIjoiTWFpbCIsIldUIjoyfQ%3D%3D%7C0%7C%7C%7C&sdata=HTsWUflEZCwN%2BIy9Z%2BDVw3KsBXUJ9kwvyZf%2FpdGVSiY%3D&reserved=0). NA

As the corresponding author, your ORCID iD is verified in the submission system and will appear in the published article. PLOS supports the use of ORCID, and we encourage all coauthors to register for an ORCID iD and use it as well. Please encourage your coauthors to verify their ORCID iD within the submission system before final acceptance, as unverified ORCID iDs will not appear in the published article. *Only* the individual author can complete the verification step; PLOS staff *cannot* verify ORCID iDs on behalf of authors. ***This has been done.***

We look forward to receiving your revised manuscript.

Kind regards,

Eyob Alemayehu Gebreyohannes, PhD
Academic Editor
PLOS One

Journal Requirements:

If the reviewer comments include a recommendation to cite specific previously published works, please review and evaluate these publications to determine whether they are relevant and should be cited. There is no requirement to cite these works unless the editor has indicated otherwise.  NA

Please review your reference list to ensure that it is complete and correct. If you have cited papers that have been retracted, please include the rationale for doing so in the manuscript text, or remove these references and replace them with relevant current references. Any changes to the reference list should be mentioned in the rebuttal letter that accompanies your revised manuscript. If you need to cite a retracted article, indicate the article’s retracted status in the References list and also include a citation and full reference for the retraction notice.

***The list has been checked.***

Additional Editor Comments:

There is only one minor edits you need to make before the paper can be formally accepted for publication. In the abstract section, change "We recruited 1 of 24 general practice groups (each comprising 6 practices) and 60 of 170 patients 40 approached." To enhance clarity, re-write it as "We recruited 1 general practice group comprising 6 practices and 60 patients."

***Thank you, we have made the necessary change.***

[Note: HTML markup is below. Please do not edit.]

Reviewers' comments: (I have deleted all the positive responses to reduce the text in this document.)

6. Review Comments to the Author

Please use the space provided to explain your answers to the questions above. You may also include additional comments for the author, including concerns about dual publication, research ethics, or publication ethics. (Please upload your review as an attachment if it exceeds 20,000 characters)

Reviewer #1: nothing else to add.........................................................................................................

Reviewer #2: The 1The abstract section needs to revised as well, particularly the methodology which still includes 1 in 24 practices. This needs clarification.

***Thank you, we have amended, following the editor’s suggestions.***

7. PLOS authors have the option to publish the peer review history of their article ([what does this mean?](https://eur03.safelinks.protection.outlook.com/?url=https%3A%2F%2Ftrack.editorialmanager.com%2FCL0%2Fhttps%3A%252F%252Fjournals.plos.org%252Fplosone%252Fs%252Feditorial-and-peer-review-process%2523loc-peer-review-history%2F1%2F010f019fa688eca4-02d33b7d-a7bd-44bb-abd7-9e645eb34d76-000000%2FN2WhD755yPmdS25-9G-MrcdgW2-aiMIl2QqFaNswPGc%3D258&data=05%7C02%7Cs.e.jordan%40swansea.ac.uk%7Caa6efd663810425d8bab08deec4f3d64%7Cbbcab52e9fbe43d6a2f39f66c43df268%7C0%7C0%7C639208022254440512%7CUnknown%7CTWFpbGZsb3d8eyJFbXB0eU1hcGkiOnRydWUsIlYiOiIwLjAuMDAwMCIsIlAiOiJXaW4zMiIsIkFOIjoiTWFpbCIsIldUIjoyfQ%3D%3D%7C0%7C%7C%7C&sdata=Ue7ifhbDidS7531N1dwzpz2rBn4HYnol9HbYQf17wj8%3D&reserved=0)). If published, this will include your full peer review and any attached files.

If you choose “no”, your identity will remain anonymous but your review may still be made public.


**Do you want your identity to be public for this peer review?** For information about this choice, including consent withdrawal, please see our [Privacy Policy](https://eur03.safelinks.protection.outlook.com/?url=https%3A%2F%2Ftrack.editorialmanager.com%2FCL0%2Fhttps%3A%252F%252Fwww.plos.org%252Fprivacy-policy%2F1%2F010f019fa688eca4-02d33b7d-a7bd-44bb-abd7-9e645eb34d76-000000%2FlIQjWJqpRri_dFRqddE2D7QOFjjKcbaVv7jjH0RVgJ8%3D258&data=05%7C02%7Cs.e.jordan%40swansea.ac.uk%7Caa6efd663810425d8bab08deec4f3d64%7Cbbcab52e9fbe43d6a2f39f66c43df268%7C0%7C0%7C639208022254458711%7CUnknown%7CTWFpbGZsb3d8eyJFbXB0eU1hcGkiOnRydWUsIlYiOiIwLjAuMDAwMCIsIlAiOiJXaW4zMiIsIkFOIjoiTWFpbCIsIldUIjoyfQ%3D%3D%7C0%7C%7C%7C&sdata=gh9hO18H3QP%2BwLa%2FTdm2hxMXeL8Mn7K7taDBeWqNIzk%3D&reserved=0).

Reviewer #1: No

Reviewer #2: No

To ensure your figures meet our technical requirements, please review our figure guidelines: <https://journals.plos.org/plosone/s/figures>

You may also use PLOS’s free figure tool, NAAS, to help you prepare publication quality figures: <https://journals.plos.org/plosone/s/figures#loc-tools-for-figure-preparation>.

NAAS will assess whether your figures meet our technical requirements by comparing each figure against our figure specifications.

***Thank you. Figures were re-checked, and one needed to be reformatted. The compliant version has been uploaded. There is no change in the text.***
