# Supplementary material for: “A better system” – managing the adverse effects of prescribed medicines: Cluster-randomised controlled trial and process evaluation of the ADRe Profile in primary care
Source: PLoS One. 2026 Sep 18;21(9):e0356480. doi: 10.1371/journal.pone.0356480 (PMC13588343; doi:10.1371/journal.pone.0356480)
Supplement: Peer review history

# Peer review: Decision Letter 0

**DOI:** 10.1371/journal.pone.0356480.r001

**Authors:** Eyob Alemayehu Gebreyohannes

15 Mar 2026

Dear Dr. Jordan,

Thank you for submitting your manuscript to PLOS ONE. After careful consideration, we feel that it has merit but does not fully meet PLOS ONE’s publication criteria as it currently stands. Therefore, we invite you to submit a revised version of the manuscript that addresses the points raised during the review process.

==============================

**ACADEMIC EDITOR:**

While the reviewers recognise the potential relevance of your study, they have raised several important concerns that need to be addressed before the manuscript can be considered further.

In particular, the reviewers highlighted the following key issues among others:

- Academic writing and clarity: The manuscript would benefit from substantial editing to improve the clarity, structure, and overall academic writing.
- Primary outcome: The primary outcome of the study should be explicitly stated and consistently presented.
- Power calculation and clustering
- Handling of missing data
- The manuscript should clearly explain any deviations from the originally planned study design and discuss their potential implications.

Please carefully revise your manuscript in light of the reviewers’ comments and provide a detailed, point-by-point response to each comment.

==============================

Please submit your revised manuscript by Apr 29 2026 11:59PM. If you will need more time than this to complete your revisions, please reply to this message or contact the journal office at plosone@plos.org. When you're ready to submit your revision, log on to https://www.editorialmanager.com/pone/ and select the 'Submissions Needing Revision' folder to locate your manuscript file.

Please include the following items when submitting your revised manuscript:

- A letter that responds to each point raised by the academic editor and reviewer(s). You should upload this letter as a separate file labeled 'Response to Reviewers'.
- A marked-up copy of your manuscript that highlights changes made to the original version. You should upload this as a separate file labeled 'Revised Manuscript with Track Changes'.
- An unmarked version of your revised paper without tracked changes. You should upload this as a separate file labeled 'Manuscript'.

If you would like to make changes to your financial disclosure, please include your updated statement in your cover letter. Guidelines for resubmitting your figure files are available below the reviewer comments at the end of this letter.

If applicable, we recommend that you deposit your laboratory protocols in protocols.io to enhance the reproducibility of your results. Protocols.io assigns your protocol its own identifier (DOI) so that it can be cited independently in the future. For instructions see: https://journals.plos.org/plosone/s/submission-guidelines#loc-laboratory-protocols. Additionally, PLOS ONE offers an option for publishing peer-reviewed Lab Protocol articles, which describe protocols hosted on protocols.io. Read more information on sharing protocols at https://plos.org/protocols?utm_medium=editorial-email&utm_source=authorletters&utm_campaign=protocols.

We look forward to receiving your revised manuscript.

Kind regards,

Eyob Alemayehu Gebreyohannes, PhD

Academic Editor

PLOS One

Journal Requirements:

When submitting your revision, we need you to address these additional requirements.

1. Please ensure that your manuscript meets PLOS ONE's style requirements, including those for file naming. The PLOS ONE style templates can be found at

https://journals.plos.org/plosone/s/file?id=wjVg/PLOSOne_formatting_sample_main_body.pdf and

https://journals.plos.org/plosone/s/file?id=ba62/PLOSOne_formatting_sample_title_authors_affiliations.pdf

2. Thank you for stating the following in the Acknowledgments Section of your manuscript:

“This work was supported by Swansea University (Centenary Research Excellence Scholarship), sponsor reference RIO 034-20. The funders had no role in study design, data collection and analysis, interpretation of data, decision to publish, or preparation of the manuscript.”

We note that you have provided funding information that is not currently declared in your Funding Statement. However, funding information should not appear in the Acknowledgments section or other areas of your manuscript. We will only publish funding information present in the Funding Statement section of the online submission form.

Please remove any funding-related text from the manuscript and let us know how you would like to update your Funding Statement. Currently, your Funding Statement reads as follows:

“This work was supported by Swansea University (Centenary Research Excellence Scholarship), sponsor reference RIO 034-20. The funders had no role in study design, data collection and analysis, interpretation of data, decision to publish, or preparation of the manuscript.”

Please include your amended statements within your cover letter; we will change the online submission form on your behalf.

3. Thank you for stating the following financial disclosure:

“This work was supported by Swansea University (Centenary Research Excellence Scholarship), sponsor reference RIO 034-20. The funders had no role in study design, data collection and analysis, interpretation of data, decision to publish, or preparation of the manuscript.”

We note that one or more of the authors is affiliated with the funding organization, indicating the funder may have had some role in the design, data collection, analysis or preparation of your manuscript for publication; in other words, the funder played an indirect role through the participation of the co-authors. If the funding organization did not play a role in the study design, data collection and analysis, decision to publish, or preparation of the manuscript and only provided financial support in the form of authors' salaries and/or research materials, please do the following:

1. Review your statements relating to the author contributions, and ensure you have specifically and accurately indicated the role(s) that these authors had in your study. These amendments should be made in the online form.

2. Confirm in your cover letter that you agree with the following statement, and we will change the online submission form on your behalf:

“The funder provided support in the form of salaries for authors [insert relevant initials], but did not have any additional role in the study design, data collection and analysis, decision to publish, or preparation of the manuscript. The specific roles of these authors are articulated in the ‘author contributions’ section.

4. Your ethics statement should only appear in the Methods section of your manuscript. If your ethics statement is written in any section besides the Methods, please delete it from any other section.

5. Please ensure that you refer to Figure 2 in your text as, if accepted, production will need this reference to link the reader to the figure.

6. We note that there is identifying data in the Supporting Information file “Supplementary material 4 additional tables.docx”. Due to the inclusion of these potentially identifying data, we have removed this file from your file inventory. Prior to sharing human research participant data, authors should consult with an ethics committee to ensure data are shared in accordance with participant consent and all applicable local laws.

Data sharing should never compromise participant privacy. It is therefore not appropriate to publicly share personally identifiable data on human research participants. The following are examples of data that should not be shared:

-Name, initials, physical address

-Ages more specific than whole numbers

-Internet protocol (IP) address

-Specific dates (birth dates, death dates, examination dates, etc.)

-Contact information such as phone number or email address

-Location data

-ID numbers that seem specific (long numbers, include initials, titled “Hospital ID”) rather than random (small numbers in numerical order)

Data that are not directly identifying may also be inappropriate to share, as in combination they can become identifying. For example, data collected from a small group of participants, vulnerable populations, or private groups should not be shared if they involve indirect identifiers (such as sex, ethnicity, location, etc.) that may risk the identification of study participants.

Additional guidance on preparing raw data for publication can be found in our Data Policy (https://journals.plos.org/plosone/s/data-availability#loc-human-research-participant-data-and-other-sensitive-data) and in the following article: http://www.bmj.com/content/340/bmj.c181.long.

Please remove or anonymize all personal information “ID numbers”, ensure that the data shared are in accordance with participant consent, and re-upload a fully anonymized data set. Please note that spreadsheet columns with personal information must be removed and not hidden as all hidden columns will appear in the published file.

7. We note that this data set consists of interview transcripts. Can you please confirm that all participants gave consent for interview transcript to be published?

If they DID provide consent for these transcripts to be published, please also confirm that the transcripts do not contain any potentially identifying information (or let us know if the participants consented to having their personal details published and made publicly available). We consider the following details to be identifying information:

- Names, nicknames, and initials

- Age more specific than round numbers

- GPS coordinates, physical addresses, IP addresses, email addresses

- Information in small sample sizes (e.g. 40 students from X class in X year at X university)

- Specific dates (e.g. visit dates, interview dates)

- ID numbers

Or, if the participants DID NOT provide consent for these transcripts to be published:

- Provide a de-identified version of the data or excerpts of interview responses

- Provide information regarding how these transcripts can be accessed by researchers who meet the criteria for access to confidential data, including:

a) the grounds for restriction

b) the name of the ethics committee, Institutional Review Board, or third-party organization that is imposing sharing restrictions on the data

c) a non-author, institutional point of contact that is able to field data access queries, in the interest of maintaining long-term data accessibility.

d) Any relevant data set names, URLs, DOIs, etc. that an independent researcher would need in order to request your minimal data set.

For further information on sharing data that contains sensitive participant information, please see: https://journals.plos.org/plosone/s/data-availability#loc-human-research-participant-data-and-other-sensitive-data

If there are ethical, legal, or third-party restrictions upon your dataset, you must provide all of the following details (https://journals.plos.org/plosone/s/data-availability#loc-acceptable-data-access-restrictions):

1. A complete description of the dataset

2. The nature of the restrictions upon the data (ethical, legal, or owned by a third party) and the reasoning behind them

3. The full name of the body imposing the restrictions upon your dataset (ethics committee, institution, data access committee, etc)

4. If the data are owned by a third party, confirmation of whether the authors received any special privileges in accessing the data that other researchers would not have

5. Direct, non-author contact information (preferably email) for the body imposing the restrictions upon the data, to which data access requests can be sent

8. If the reviewer comments include a recommendation to cite specific previously published works, please review and evaluate these publications to determine whether they are relevant and should be cited. There is no requirement to cite these works unless the editor has indicated otherwise.

[Note: HTML markup is below. Please do not edit.]

Reviewers' comments:

Reviewer's Responses to Questions

**Comments to the Author**

1. Is the manuscript technically sound, and do the data support the conclusions?

Reviewer #1: Partly

Reviewer #2: Partly

Reviewer #3: Yes

**********

2. Has the statistical analysis been performed appropriately and rigorously?

Reviewer #1: Yes

Reviewer #2: Yes

Reviewer #3: Yes

**********

3. Have the authors made all data underlying the findings in their manuscript fully available?

The PLOS Data policy

Reviewer #1: Yes

Reviewer #2: Yes

Reviewer #3: Yes

**********

4. Is the manuscript presented in an intelligible fashion and written in standard English?

Reviewer #1: Yes

Reviewer #2: Yes

Reviewer #3: Yes

**********

Reviewer #1: As the statistical reviewer I will focus on methods and reporting

Major

1) aspects of the paper are not easy to follow, with bullet points and not strong academic writing.

2) it was not clear what the primary outcome is in the relevant section (study outcomes). This is key since all other analyses for other outcomes need to be treated as exploratory since the power calculation does not relate to them.

3) specifically about the power calculation, there is no clarity of the primary outcome (clinical gain - needs to be defined in the main paper not just the appendix) and what that implies. how was clustering accounted for. and what level of attrition was accounted for. currently that is not replicable from what is reported in the main paper. Note that the hypothesised effect is very large and probably unrealistic - there is no justification on why this effect size is expected, e.g. from previous work.

4) practice needs to be included as a random intercept in the regression models to account for clustering.

5) it's not clear how missing data were dealt with. The consort statement has a section on that. were all data complete? if yes please state so. if not, why weren't multiple imputation approaches considered.

6) why was age and WIMD dichotomised? there is no rationale for this and it reduced power further.

7) there are baseline imbalances that the authors try to address through regression modelling - these need to be demonstrated in the relevant tables using SMDs. sex needs to be adjusted in all models, and also any other covariate with imblalanced needs to be included.

Minor

1) WIMD needs to be defined for international readers. area level measure, what domains it includes, what is the level on which calculated etc (Also what year used).

2) avoid the use of McNemar's test and any other test that focuses on statistical significance. the regression model is adequate for that, with the baseline scores/values included as covariates. this allows obtaining effect sizes and confidence intervals which are more informative than p-values, especially in analyses that are underpowrered (e.g. secondary outcocomes). This to some extent addresses the type-I error inflation - this needs to be discussed as a limitation and also all analyses except primary outcome need to be discussed as exploratory.

Reviewer #2: This is an interesting article, indeed a "better system" However, I am unable to access and review the ADRe profile itself, which I believe is the essence of this paper. The link should be easily accessible, or a version posted as supplementary material to enable a proper understanding of the applicability of such a tool.

Line 86- actual is underlined??

Different fonts and sizes in various parts of the article, these should be corrected.

Table 3- 1,11 or 1.11 . please clarify

Reviewer #3: 1.Cluster randomization concerns

The study is described as a cluster-randomized trial involving six GP practice clusters; however, only one cluster consisting of six practices was ultimately recruited. This substantially undermines the rationale for cluster randomization and raises concerns about selection bias and external validity. Please clarify whether clustering effects were accounted for and discuss the implications for statistical inference.

2. Deviation from the planned study design

The protocol planned recruitment of six clusters, but the final study included only one cluster. This represents a major deviation from the protocol and may invalidate the sample size calculation and power assumptions. The impact of this deviation should be explicitly discussed.

3. Unit of analysis vs. design mismatch

Although the trial was designed as a cluster RCT, the analysis was conducted at the individual patient level due to insufficient clusters. This raises concerns about inflated type I error and potential violation of the original trial design assumptions.

4. Lack of ADR causality assessment

The manuscript frequently attributes symptoms to medications, yet no formal causality assessment method (e.g., WHO-UMC, Naranjo) was used. The findings therefore represent symptom detection rather than confirmed ADR identification, and this distinction should be clearly stated.

5. Definition and clinical relevance of “problems”

The study reports 388 problems among 27 patients, averaging over 14 problems per patient, most of which were not recorded in medical records. It is unclear how a “problem” was defined and whether minor symptoms were counted alongside clinically significant ADRs. Greater clarification of problem classification and clinical relevance is required.

6. Potential overinterpretation of clinical benefit

The manuscript states that all patients in the intervention arm benefited or expected to benefit, which appears overly strong given that many interventions consisted of referrals or monitoring rather than confirmed clinical improvement. The interpretation of benefit should be more cautious and evidence-based.

7. Generalizability limitations

The study was conducted in a single economically deprived region with a very small number of participating practices. The generalizability of the findings to broader primary care populations requires further discussion.

8. Feasibility and implementation challenges

The study notes that many clinicians declined participation due to concerns about workload. This suggests potential implementation barriers, which should be more fully discussed in the context of real-world adoption.

9. Inconsistent age criteria

The abstract states >64 years, while the methods state ≥65 years. Please standardize the eligibility criteria.

10. Terminology clarity

The manuscript uses ADR, ADE, symptoms, and problems interchangeably. These represent distinct concepts in pharmacovigilance and should be used consistently.

11. Polypharmacy definition

Please clarify whether PRN medications were included in the count of long-term medicines.

12. Intervention standardization

Additional detail is needed on training of pharmacists and standardization of ADRe review procedures.

13. Outcome hierarchy

It would be helpful to distinguish clearly between process outcomes (e.g., referrals, investigations) and clinical outcomes (symptom resolution, deprescribing).

14. Phases of study

It is mentioned in the protocol that the study would be conducted in 3 phases. Wa it maintained while conducting the study?Please provide the timeline for the same.

**********

what does this mean?). If published, this will include your full peer review and any attached files.

If you choose “no”, your identity will remain anonymous but your review may still be made public.

**Do you want your identity to be public for this peer review?**  For information about this choice, including consent withdrawal, please see our Privacy Policy

Reviewer #1: No

Reviewer #2: No

Reviewer #3: **Yes:** Dr. Meenalotchini Prakash Gurunthalingam

**********

[NOTE: If reviewer comments were submitted as an attachment file, they will be attached to this email and accessible via the submission site. Please log into your account, locate the manuscript record, and check for the action link "View Attachments". If this link does not appear, there are no attachment files.]

To ensure your figures meet our technical requirements, please review our figure guidelines: https://journals.plos.org/plosone/s/figures

You may also use PLOS’s free figure tool, NAAS, to help you prepare publication quality figures: https://journals.plos.org/plosone/s/figures#loc-tools-for-figure-preparation.

NAAS will assess whether your figures meet our technical requirements by comparing each figure against our figure specifications.

# Author comment: Author response to Decision Letter 1

**DOI:** 10.1371/journal.pone.0356480.r002

7 May 2026

27.4.26

Dear Dr. Gebreyohannes,

“A better system” – managing the adverse effects of prescribed medicines: cluster-randomised controlled trial and process evaluation of the ADRe Profile in primary care

Short title: Monitoring patients for adverse effects of prescribed medicines

PONE-D-26-02770

Thank you for the opportunity to respond to the reviewers’ helpful comments. We have re-analysed the data and clarified as requested. Reponses to individual comments are tabulated.

We are grateful to the reviewers for their insightful comments.

While the reviewers recognise the potential relevance of your study, they have raised several important concerns that need to be addressed before the manuscript can be considered further.

In particular, the reviewers highlighted the following key issues among others:

• Academic writing and clarity: The manuscript would benefit from substantial editing to improve the clarity, structure, and overall academic writing. We apologise for any lack of clarity. The manuscript has been re-edited by native English speakers, who are editors of research journals. We are unable to identify either statements that need further support or grammatical errors.

• Primary outcome: The primary outcome of the study should be explicitly stated and consistently presented. We apologise for any lack of clarity. We have re-written (line 143 et seq):

The primary outcome was ‘health problems addressed’, as indicated by the medical records. ‘Health problems’ were defined as signs and symptoms listed on ADRe [46] [44 p.5], and S3 file. ‘Problems addressed’ were defined as those where action was taken following problem identification, for example by changes in prescribing or referrals or health promotion [44 p.5] (examples are described under ‘clinical gain’ and in S4 file, S2 Table). Before and after ADRe use, in both arms, using medical records, we assessed the number and nature of health problems identified and addressed and changes in prescribing, including deprescribing (see glossary), dose reduction, new prescriptions, laboratory tests, and recording of vital signs [55].

• Power calculation and clustering Thank you. We have moved the detail of the calculation from the published protocol [44], from supplementary material into the manuscript (line 163 et seq).

In a ‘before and after’ single arm intervention study, with participants acting as their own controls, 17/19 (89%) had at least one clinical problem addressed when ADRe for polypharmacy was used [8]. We hypothesised that 9/19 (47%) would have shown some improvement without intervention, necessitating an effective sample of 40 patients (20 in each arm), with 5% significance and 90% power [56]. Based on the reported intra-cluster coefficient (0.02), [43], and a design effect of 1.18, to achieve an effective sample size of 40, an actual sample size of 48 was needed (Killip et al 2004) [58]. Ten patients in each of 6 clusters (GP practices) allows for 20% loss to follow up (S3 file).

Thank you. The two uses of the term ‘clustering’ have been explicated. We apologise for any confusion. We have two distinct types of “clusters”. To avoid ambiguity. GP clusters have now been re-named GP groups, retaining the term ‘cluster’ solely as a statistical concept.

• Handling of missing data • Data collection from medical notes was complete (Tables 4, 5).

• We have detailed where missing data arose. We explain (line 219 et seq):

Missing data arose in the ADRe questionnaire when: a) participants completed ADRe by telephone or were unable to stand it was impossible to obtain measurements of all vital signs (5/27 respondents); b) one questions were designated as ‘discretionary’ due to cultural sensitivities: the question on sexual dysfunction was omitted by 15/27 respondents; all other questions were complete. These data were not subjected to inferential analyses. (Further details are in S3 file.) Differences between those responding and not responding militated against transferability of responses, and hence data imputation.•

Reasons for missing data

• Lack of equipment (e.g. scales, device battery)

• Participant physical limitations (e.g., inability to stand to measure standing blood pressure)

• Telephone-based completion

• Clinical discretion for sensitive items •

• The manuscript should clearly explain any deviations from the originally planned study design and discuss their potential implications. These were detailed in S3 file, and more details have now been added to the manuscript.

Protocol deviations are detailed in S3 file; most notably, time between the data collection rounds was more protracted than planned for 22/27 participants.

• Delays in follow-up timing (planned: 4-12 weeks), actual: 16 had follow-up at 17-20 weeks (causes: logistical delays

Please carefully revise your manuscript in light of the reviewers’ comments and provide a detailed, point-by-point response to each comment.

5. Review Comments to the Author

Reviewer #1: As the statistical reviewer I will focus on methods and reporting

Major

1) aspects of the paper are not easy to follow with bullet points and not strong academic writing. Thank you for this insightful comment. We have revisited the manuscript.

We did not identify any bullet points. The manuscript has been re-edited by three professors, all native English speakers, who are also editors. We did not identify any grammatical errors or unorthodox style or unsupported statements. We have aimed for appropriate intertextuality.

https://en.wikipedia.org/wiki/Academic_writing

2) it was not clear what the primary outcome is in the relevant section (study outcomes). This is key since all other analyses for other outcomes need to be treated as exploratory since the power calculation does not relate to them. Thank you. We have clarified (line 143 et seq):

A) The primary outcome was ‘health problems addressed’, as indicated by the medical records. ‘Health problems’ were defined as signs and symptoms listed on ADRe [46] [44 p.5], and S3 file. ‘Problems addressed’ were defined as those where action was taken following problem identification, for example by changes in prescribing or referrals or health promotion [44 p.5] (examples are described under ‘clinical gain’ and in S4 file, S2 Table). Before and after ADRe use, in both arms, using medical records, we assessed the number and nature of health problems identified and addressed and changes in prescribing, including deprescribing (see glossary), dose reduction, new prescriptions, laboratory tests, and recording of vital signs [55].

3) specifically about the power calculation e.g. from previous work. We have transferred the text from the protocol paper [44] from supplementary material to the manuscript. We have added (line 163 et seq):

In a ‘before and after’ single arm intervention study, with participants acting as their own controls, 17/19 (89%) had at least one clinical problem addressed when ADRe for polypharmacy was used [8]. We hypothesised that 9/19 (47%) would have shown some improvement without intervention, necessitating an effective sample of 40 patients (20 in each arm), with 5% significance and 90% power [56]. Based on the reported intra-cluster coefficient (0.02), [43], and a design effect of 1.18, to achieve an effective sample size of 40, an actual sample size of 48 was needed (Killip et al 2004) [58]. Ten patients in each of 6 clusters (GP practices) allows for 20% loss to follow up (S3 file).

4) practice needs to be included as a random intercept in the regression models to account for clustering. Thank you for this suggestion. The GP practice variable was included as a random intercept in all 5 GLMM analyses to allow for the multi-level structure of the data. Methods text now states ((line 187 et seq):

… assessed using multilevel generalized linear mixed models (GLMM), accounting for variability across GP practices by including practice as a random intercept [60]. We adjusted for age, deprivation and comorbidity. Data on age and deprivation were dichotomised: patients aged 65-75 (reference group) were compared with older patients; those in the most deprived WIMD quintile (reference group) were compared all with other patients, reflecting the study population. Comorbidity at baseline was summarised by ‘Number of long-term conditions’, entered as a continuous variable.

Table 5 has been reworked, replacing the previous adjusted analyses. Full models, as generated in spss, have been included in S4 file to ensure transparency.

Results were essentially unchanged when adjusted analyses from the GLMMs were substituted for the binary logistic regression models. As expected, confidence intervals widened.

Our professor of statistics has checked this addition to the paper, and is now included as a co-author.

5) it’s not clear how missing data were dealt with. The consort statement has a section on that. were all data complete? If yes please state so. If not why weren’t multiple imputation approaches considered. We agree this is a very important consideration, and apologise for any lack of clarity. We have further detailed where missing data arose. We explain (line 218 et seq):

Data collection from medical notes was complete. Missing data arose in the ADRe questionnaire when: a) participants completed ADRe by telephone or were unable to stand it was impossible to obtain measurements of all vital signs (5/27 respondents); b) one questions were designated as ‘discretionary’ due to cultural sensitivities: the question on sexual dysfunction was omitted by 15/27 respondents; all other questions were complete. These data were not subjected to inferential analyses. (Further details are in S3 file.) Differences between those responding and not responding militated against transferability of responses, and hence data imputation.

6) why was age and WIMD dichotomised? There is no rationale for this and it reduced power further. Thank you for highlighting this issue. We have now expanded the ‘limitations’ section of the manuscript to address concerns. As we acknowledge, the small sample size in our trial limited the deployment of covariates. We avoided low numbers in the cells of our models, as far as possible.

All participants were aged ≥65, indicating that a continuous variable would be inappropriate. In both arms, age was not normally distributed. We have added this information as a note to Table 1.

Shapiro- Wilk test for data distribution: intervention arm P=0.01; control arm P=0.01. We have also supported the use of 75 as a ‘cut point’ with a reference .

We have added a further note to Table 1:

Dichotomisation of age was based on definitions of premature death, used in UK statistics [62].

WIMD is a categorical variable. Most participants were in the most deprived quintile (Table 1). Numbers in the other 4 quintiles ranged between 4 and 0. Local knowledge of the research setting confirms the validity of this dichotomisation. We have added a note to Table 1:

There were 0-4 participants in each of the other four quintiles.

We agree that these measure to promote parsimony and minimise over-fitting reduce the power of the analyses. We now acknowledge this under ‘limitations’ (line 553 et seq).

Corollaries of the small sample size were low counts in some categories, necessitating simplification of variables, and restriction in the number of covariates.

And also any other covariate with imblalanced needs to be included. Thank you for highlighting this. We have augmented the ‘limitation’ section of the discussion to encompass this important consideration.

We are limited by the size of the data set, and the numbers of participants with each outcome. Our lived experience of valleys’ communities suggests that we have included the most important modifiable risk factors that might help practitioners to identify needs.

We have expanded (line 553 et seq)

Corollaries of the small sample size were low counts in some categories, necessitating simplification of variables, and restriction in the number of covariates. Covariates selected reflect the important considerations in our communities. The diverse domains of the WIMD obviate the necessity of considering each separately in small studies. The need to narrow confidence intervals and reduce the risk of type 1 errors by introducing further covariates was balanced against the risk of over-fitting, which would limit the validity of findings [99]. We acknowledge that our model, based on 57 participants, is vulnerable to over-interpretation and inclusion of secondary outcomes, without adjusting for multiple comparisons, increases the likelihood of reporting misleading positive results. However, statistical findings should be considered alongside the narrative accounts of patient benefit when determining the practical adequacy of ADRe [100]

Minor

11) WIMD needs to be defined for international readers. Area level measure

what domains it includes

what is the level on which calculated etc (Also what year used). Thank you, we have augmented the text accordingly (line 174 et seq):

The Wales 2019 Index of Multiple Deprivation (WIMD) is a composite of eight domains (or types) of deprivation: a) Income b) Employment c) Health d) Education e) Access to Services f) Community Safety g) Physical Environment h) Housing. Each domain is compiled from several different indicators, and the domains are combined to give a single rank for the local area [59].

2) avoid the use of McNemar’s test and any other test that focuses on statistical significance. The regression model is adequate for that

with the baseline scores/values included as covariates. This allows obtaining effect sizes and confidence intervals which are more informative than p-values

especially in analyses that are underpowrered (e.g. secondary outcocomes). This to some extent addresses the type-I error inflation – this needs to be discussed as a limitation and also all analyses except primary outcome need to be discussed as exploratory. Thank you. The testing of symptoms between before and after the intervention cannot be accommodated in the regression models because we do not have information on the signs and symptoms listed on ADRe for the control arm. The number of long-term conditions listed in the medical records is a covariate.

We acknowledge the limitations of McNemar’s test, and, on the advice of the reviewer, have removed it from the paper.

All trial outcomes were pre-specified in the protocol [44].

We agree that secondary outcomes inflate the risk of type I errors, and, as suggested, added this point to the ‘limitations’ (line 558 et seq):

We acknowledge that our model, based on 57 participants, is vulnerable to over-interpretation and inclusion of secondary outcomes, without adjusting for multiple comparisons, increases the likelihood of reporting misleading positive results. However, statistical findings should be considered alongside the narrative accounts of patient benefit when determining the practical adequacy of ADRe [100].

Reviewer #2: This is an interesting article, indeed a “better system”

However, I am unable to access and review the ADRe profile itself, which I believe is the essence of this paper. The link should be easily accessible, or a version posted as supplementary material to enable a proper understanding of the applicability of such a tool.

Thank you. Positive support is much appreciated.

We apologise. ADRe is normally available by email from our website. A direct link was included in reference 46. We have now clarified and augmented reference [46]. The problems listed on ADRe are reproduced in S3 file. This has prompted us to expand the information in S4 file, S2 table.

Line 86- actual is underlined??

Thank you. This was intended for emphasis. Following reviewer’s advice, the underline has been removed.

Different fonts and sizes in various parts of the article, these should be corrected. Thank you. We have attended to this.

Table 3- 1,11 or 1.11 . please clarify Thank you for the detailed scrutiny. Typographical error. We apologise.

Reviewer #3:

1.Cluster randomization concerns

The study is described as a cluster-randomized trial involving six GP practice clusters; howeve

Attachment Submitted filename: RCT Response to reviewers and editor (2).docx

# Peer review: Decision Letter 1

**DOI:** 10.1371/journal.pone.0356480.r003

**Authors:** Eyob Alemayehu Gebreyohannes

11 Jun 2026

Dear Dr. Jordan,

Thank you for submitting your manuscript to PLOS ONE. After careful consideration, we feel that it has merit but does not fully meet PLOS ONE’s publication criteria as it currently stands. Therefore, we invite you to submit a revised version of the manuscript that addresses the points raised during the review process.

Please submit your revised manuscript by Jul 26 2026 11:59PM. If you will need more time than this to complete your revisions, please reply to this message or contact the journal office at plosone@plos.org. When you're ready to submit your revision, log on to https://www.editorialmanager.com/pone/ and select the 'Submissions Needing Revision' folder to locate your manuscript file.

- A letter that responds to each point raised by the academic editor and reviewer(s). You should upload this letter as a separate file labeled 'Response to Reviewers'.
- A marked-up copy of your manuscript that highlights changes made to the original version. You should upload this as a separate file labeled 'Revised Manuscript with Track Changes'.
- An unmarked version of your revised paper without tracked changes. You should upload this as a separate file labeled 'Manuscript'.

If you would like to make changes to your financial disclosure, please include your updated statement in your cover letter. Guidelines for resubmitting your figure files are available below the reviewer comments at the end of this letter.

If applicable, we recommend that you deposit your laboratory protocols in protocols.io to enhance the reproducibility of your results. Protocols.io assigns your protocol its own identifier (DOI) so that it can be cited independently in the future. For instructions see: https://journals.plos.org/plosone/s/submission-guidelines#loc-laboratory-protocols. Additionally, PLOS ONE offers an option for publishing peer-reviewed Lab Protocol articles, which describe protocols hosted on protocols.io. Read more information on sharing protocols at https://plos.org/protocols?utm_medium=editorial-email&utm_source=authorletters&utm_campaign=protocols.

As the corresponding author, your ORCID iD is verified in the submission system and will appear in the published article. PLOS supports the use of ORCID, and we encourage all coauthors to register for an ORCID iD and use it as well. Please encourage your coauthors to verify their ORCID iD within the submission system before final acceptance, as unverified ORCID iDs will not appear in the published article. *Only*  the individual author can complete the verification step; PLOS staff *cannot*  verify ORCID iDs on behalf of authors.

We look forward to receiving your revised manuscript.

Kind regards,

Eyob Alemayehu Gebreyohannes, PhD

Academic Editor

PLOS One

**Journal Requirements:**

1. If the reviewer comments include a recommendation to cite specific previously published works, please review and evaluate these publications to determine whether they are relevant and should be cited. There is no requirement to cite these works unless the editor has indicated otherwise.

2. Please review your reference list to ensure that it is complete and correct. If you have cited papers that have been retracted, please include the rationale for doing so in the manuscript text, or remove these references and replace them with relevant current references. Any changes to the reference list should be mentioned in the rebuttal letter that accompanies your revised manuscript. If you need to cite a retracted article, indicate the article’s retracted status in the References list and also include a citation and full reference for the retraction notice.

**Additional Editor Comments:**

Revisit the reference to the 24 GP groups, as it may be unnecessary and potentially confusing for readers. Instead, rephrase the terminology and, where possible, provide a clearer description of the specific cluster included in the study. This would improve clarity and maintain focus on the study findings without introducing distracting detail.

[Note: HTML markup is below. Please do not edit.]

Reviewers' comments:

Reviewer's Responses to Questions

**Comments to the Author**

Reviewer #1: All comments have been addressed

Reviewer #2: (No Response)

Reviewer #3: All comments have been addressed

**********

2. Is the manuscript technically sound, and do the data support the conclusions?

Reviewer #1: Yes

Reviewer #2: Yes

Reviewer #3: Yes

**********

3. Has the statistical analysis been performed appropriately and rigorously?

Reviewer #1: Yes

Reviewer #2: I Don't Know

Reviewer #3: Yes

**********

4. Have the authors made all data underlying the findings in their manuscript fully available?

The PLOS Data policy

Reviewer #1: Yes

Reviewer #2: Yes

Reviewer #3: Yes

**********

5. Is the manuscript presented in an intelligible fashion and written in standard English?

Reviewer #1: Yes

Reviewer #2: Yes

Reviewer #3: Yes

**********

**Reviewer #1:**  I am satisfied with the authors' responses and the resulting changes to the paper. I have nothing else to add.

**Reviewer #2:** I think the reference to the 24 GP groups is not necessary and confusing, the authors should rephrase the terminology and describe if possible, the particular cluster that was used in the study. This does not distract from the findings.

**Reviewer #3:** Thank you to the authors for the careful and constructive revision of the manuscript. The revised version is notably improved in terms of methodological clarity, statistical reporting, and discussion of limitations. I especially appreciate the re-analysis using GLMM to account for clustering effects, as well as the clearer distinction made between patient-reported symptoms/problems and confirmed adverse drug reactions.

The manuscript addresses an important and clinically relevant area in primary care pharmacovigilance and polypharmacy management among older adults. The patient-centred approach of the intervention, together with its emphasis on communication between patients, pharmacists, and prescribers, represents a meaningful strength of the study.

The additional clarifications regarding study design, terminology, handling of PRN medications, and differentiation between process and clinical outcomes have improved both the readability and interpretability of the manuscript. I also value the authors’ more balanced discussion of implementation challenges and feasibility in routine clinical practice.

A few concerns nevertheless remain, mainly related to the interpretation and framing of the findings. While the revised statistical approach is appropriate, the small number of participating practices and recruitment from a single GP group continue to limit the robustness and wider generalizability of the conclusions. In this regard, the study may be better framed as exploratory or pilot in nature.

In addition, some statements relating to “clinical benefit” and “improved outcomes” still appear somewhat stronger than the available evidence fully supports. Several of the reported benefits were linked to identification of symptoms, referrals, investigations, medication review, or monitoring processes, rather than confirmed reductions in medication-related harm. I would therefore encourage the authors to further moderate causal language throughout the manuscript and to consistently distinguish between:

-patient-reported symptoms/problems,

-potential medication-related concerns,

-and confirmed ADRs/ADEs.

In Table 3, terms such as “after intervention” may be slightly confusing when referring to the control arm, since the control group did not receive the intervention. Consider using terminology such as “follow-up” or “post-study assessment” for clarity.

In Table 5, the distinction between “Deprescribing, medicines discontinued” and “Deprescribing, medicines stopped or doses reduced” is not immediately clear and may be confusing to readers, as the latter appears to include the former. Kindly clarify whether these represent separate or cumulative outcomes and consider revising the wording for greater clarity.

In the response to reviewers, it is mentioned that only one pharmacist was involved in the study/intervention. However, in the Results section (process evaluation), references are made to “Pharmacist 1” and “Pharmacist 2.” Kindly clarify the number and roles of pharmacists involved in the intervention and process evaluation, as this currently appears inconsistent and may confuse readers.

Overall, this is a thoughtful and clinically valuable study that contributes meaningfully to ongoing discussions on safer prescribing and person-centred monitoring in primary care. With some further refinement of the conclusions and interpretation, the manuscript will be further strengthened.

**********

what does this mean?). If published, this will include your full peer review and any attached files.

If you choose “no”, your identity will remain anonymous but your review may still be made public.

**Do you want your identity to be public for this peer review?**  For information about this choice, including consent withdrawal, please see our Privacy Policy

Reviewer #1: No

Reviewer #2: No

Reviewer #3: No

**********

[NOTE: If reviewer comments were submitted as an attachment file, they will be attached to this email and accessible via the submission site. Please log into your account, locate the manuscript record, and check for the action link "View Attachments". If this link does not appear, there are no attachment files.]

To ensure your figures meet our technical requirements, please review our figure guidelines: https://journals.plos.org/plosone/s/figures

You may also use PLOS’s free figure tool, NAAS, to help you prepare publication quality figures: https://journals.plos.org/plosone/s/figures#loc-tools-for-figure-preparation.

NAAS will assess whether your figures meet our technical requirements by comparing each figure against our figure specifications.

# Author comment: Author response to Decision Letter 2

**DOI:** 10.1371/journal.pone.0356480.r004

1 Jul 2026

Dear Dr. Gebreyohannes,

Thank you very much for considering our work. Our responses are below.

Yours,

Sue

Journal Requirements:

1. If the reviewer comments include a recommendation to cite specific previously published works, please review and evaluate these publications to determine whether they are relevant and should be cited. There is no requirement to cite these works unless the editor has indicated otherwise. NA

2. Please review your reference list to ensure that it is complete and correct. If you have cited papers that have been retracted, please include the rationale for doing so in the manuscript text, or remove these references and replace them with relevant current references. Any changes to the reference list should be mentioned in the rebuttal letter that accompanies your revised manuscript. If you need to cite a retracted article, indicate the article’s retracted status in the References list and also include a citation and full reference for the retraction notice. NA

Additional Editor Comments:

Revisit the reference to the 24 GP groups, as it may be unnecessary and potentially confusing for readers. Instead, rephrase the terminology and, where possible, provide a clearer description of the specific cluster included in the study. This would improve clarity and maintain focus on the study findings without introducing distracting detail.

Thank you for this advice. I hope our amendments provide greater clarity. Our description has been curtailed by our commitment to ensure that the practices remain anonymous.

( Line 117 et seq)

We recruited 60 patients from six primary care practices in one valley in South-West Wales where the pharmacist and general practitioners (GPs) were willing to review completed ADRe documents. The six practices worked as a group of collaborating doctors and allied professionals. The study area is amongst the most deprived in Wales for income, employment and health; however, it enjoys relatively good transport links. Over half the patients are amongst the 20% most deprived in terms of health, measured as long-term illness, premature death, cancer incidence, recorded long-term and mental health conditions, preterm birth and childhood obesity (RCGP 2022, Welsh government 2025). The practices in the upper valley were the most deprived.

[Note: HTML markup is below. Please do not edit.]

Reviewers' comments:

Reviewer's Responses to Questions

Comments to the Author

Questions 1-5 are all affirmative, and do not require a response from us.

________________________________________

________________________________________

6. Review Comments to the Author

Please use the space provided to explain your answers to the questions above. You may also include additional comments for the author, including concerns about dual publication, research ethics, or publication ethics. (Please upload your review as an attachment if it exceeds 20,000 characters)

1. Reviewer #1: I am satisfied with the authors' responses and the resulting changes to the paper. I have nothing else to add. Thank you for your support.

2. Reviewer #2: I think the reference to the 24 GP groups is not necessary and confusing, the authors should rephrase the terminology and describe if possible, the particular cluster that was used in the study. This does not distract from the findings. Thank you for this insight. We have rewritten, line 117 et seq:

We recruited 60 patients from six primary care practices in one valley in South-West Wales where the pharmacist and general practitioners (GPs) were willing to review completed ADRe documents. The six practices worked as a group of collaborating doctors and allied professionals. The study area is amongst the most deprived in Wales for income, employment and health; however, it enjoys relatively good transport links. Over half the patients are amongst the 20% most deprived in terms of health, measured as long-term illness, premature death, cancer incidence, recorded long-term and mental health conditions, preterm birth and childhood obesity (RCGP 2022, Welsh government 2025). The practices in the upper valley were the most deprived.

Our description has been curtailed by our commitment to ensure that the practices remain anonymous.

3. Reviewer #3: Thank you to the authors for the careful and constructive revision of the manuscript. The revised version is notably improved in terms of methodological clarity, statistical reporting, and discussion of limitations. I especially appreciate the re-analysis using GLMM to account for clustering effects, as well as the clearer distinction made between patient-reported symptoms/problems and confirmed adverse drug reactions. Thank you for your support.

4. The manuscript addresses an important and clinically relevant area in primary care pharmacovigilance and polypharmacy management among older adults. The patient-centred approach of the intervention, together with its emphasis on communication between patients, pharmacists, and prescribers, represents a meaningful strength of the study. Thank you for your support.

5. The additional clarifications regarding study design, terminology, handling of PRN medications, and differentiation between process and clinical outcomes have improved both the readability and interpretability of the manuscript. I also value the authors’ more balanced discussion of implementation challenges and feasibility in routine clinical practice. Thank you for your support.

6. A few concerns nevertheless remain, mainly related to the interpretation and framing of the findings. While the revised statistical approach is appropriate, the small number of participating practices and recruitment from a single GP group continue to limit the robustness and wider generalizability of the conclusions. In this regard, the study may be better framed as exploratory or pilot in nature. We agree that this trial is exploratory in primary care. We have substituted ‘explored’ for ‘investigated’ (line 109) and described our models as ‘exploratory’ (line 563).

There is no funding for further work, so we are unable to term this ‘pilot work’. We acknowledge that ‘further work on implementation of these exploratory findings is needed.’ (line 604)

The evidence for adoption of ADRe becomes more convincing when considered alongside the work in other areas (lines 91-2, and 612), and patients’ views (line 504)

7. In addition, some statements relating to “clinical benefit” and “improved outcomes” still appear somewhat stronger than the available evidence fully supports. Several of the reported benefits were linked to identification of symptoms, referrals, investigations, medication review, or monitoring processes, rather than confirmed reductions in medication-related harm. I would therefore encourage the authors to further moderate causal language throughout the manuscript and to consistently distinguish between: We have endeavoured to comply, as detailed below.

We agree that referrals do not always confer clinical benefit; however, expert opinion is welcomed by patients, and care usually improves. We now acknowledge that outcomes of referrals (mainly to medical specialists) were not available within the study timeframe. Line 590 et seq now reads:

The study’s timeframe limited exploration of all associations between symptoms and medicines identified in ADRe decision support and the outcomes of referrals to secondary care.

We do not mention this is the paper, but our participants were unable to expedite their referrals to consultants by paying for private healthcare, leading to long delays.

We have qualified our claims by inserting ‘some’, for example, line 599:

This addressed some instances of unmonitored avoidable medicine-related harm …

8. -patient-reported symptoms/problems, We agree that ADRe only monitors patient-reported problems. Where patients live in their own homes, it is impossible to confirm reports of falls or insomnia. To clarify, we have changed the section heading from ‘clinical gain’ to ‘clinical gains reported by patients’. (line 350)

We now acknowledge that patient-reported symptoms fluctuate for reasons unrelated to pharmacotherapy. Line 454:

Other gains were more modest e.g. relief of itching following prescription for an emollient (ID 9), and some symptoms abated for no reason apparent to researchers e.g. insomnia and vivid dreams subsided (ID 2).

For the primary outcome, we used the medical notes. While we can be confident regarding actions taken and prescription changes, we can only infer associations with patient-reported problems.

9. -potential medication-related concerns, In practice, it is difficult to assign aetiology, as noted (line 577):

Symptom aetiology was often uncertain or mixed.

By listing putative explanations for resolution of problems, Table 6 shows that many improvements were not due to changes in prescriptions.

10. -and confirmed ADRs/ADEs. We agree and acknowledge (line 58) that we were unable to meet the criteria of de-challenge and re-challenge. Neither we nor the research ethics committee consider re-challenge to be appropriate in primary care trials of complex interventions, due to the risks involved. We acknowledge under ‘limitations’, line 577:

The provenance of problems identified was not explored with de-challenge and re-challenge, the ‘gold standard’ for ADR detection.

For some problems the balance of opinion indicates iatrogenic aetiology. Examples include: resolution of paraesthesia following B12 supplementation for a patient using metformin long-term, resolution of hallucinations on discontinuation of flecainide, resolution of abnormal movements following mycophenolate or gabapentin dose reduction.

11. In Table 3, terms such as “after intervention” may be slightly confusing when referring to the control arm, since the control group did not receive the intervention. Consider using terminology such as “follow-up” or “post-study assessment” for clarity. Thank you for this detailed consideration. We agree. We have adopted the terms ‘baseline’ and ‘follow-up’.

We have added a note to table 3:

* Baseline data were extracted before the intervention was delivered. Follow-up data were extracted after the intervention was delivered in the intervention arm. Control arm participants received usual care throughout; they did not receive the intervention.

We have adjusted line 99 et seq to reflect the change in terminology:

We explored: differences between trial arms in problems and prescriptions in the practice medical records at baseline and follow-up (before and after the intervention was delivered in one arm);

We have also clarified in line 157:

In both arms, at baseline (before) and follow-up (after ADRe use in one arm), using medical records …

12. In Table 5, the distinction between “Deprescribing, medicines discontinued” and “Deprescribing, medicines stopped or doses reduced” is not immediately clear and may be confusing to readers, as the latter appears to include the former. Kindly clarify whether these represent separate or cumulative outcomes and consider revising the wording for greater clarity. Thank you. We have added a note to table 5.

† This outcome measured both discontinued medicines (as above) plus dose reductions.

13. In the response to reviewers, it is mentioned that only one pharmacist was involved in the study/intervention. However, in the Results section (process evaluation), references are made to “Pharmacist 1” and “Pharmacist 2.” Kindly clarify the number and roles of pharmacists involved in the intervention and process evaluation, as this currently appears inconsistent and may confuse readers. Thank you. We have clarified that both pharmacists were working with the research team.

Line 209:

… two pharmacists working with the ADRe project [Logan et al 2025, 2026] …

We are obliged to restrict details to preserve anonymity.

Transcripts from all interviews in all phases of the project are available via a non-author email: library-digital@swansea.ac.uk or the corresponding authors.e.jordan@swansea.ac.uk.

URL is: https://zenodo.org/records/19736675

Doi is: 10.5281/zenodo.19736674 (https://doi.org/10.5281/zenodo.19736674)

Overall, this is a thoughtful and clinically valuable study that contributes meaningfully to ongoing discussions on safer prescribing and person-centred monitoring in primary care. With some further refinement of the conclusions and interpretation, the manuscript will be further strengthened. Thank you for your support. We have endeavoured to adopt the helpful suggestions of the expert reviewers. For example, line 613 now reads:

ADRe … reduces health-related problems for many patients …

Attachment Submitted filename: Response to reviews 11.6.26.docx

# Peer review: Decision Letter 2

**DOI:** 10.1371/journal.pone.0356480.r005

**Authors:** Eyob Alemayehu Gebreyohannes

27 Jul 2026

Dear Dr. Jordan,

Thank you for submitting your manuscript to PLOS ONE. After careful consideration, we feel that it has merit but does not fully meet PLOS ONE’s publication criteria as it currently stands. Therefore, we invite you to submit a revised version of the manuscript that addresses the points raised during the review process.

==============================

**ACADEMIC EDITOR:**

There is only one minor edits you need to make before the paper can be formally accepted for publication. In the abstract section, change "We recruited 1 of 24 general practice groups (each comprising 6 practices) and 60 of 170 patients 40 approached." To enhance clarity, re-write it as "We recruited 1 general practice group comprising 6 practices and 60 patients."

Please submit your revised manuscript by Sep 10 2026 11:59PM If you will need more time than this to complete your revisions, please reply to this message or contact the journal office at plosone@plos.org. When you're ready to submit your revision, log on to https://www.editorialmanager.com/pone/ and select the 'Submissions Needing Revision' folder to locate your manuscript file.

- A letter that responds to each point raised by the academic editor and reviewer(s). You should upload this letter as a separate file labeled 'Response to Reviewers'.
- A marked-up copy of your manuscript that highlights changes made to the original version. You should upload this as a separate file labeled 'Revised Manuscript with Track Changes'.
- An unmarked version of your revised paper without tracked changes. You should upload this as a separate file labeled 'Manuscript'.

If you would like to make changes to your financial disclosure, please include your updated statement in your cover letter. Guidelines for resubmitting your figure files are available below the reviewer comments at the end of this letter.

If applicable, we recommend that you deposit your laboratory protocols in protocols.io to enhance the reproducibility of your results. Protocols.io assigns your protocol its own identifier (DOI) so that it can be cited independently in the future. For instructions see: https://journals.plos.org/plosone/s/submission-guidelines#loc-laboratory-protocols. Additionally, PLOS ONE offers an option for publishing peer-reviewed Lab Protocol articles, which describe protocols hosted on protocols.io. Read more information on sharing protocols at https://plos.org/protocols?utm_medium=editorial-email&utm_source=authorletters&utm_campaign=protocols.

As the corresponding author, your ORCID iD is verified in the submission system and will appear in the published article. PLOS supports the use of ORCID, and we encourage all coauthors to register for an ORCID iD and use it as well. Please encourage your coauthors to verify their ORCID iD within the submission system before final acceptance, as unverified ORCID iDs will not appear in the published article. *Only*  the individual author can complete the verification step; PLOS staff *cannot*  verify ORCID iDs on behalf of authors.

We look forward to receiving your revised manuscript.

Kind regards,

Eyob Alemayehu Gebreyohannes, PhD

Academic Editor

PLOS One

Journal Requirements:

If the reviewer comments include a recommendation to cite specific previously published works, please review and evaluate these publications to determine whether they are relevant and should be cited. There is no requirement to cite these works unless the editor has indicated otherwise.

Please review your reference list to ensure that it is complete and correct. If you have cited papers that have been retracted, please include the rationale for doing so in the manuscript text, or remove these references and replace them with relevant current references. Any changes to the reference list should be mentioned in the rebuttal letter that accompanies your revised manuscript. If you need to cite a retracted article, indicate the article’s retracted status in the References list and also include a citation and full reference for the retraction notice.

Additional Editor Comments:

There is only one minor edits you need to make before the paper can be formally accepted for publication. In the abstract section, change "We recruited 1 of 24 general practice groups (each comprising 6 practices) and 60 of 170 patients 40 approached." To enhance clarity, re-write it as "We recruited 1 general practice group comprising 6 practices and 60 patients."

[Note: HTML markup is below. Please do not edit.]

Reviewers' comments:

Reviewer's Responses to Questions

**Comments to the Author**

Reviewer #1: All comments have been addressed

Reviewer #2: All comments have been addressed

**********

2. Is the manuscript technically sound, and do the data support the conclusions?

Reviewer #1: Yes

Reviewer #2: Yes

**********

3. Has the statistical analysis been performed appropriately and rigorously?

Reviewer #1: Yes

Reviewer #2: Yes

**********

4. Have the authors made all data underlying the findings in their manuscript fully available?

The PLOS Data policy

Reviewer #1: Yes

Reviewer #2: Yes

**********

5. Is the manuscript presented in an intelligible fashion and written in standard English?

Reviewer #1: Yes

Reviewer #2: Yes

**********

Reviewer #1: nothing else to add.........................................................................................................

Reviewer #2: The 1The abstract section needs to revised as well, particularly the methodology which still includes 1 in 24 practices. This needs clarification.

**********

what does this mean?). If published, this will include your full peer review and any attached files.

If you choose “no”, your identity will remain anonymous but your review may still be made public.

**Do you want your identity to be public for this peer review?**  For information about this choice, including consent withdrawal, please see our Privacy Policy

Reviewer #1: No

Reviewer #2: No

**********

[NOTE: If reviewer comments were submitted as an attachment file, they will be attached to this email and accessible via the submission site. Please log into your account, locate the manuscript record, and check for the action link "View Attachments". If this link does not appear, there are no attachment files.]

To ensure your figures meet our technical requirements, please review our figure guidelines: https://journals.plos.org/plosone/s/figures

You may also use PLOS’s free figure tool, NAAS, to help you prepare publication quality figures: https://journals.plos.org/plosone/s/figures#loc-tools-for-figure-preparation.

NAAS will assess whether your figures meet our technical requirements by comparing each figure against our figure specifications.

# Author comment: Author response to Decision Letter 3

**DOI:** 10.1371/journal.pone.0356480.r006

29 Jul 2026

There is only one minor edits you need to make before the paper can be formally accepted for publication. In the abstract section, change "We recruited 1 of 24 general practice groups (each comprising 6 practices) and 60 of 170 patients 40 approached." To enhance clarity, re-write it as "We recruited 1 general practice group comprising 6 practices and 60 patients."

Thank you, we have made the necessary change.

Thank you for your help and support.

we have uploaded a reformatted version of fig 1. The figure;s content is unchanged.

Attachment Submitted filename: Response to reviewers 28.7.26.docx

# Editor report: Decision Letter 3

**DOI:** 10.1371/journal.pone.0356480.r007

**Authors:** Eyob Alemayehu Gebreyohannes

4 Aug 2026

“A better system” – managing the adverse effects of prescribed medicines: cluster-randomised controlled trial and process evaluation of the ADRe Profile in primary care

PONE-D-26-02770R3

Dear Dr. Jordan,

We’re pleased to inform you that your manuscript has been judged scientifically suitable for publication and will be formally accepted for publication once it meets all outstanding technical requirements.

Within one week, you’ll receive an e-mail detailing the required amendments. When these have been addressed, you’ll receive a formal acceptance letter and your manuscript will be scheduled for publication.

An invoice will be generated when your article is formally accepted. Please note, if your institution has a publishing partnership with PLOS and your article meets the relevant criteria, all or part of your publication costs will be covered. Please make sure your user information is up-to-date by logging into Editorial Manager at Editorial Manager® and clicking the ‘Update My Information' link at the top of the page. For questions related to billing, please contact billing support.

If your institution or institutions have a press office, please notify them about your upcoming paper to help maximize its impact. If they’ll be preparing press materials, please inform our press team as soon as possible -- no later than 48 hours after receiving the formal acceptance. Your manuscript will remain under strict press embargo until 2 pm Eastern Time on the date of publication. For more information, please contact onepress@plos.org.

Kind regards,

Eyob Alemayehu Gebreyohannes, PhD

Academic Editor

PLOS One

# Editor report: Acceptance letter

**DOI:** 10.1371/journal.pone.0356480.r008

**Authors:** Eyob Alemayehu Gebreyohannes

PONE-D-26-02770R3

PLOS One

Dear Dr. Jordan,

I'm pleased to inform you that your manuscript has been deemed suitable for publication in PLOS One. Congratulations! Your manuscript is now being handed over to our production team.

At this stage, our production department will prepare your paper for publication. This includes ensuring the following:

* All references, tables, and figures are properly cited

* All relevant supporting information is included in the manuscript submission,

* There are no issues that prevent the paper from being properly typeset

You will receive further instructions from the production team, including instructions on how to review your proof when it is ready. Please keep in mind that we are working through a large volume of accepted articles, so please give us a few days to review your paper and let you know the next and final steps.

Lastly, if your institution or institutions have a press office, please let them know about your upcoming paper now to help maximize its impact. If they'll be preparing press materials, please inform our press team within the next 48 hours. Your manuscript will remain under strict press embargo until 2 pm Eastern Time on the date of publication. For more information, please contact onepress@plos.org.

You will receive an invoice from PLOS for your publication fee after your manuscript has reached the completed accept phase. If you receive an email requesting payment before acceptance or for any other service, this may be a phishing scheme. Learn how to identify phishing emails and protect your accounts at https://explore.plos.org/phishing.

If we can help with anything else, please email us at customercare@plos.org.

Thank you for submitting your work to PLOS One and supporting open access.

Kind regards,

PLOS One Editorial Office Staff

on behalf of

Dr. Eyob Alemayehu Gebreyohannes

Academic Editor

PLOS One
